# Types of Nursing Intervention on Improving Quality of Life among Patients with Diabetes Mellitus: A Systematic Review

**DOI:** 10.2174/1573399820666230829103016

**Published:** 2024-01-30

**Authors:** Agung Samsu Alam, Amin Samiasih, Mohammad Fatkhul Mubin, Satriya Pranata, Reina Dhamanik

**Affiliations:** 1 Department of Nursing, Faculty of Nursing and Health Sciences, Universitas Muhammadiyah Semarang, Semarang City, Central Java, Indonesia

**Keywords:** Diabetes mellitus, nursing interventions, quality of life, RCT, QoL, psychological abilities

## Abstract

**Background:**

Long-term treatment of patients with diabetes mellitus (DM) is considered a major factor causing disease complications. DM complications mostly impact the patient’s quality of life (QoL). Only a few studies have been conducted summarizing the types of nursing interventions for improving the QoL of DM patients.

**Objective:**

The objective of this study is to explore the types of nursing interventions that can improve the QoL of DM patients.

**Methods:**

The online databases, including ScienceDirect, Medline, Google Search, and Pro-Quest, were used to search for the relevant articles. Articles that met the inclusion criteria were analyzed, and their level of evidence was determined and synthesized.

**Results:**

A total of 30 articles defining the types of nursing intervention on improving the QoL of DM patients were discovered, comprising the five types of nursing interventions, such as health education (15 articles), exercise (8 articles), WhatsApp/short message service (WA/SMS) gateway (3 articles), blood glucose control (3 articles), and black garlic herbal therapy (1 article).

**Conclusion:**

Sequentially, the most common types of nursing interventions to improve the QoL of DM patients was health education, followed by exercise, WA/SMS gateway, and glucose control. A personal approach to health education is a significant point in improving the QoL of DM patients in the future. The findings of this study might not be strongly generalized, so further randomized controlled trial (RCT) studies with larger samples are needed.

## 1 INTRODUCTION

Diabetes Mellitus (DM) is considered one of the most prevalent chronic diseases worldwide. Globally, a total of 422 million DM patients were recorded in 2022 (an increase of 28.6% compared to the previous year), and it is predicted to be elevated in 2045 with a total number of 629 million cases. An increasing rate of DM cases has also been witnessed in Indonesia. A total of 19.5 million DM cases were reported in 2021 (an increase of 28.6% from 2020), and it is estimated to increase to 183 million in 2045 [1]. The increasing prevalence of DM indicates the need for prevention efforts. These prevention efforts aim to control blood glucose levels through four pillars of DM management, such as education, meal planning, physical exercise, and pharmacological therapy [2]. Several etiologies contribute to DM prevalence, including obesity, low physical activity, sugar levels in the body, stress, genetics, and the aging process (elderly). A previous study supported that a higher percentage of DM complications are triggered by obesity (53.6%), low physical activity (50.7%), and higher fasting sugar levels (75.4%) [3]. Numerous efforts have been implemented to prevent DM complications and maintain blood sugar within the normal range. The Indonesia Ministry of Health has initiated a healthy living program for DM patients by maintaining a diet style. A good diet style will improve the quality of life (QoL) of DM patients [4].

Patients with DM potentially encounter a decrease in QoL and contribute to decreasing *health-related quality of life* (HRQoL). It occurs due to limited physical, psychological, and cognitive abilities in performing daily activities. To date, many studies have been investigated to improve the QoL of DM patients. A recent study conducted in 2020 described that health education intervention is effective in improving the QoL of DM patients [5]. The previous investigation also explored that exercise, blood glucose level management, and black garlic consumption can also improve the QoL of DM patients [6]. Several studies mentioned above are still categorized as single studies. There is no systematic review that discusses the types of nursing interventions for increasing the QoL in DM patients. Thus, it is necessary to conduct a systematic literature study to determine the types of nursing interventions that contribute to increasing the QoL among DM patients. Therefore, the purpose of this study was to explore and review the current evidence regarding the types of nursing interventions that contribute to improve the QoL among patients with DM.

## 2 MATERIALS AND METHODS

### 2.1 Design

**Fig. (1):**
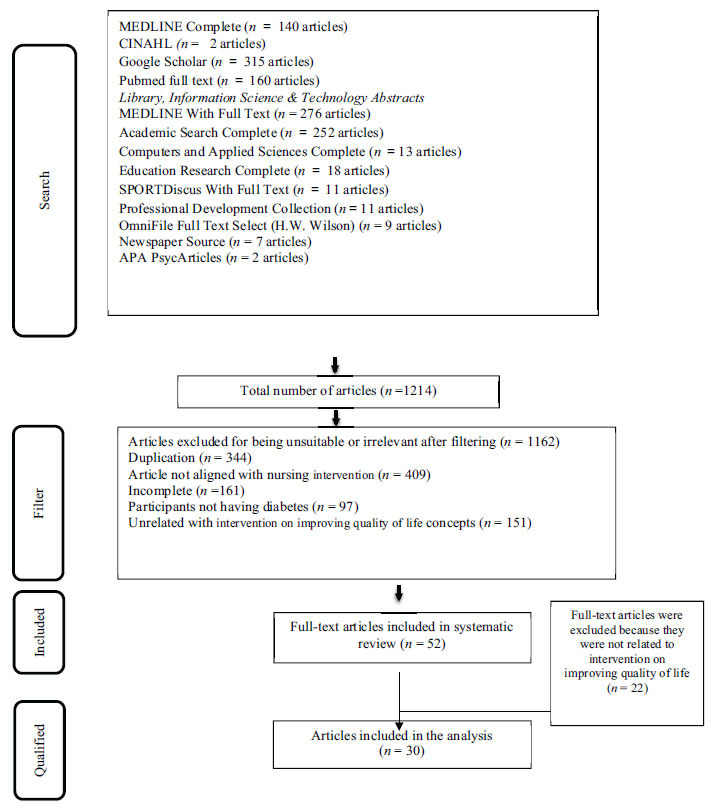
PRISMA flowchart of screening articles on intervention for improving quality of life.

The types of research article designs included in this systematic review were RCT, quasi-experimental, and cross-sectional design. These types of research designs were set to answer the research questions determined from the initial study (Fig. **1**).

### 2.2 Inclusion and Exclusion Criteria

#### 2.2.1 Types of Studies

All intervention studies related to nursing interventions that contribute to improving the QoL of DM patients were included.

#### 2.2.2 Types of Participants/Respondents

The older adult and elderly participants were limited to be included in this review.

#### 2.2.3 Types of Interventions

All types of intervention studies that contributed to improving the QoL of DM patients were included in this review.

#### 2.2.4 Types of Outcome Measurements

The types of outcome measurements were limited to the effect of nursing interventions on improving the QoL of DM patients.

#### 2.2.5 Search Strategy of Literature

This systematic review was conducted by including published research articles between 2017 and 2022 with a set of keywords through several online databases, such as ScienceDirect, Medline, Google Search, and Pro-quest (Table **1**). Articles with accessible pdf full text and containing RCT, quasi-experiment, and cross-sectional design were selected. The discovered articles were then systematically analyzed to meet the defined inclusion criteria. The population characteristics of DM patients who received nursing interventions that contributed to improving their QoL were used.

#### 2.2.6 Method of Assessment and Appraisal of the Articles’ Quality

Articles that met the inclusion criteria were systematically analyzed and their level of evidence was determined and synthesized (Table **2**). Concerning these systematic procedures, the findings of this review can be summarized into nursing interventions on improving the QoL of DM patients, which are beneficial as the fundamentals of implementing nursing care in hospital and community settings.

#### 2.2.7 Data Extraction Methods

Data extraction was performed by reading all of selected the articles’ findings and then summarizing their main contents. The main contents of the articles included the title of the study, authors’ details, study methods, number and characteristics of samples, and the number of intervention and control groups (Table **3**).

## 3 RESULTS

A total of 30 articles defining the types of nursing intervention for improving the QoL of DM patients that met the inclusion and exclusion criteria were searched from various types of study methods, including RCT, quasi-experiment, and cross-sectional design. Those selected articles were then systematically analyzed. Five types of nursing interventions that contributed to improving the QoL of DM patients were concluded as health education (15 articles), exercise (8 articles), WhatsApp/short message service (WA/SMS) gateway (3 articles), blood glucose control (3 articles), and black garlic herbal therapy (1 article). The research locations of the articles were diverse such as the USA, China, Indonesia, India, Saudi Arabia, England, Spain, Singapore, Jordan, Malaysia, Turkey, Romania, Brazil, Pakistan, Thailand, Canada, Kuwait, Netherlands, and Iran.

### 3.1 Health Education

An investigation study [7] described that the QoL of DM patients was improved after the implementation of health education intervention. Health education was provided with an empowerment process model. The intervention group received a 6-week empowerment-based transitional care program, emphasizing personally meaningful goals, facilitated collaborative partnerships, shared decision-making, and an approach through situational reflection. While the control group received health education twice, after discharge from the hospital and during routine visitation assessment-management. Other findings of that study also mentioned the level of empowerment and decreased DM status. Furthermore, another published study [8] highlighted that health education has an effective impact on blood glucose control in the intervention group. During the intervention, blood glucose levels in the intervention group were well maintained, while in the control group, there was no alteration. Furthermore, a study [8] underlined that diabetic foot ulcers (DFUs) are considered a major complication of DM, which strongly impacts patients’ QoL. The aim of the study was to determine the QoL of DM patients (HRQoL) and the management of DFUs. The results showed that HRQoL scores improved in DM patients who received foot care management education. Research on diabetic ulcer patients, using the DFS-SF questionnaire (Diabetic foot scale short form-8), determined that patients with diabetic ulcers had a low means of QoL score on the physical and mental components and men have a significantly higher DSF-SF score than women. Moreover, a study about complementary and alternative medicine (CAM) in DM patients with inclusion criteria of age, gender, family income, occupation, length of suffering, and complications showed that the use of CAM was relatively high so it was concluded that the development of CAM management is necessary [9]. The increasing incidence of DM is a raising concern in the health sectors worldwide, including in Saudi Arabia. In a research study [10], an SF 36 questionnaire was implemented to determine random sugar levels in the community. It underlined that the incidence of DM affects the QoL of adolescents, but the domain of physical function is the most influential aspect impacting social activities. Moreover, an investigation study on a 6-month community-based program improved the HRQoL of mental health and self-management domains and reduced depressive symptoms in adults with type 2 diabetes and the intervention group [11]. In addition, a study on the effect of lifestyle interventions on diabetes medical care costs that investigated two groups of patients highlighted that the group without the lifestyle counseling intervention spent more money on diabetes treatment than the lifestyle modification counseling group. There was a significant difference in hospitalization costs with a significant *p*-value of 0.0038. The difference in the cost of hospitalization in DM patients with surgery due to DM complications was higher in a group without lifestyle counseling intervention (*p*-value 0.0111). Significant improvement was also found in the quality of life domain, which included physical, emotional, and social functioning [5].

### 3.2 Exercise

The findings of the study on the Prolanis exercise intervention on blood sugar levels in DM patients showed a significant result in reducing blood sugar levels (*p*-value: 0.001). Before and after the intervention, the average blood sugar levels were, respectively, 150.31 mg/dl (SD 24,730 mg/dl) and 127.33 mg/dl (SD 14,764 mg/dl). Prolanis exercise has the benefit of preventing obesity by burning calories within the body. During Prolanis activity, blood glucose can be used as energy [12]. Furthermore, a meta-analysis study about yoga intervention revealed that yoga significantly affected the physical function of DM patients (5% significance level). The physical function of the intervention group that was actively implementing yoga was better than the control group that was not actively implementing yoga [13]. Yoga was also found to improve the QoL in physical and social domains [14]. Aerobic physical exercise has a greater impact on blood sugar, HbA1c, and QoL. Aerobic exercise, resistance training, and a combination of both activities have benefits in reducing blood glucose and HbA1c levels, as well as improving the QoL of patients with type 2 diabetes [15]. Another study on aerobic exercise found that the social and environmental domains in the peer group and the environmental domains in the yoga group significantly increased the QoL scores. Moreover, 96% of the intervention group's members reported feeling the program's benefits. The main goal of treating chronic DM is to improve well-being and achieve QoL [16]. The concept related to the QoL of DM patients is well-being, which assesses the positive aspects of an individual’s life, such as positive emotions and life satisfaction. Well-being measurement assesses a positive evaluation of a person's daily life, such as healthier and satisfied feelings or satisfaction with the quality of good relationships, positive emotions, resilience, and realization of personal potential. Yoga practice aims to improve the quality of life by modifying the level of fitness that is safe and suitable for all ages [17]. A research study that combined the Sun and Yang and Kai Mai styles of Tai Chi and Qigong came to the conclusion that Tai Chi can enhance quality of life. It was concluded that Tai Chi might play a significant role in encouraging individuals to become more spirited and physically active. Tai Chi is a gentle activity that has the ability to put people at ease and demonstrates a positive impact of the intervention on the quality of life of DM patients [7]. Moreover, a study conducted on patients with type 2 diabetes showed that yoga contributed to increasing QoL. While study on type 2 DM that focused on metabolic factors and glycemic control after yoga showed significant differences in pain disturbance, Fullerton Advanced Balance scale, upper limb strength, lower limb strength, and QoL scores [18]. Yoga requires less equipment and it is relatively inexpensive. Once trained, the patients can practice at home, thus leading to long-term adherence. Yoga is a relatively low-cost intervention to reduce stress and improve the QoL with type 2 diabetes. By supporting stress resilience, yoga can prevent stress-induced increases in cortisol, thereby controlling the elevation of blood sugar levels [19]. It has been reported that Tai Chi statistically improved QoL as measured by SF-36 in each domain of physical function [20]. Furthermore, aromatherapy is a complementary therapeutic method that uses essential oils for therapy. Aromatherapy in several studies has been shown to have benefits against pain, anxiety, depression, cognitive function, and sleep disorders in the elderly. The study on the effect of aromatherapy and massage on neuropathic pain and QoL of DM patients demonstrated a significantly increased quality of life score in the intervention group in the fourth week of the study [21]. In addition, research on the efficacy of Pilates-based mattress training programs on the parameters of QoL, sleep quality, and satisfaction in type 2 DM patients concluded that Pilates-based mattress training has a significant effect on improving the QoL parameters after a 4-week training program. This study also revealed the significant effect of an exercise intervention on QoL, sleep quality, and life satisfaction [4]. Exercise is part of a planned, structured, and repetitive physical activity of body movement to improve and maintain one or more physical components. Exercise can improve sleep quality and prevent chronic diseases [7].

### 3.3 Reminder WA/SMS Gateway

This current review found 3 articles describing the effect of WhatsApp (WA)/Short Message Services (SMS) gateway on QoL of DM patients. These studies described the uses of diabetes self-management smartphone applications on both iOS and Android smartphones, which aimed to manage diabetes through self-management and glycemic control in DM patients. Smartphone applications and their variation features have shown a significant effect on decreasing HbA1c and blood glucose in DM patients. The results of this systematic review found that the implementation of mobile smartphone applications led to a decrease in HbA1c and fasting blood glucose in patients with DM [3]. Mobile health interventions are evolving research to change behavior among patients with chronic diseases. This study showed that interactive text messaging was a viable and enjoyable intervention among a population of adolescents and young adults with DM. Based on participant feedback in the TEACH intervention group, a significant improvement was reported by comparing enrollment scores with 3-month follow-up scores in patients’ activity (*p*-value = 0.01) and quality of life (Global Mental Health *p*-value = 0.01 and Physical Health Global *p*-value = 0.03) [22]. The study on SMS intervention found that SMS-based self-management support programs contributed to the improvement of glycemic control. The effect of the intervention was also seen in foot care behavior and diabetes support levels. This program showed significantly high acceptance in the intervention group with the majority of participants experiencing a decrease in HbA1c at nine months [23]. This application may be a standalone intervention but can also be used as a part of a more comprehensive program. The possible limitations of this intervention might be considered for large-scale delivery. This study provided evidence of the effectiveness of a newly developed smartphone application designed to trigger diabetes self-management [24]. This smartphone application is easy to use for adults and allows a quick assessment of whether personal habits match the recommended healthy lifestyle in terms of nutrition and physical activity. The broad use of mobile health technologies, such as m-health, is promising in providing high validity in diabetes self-care. M-health is a virtual communication tool that can offer psychological support to encourage and facilitate changes in diabetic self-care. It is versatile and quickly adjusts to varied cultural norms. In comparison to standard care without the use of biofeedback devices or software, an additional motivational interview conducted using m-health in Kuwait improved glycemic control. Since the dates intervention is non-pharmacological, negative side effects are anticipated to be limited [25]. The goal of the trial was to determine whether personalized text message in addition to conventional lifestyle recommendations could slow the progression of prediabetes to type 2 diabetes in two different settings (India and UK). If the results are positive, text messages can provide a low-cost and far-reaching modality in addition to diabetes prevention programs globally. Diabetes prevention using intervention and intensive lifestyle monitoring has achieved a reduction in the cumulative incidence of diabetes by 36% and 65%, respectively [26]. All of the described mHealth interventions were tested in RCTs, and eight out of the thirteen interventions showed clinically and statistically significant results. Five interventions, however, had no results or differences in HbA1c reduction between the intervention and control groups of less than 0.5% (5.5 mmol/mol). These interventions include anything from new developments in glucose monitoring and insulin bolus calculators to health education and lifestyle changes. Its components also include communication with distant clinicians using the telemedicine model, educational information, self-monitoring, and automated messages that provide inspiration, education, and feedback. The mHealth intervention's design should take into account input from patients and doctors on lifestyle or workflow integration, as well as usability and contents [27]. In addition, a study [28] highlighted that participants who received a smartphone-based self-management intervention had improved self-efficacy with a large effect size of 0.98 (*p* 0.001), self-care activities with an effect size of 0.90 (*p* 0.001), health-related quality of life with an effect size of 0.26 (*p* 0.01), and lower glycated hemoglobin (pooled MD=-0.55; p).

### 3.4 Glucose Control

Cardiovascular events are less likely in people with intensive glycemic control than in those who receive standard medication over an extended period of time. The findings demonstrated that better glucose management decreased the frequency of diabetic microvascular problems. Although follow-up of the advantages of rigorous glucose control helped improve QoL, it did not demonstrate a substantial decrease in the incidence of cardiovascular disease and reduced mortality in diabetes patients. Nearly all of the associations between glucose control and major cardiovascular events are explained by the mean of glycated hemoglobin level over the past 3 years [29]. Only 1 patient (2%) showed good QoL with controlled fasting blood glucose levels in the category of hyperglycemia [30]. This is in line with the results of a study describing that most of the patients with controlled fasting blood glucose levels in the hyperglycemia category showed a good QoL. The results of the distribution of respondents in terms of fasting blood glucose levels in the emergency room were in accordance with research conducted previously [31], where uncontrolled glycemic control developed long-term complications and stress and impacted the quality of life among DM patients [32]. Therefore, glycemic control is very important to achieve target blood glucose levels and encourage patients to improve QoL. Considering the importance of QoL in diabetic patients, self-monitoring of blood glucose levels involves self-care behaviors that are assessed from adherence to four dimensions, such as nutrition, activity, medication, and glucose control [33].

### 3.5 Black Garlic Herbal Therapy

Hyperglycemia, a hallmark of type 2 DM, and dyslipidemia are linked to a reduced risk of consequences in diabetic patients. Antioxidants may be helpful in preventing diabetes complications, according to mounting research. According to reports, antioxidant minerals and phytochemicals can help lower blood sugar levels and prevent diabetes-related problems. Garlic has been shown in several trials to have a hypoglycemic impact (Lee *et al.*, 2009). According to a recent study, fermented black garlic products include a high concentration of polysaccharides, phenolic compounds, organic sulfur compounds, proteins, and melanoidins, all of which have numerous health advantages (Zhang *et al.*, 2019). In streptozotocin-induced diabetic rats, consumption of an 80% ethanol extract of garlic decreased serum glucose levels, and injection of the extract delayed the onset of hypoglycemia and structural nephropathy. As it is processed at a controlled temperature and humidity, aged black garlic, which has lately become accessible in the Korean market, is an example of a garlic product that is anticipated to have a strong antioxidant capacity (Jing, 2020). Aged black garlic consumption significantly lowered the homeostasis model assessment for insulin resistance (HOMA-IR) by 11.0% and tended to lower serum glucose levels. In db/db rats, diet for 7 weeks dramatically elevated levels of insulin by 12.1% and lowered serum glucose by 8.7% (Seo *et al.*, 2009). Black garlic also generalizes plasma lipid imbalance and increases fibrinolytic activity (Setiawan *et al.*, 2021). In rats given a high-fat diet, the consumption of black garlic enhanced adiponectin and downregulated PAI-1, thus enhancing insulin resistance (Nurmawati *et al.*, 2021). The capacity of black garlic to neutralize free radicals, such as hydroxyl radicals, 2,2-azino-bis (3-ethylbenzenthiazoline-6-sulfonic) acid (ABTS), and DPPH, was enhanced by L. bulgaricus (Si *et al.*, 2019). This is associated with an improvement in gut microbial composition, which has been shown to decrease blood sugar levels and limit body weight in diabetic patients. By lowering lipid and glucose levels, butyrate synthesis by the gut bacteria may be one of the key mechanisms regulating energy metabolism. The potent antioxidant content is beneficial for protecting the heart against atherosclerosis, where the presence of SAC has the potential to provide anti-inflammatory benefits and inhibit endothelial vasodilation (Setiawan *et al.*, 2021).

## 4 DISCUSSION

The types of nursing interventions that have been proven to be able to improve the QoL of DM patients are discussed in this review. First, exercise is effective in burning and converting sugar into energy and can improve the QoL of DM patients. Subsequently, the WA/SMS gateway is capable of reducing HbA1c and blood glucose as well as behavioral changes among patients with chronic disease. Various nursing interventions are indeed proven to be able to significantly improve QoL and reduce blood glucose levels and anxiety levels in DM patients. DM is viewed as a significant public health issue that negatively impacts sufferers' QoL. The high prevalence has a negative impact on the patient's health and causes a number of issues with low quality of life. From a self-evaluation of the consequences of diabetes care, one of the most significant elements influencing treatment results is the quality of life (QoL). Patients' individual expectations, attitudes, behaviors, and information regarding specific diseases have a significant impact on their quality of life [32]. Diabetes self-management education (DSME) is a crucial program that helps patients maintain regular blood sugar control and has been shown to be a successful strategy. The curriculum for all prepared content includes information on the fundamentals of diabetes, the value of self-management, and self-care. It is conducted over the course of several sessions and includes presentations, discussions, demonstrations, and self-care. The DSME program has a beneficial impact on enhancing type 2 diabetes patients' health status. For diabetic patients to improve QoL, exercise is a powerful and motivating tool. Self-management of diabetes and blood sugar levels was demonstrated by an intervention trial in Thailand [34]. Prior to the intervention, there were no appreciable changes in the participants' age, education, blood sugar monitoring behavior, health examination, knowledge, self-care, stress, or hemoglobin HbA1c (> 0.05) between the two groups' baseline characteristics. However, type 2 DM patients in the intervention group demonstrated significant changes (0.05) in HbA1c serum test, stress levels, and QoL following the intervention. While the control group's results remained unaltered, there was no statistically significant difference (>0.05) between the two groups. The results of the study showed that DSME improved the QoL of adult female patients with type 2 diabetes by lowering blood glucose levels, reducing tension, and lowering anxiety. Therefore, patients with diabetes may benefit from this intervention [32].

Diabetes is a difficult and complex condition that people must manage on a daily basis. A foundation is provided by DSMES to help DM patients navigate their daily self-care with confidence and respectable results. It addresses the combination of clinical, educational, psychosocial, and behavioral aspects of care needed for daily self-management [35]. Encouraging patients to use DSMES helps to implement informed decision-making, self-management behaviors, problem-solving, and active collaboration with healthcare providers to improve critical outcomes, health status, and QoL. The goal of DSMES is to provide DM patients with the knowledge, skills, and confidence to accept responsibility for self-management of type 2 DM. DSMES has various benefits, including clinical, psychosocial, and behavioral outcomes, in type 2 DM patients, such as improving QoL, healthy life planning, and patient involvement in regular physical activity [35, 36].

A technology-based strategy to improve patients’ behavior in DM treatment provides controlled treatment services to patients at home using mobile smartphone technology. An SMS-based reminder system was tested in the Netherlands to determine the effect of an SMS reminder on patient adherence to oral antidiabetic drugs using real-time medication monitoring. The study proved that SMS reminders were effective in increasing treatment adherence of type 2 DM patients, which aimed to prevent patients from dropping out from the treatments. The reminder system application is open source and proposed as a non-profit reminder that provides important information quickly, inexpensively, and accurately to the target group's cellphone numbers. The reminder system produces monitoring report output, visiting reports, and reminder reports that help in monitoring outpatient DM patients [37].

According to an updated current investigation, non-pharmacological therapy has a positive effect in reducing blood sugar levels, fasting blood sugar levels, HbA1c, and excessive use of diabetes drugs in type 2 DM patients. The results showed that exercise therapy is a promising intervention for type 2 DM management [38]. The prognosis of the DM affects patients’ QoL, where most of them have been suffering for years. It causes the patients to feel restless and hopeless in treating the disease, especially for patients who experience the DM complications that can contribute to a negative impact on their QoL. In addition, due to low knowledge about the DM prognosis, patients mostly do not understand the need for treatments. Therefore, they feel hopeless about what to do and it affects their QoL [15].

Type 2 DM self-management is not only limited to education, but with simultaneous and regular exercise, type 2 DM patients will achieve a higher level of satisfaction of QoL compared to patients with less exercise management [39]. The higher incidence of type 2 DM can be associated with adverse lifestyles, such as lack of physical activity and obesity. A previous study showed that exercise has effective results for type 2 DM management. However, the optimal intensity of exercise to prevent type 2 DM progression remains to be investigated. A prior RCT investigation found that moderate-intensity exercise was effective in improving blood glucose tolerance. Regular exercise can improve insulin sensitivity and help to control blood glucose levels and lose weight. Traditional Chinese “Qigong” and Indian exercises, including Taichi, Baduanjin, and Yoga, which combine body regulation and breathing with unifying body movements, have been frequently used by type 2 DM patients [38]. Based on the proceeding study, exercise can increase the QoL of DM patients with peripheral neuropathy. Diabetic peripheral neuropathy can cause leg pain, ulcers, and body amputation, which seriously affect the QoL. Exercising with family can encourage DM patients to exercise, improve glycemic control, and foster a positive attitude toward life. Moreover, it can make patients feel better in terms of physical and psychological manners and improve their social relationships [7].

A prior prospective diabetes study revealed that intensive glucose control can significantly reduce risk to the cardiovascular system [40]. Self-monitoring glucose control (SMGC) is the standard of care in DM patients with continuous glucose monitoring. The SMGC monitoring system has more advantages for DM patients in making clinical decisions and alerting them of integrated hypoglycemic events every 2 weeks. The integration of the results of the SMGC system provides a more complete description of glucose control throughout the week. Therefore, it is useful to reduce complications in DM patients [41].

Moreover, the complementary and alternative medicine studies reported the use of black garlic (BG) *(Allium sativum)* as one of the effective complementary interventions to prevent diabetes complications, since it has been tested and used to prevent hypoglycemia in diabetic patients. BG contains high antioxidants when exposed to humidity and fermentation. The antioxidant activity of BG is influenced by the way it is processed. Allicin is an unstable compound in onions that is converted into a stable compound during the aging process, so it can produce a stronger antioxidant content that is capable of being used as treatment management for type 2 DM patients [42]. Recent studies have reported that bioactive compounds in the BG have various biological activities and pharmacological properties that show their effectiveness in preventing various types of diseases. Most of the benefits of BG can be attributed to its antioxidant, anti-inflammatory, anti-obesity, anti-cancer, anti-allergic, and hypolipidemic properties. With the increasing prevalence of chronic diseases, such as diabetes, health has become a top priority for research, with the aim of finding new foods and tactics to address the health burden on patients [43]. Onions have been used throughout the world as traditional medicine to treat several disorders, such as rheumatism, diabetes, and other diseases [44].

## CONCLUSION

This recent systematic review concluded that nursing intervention methods, such as exercise, WA/SMS gateway, health education, BG herbal therapy, and glucose control, are proven to improve the QoL of DM patients. It is also evidenced that these nursing interventions are very popular in Asian, American, European, and African countries. The application of these types of interventions is relatively easy, low cost, and has been supported by adequate training facilities and instructors. Health education is the most frequently implemented nursing intervention. Moreover, it has been reported that the personal approach has become a trend. However, further research needs to be conducted in the future.

## Figures and Tables

**Table 1:** Databases search strategy.

**Search Strategy on the Online Databases**
Keywords and steps of articles search through online databases 1. Intervention AND Nursing Interventions AND Diabetes Mellitus AND Quality of Life 2. Education AND Interventions AND Quality of life AND Diabetes Mellitus 3. Blood sugar level AND Interventions AND Quality Of life AND Diabetes Mellitus 4. Exercise AND Interventions AND Quality of Life AND Diabetes Mellitus 5. Black Garlic AND Interventions AND Quality of Life AND Diabetes Mellitus Level 3. #1 AND #2

**Table 2:** Data extraction and assessment of articles’ quality.

No	Author (Year)	Method	SMS Gateway	Education	Glucose Control	Exercise	Black Garlic	Level of Evidence	Summary Quality of Article
1	Sylvia (2021) [12]	QE	-	-	-	✓	-	2	Good
2	Bota (2021) [45]	SR	-	-	✓	-	-	3	Fair
3	Gomez (2021) [14]	RCT	-	-	-	✓	-	3	Good
4	Fajriah (2020) [15]	SR	-	-	-	✓	-	1	Good
5	Yao (2020) [8]	RCT	-	✓		-	-	1	Good
6	Rosyid (2020) [46]	RCT	-	-	✓	-	-	3	Fair
7	Sesaria (2020) [47]	CS	✓		-	-	-	2	Good
8	Khunkaew (2019) [48]	CS	-	✓	-	-	-	3	Fair
9	Alrub (2019) [49]	CS	-	✓	-	-	-	2	Good
10	Joeliantina (2019) [9]	RCT	-	✓	-	-	-	2	Good
11	Ibrahim (2019) [10]	CS	-	✓	-	-	-	3	Fair
12	Almaramhi (2018) [50]	RCT	-		✓	-	-	3	Fair
13	Nagaraja Y. (2018) [17]	QE	-	-	-	✓	-	3	Fair
14	Thomson (2018) [26]	RCT	✓		-		-	2	Good
15	Cunningham (2018) [51]	RCT	-	✓	-		-	2	Good
16	Sharma (2018) [52]	ES	-		-	✓	-	2	Good
17	Borji (2017) [53]	QE	-	✓	-		-	3	Fair
18	Sreedevi (2017) [16]	RCT	-		-	✓	-	2	Good
19	Iqbal (2017) [54]	CS	-	✓	-		-	2	Good
20	Metin (2017) [21]	RCT	-		-	✓	-	2	Good
21	Teston (2017) [55]	RCT	-	✓	-		-	2	Good
22	Roifah (2016) [56]	CS	-	✓	-		-	3	Fair
23	Cai (2016) [7, 56]	SR	-		-	✓	-	1	Good
24	Kueh (2016) [38]	CS	-	✓	-	-	-	3	Fair
25	Al-Aboudi (2015) [57]	CS	-	✓	-	-	-	3	Fair
26	Kumar (2021) [32]	QE	-	✓	-	-	-	1	Good
27	Hust (2020) [34]	QE	-	✓	-	-	-	2	Good
28	Lubis (2016) [58]	QE	✓		-	-	-	2	Good
29	Power (2020) [59]	CS	-	✓	-	-	-	1	Good
30	Si (2019) [60]	RCT	-	-	-	-	✓	1	Good

**Annotation:** RCT: Randomized Controlled Trials; SR: Systematic Review; MA: Meta-Analysisl; CS: Cross-Sectional; QE: Quasi-Experimental; ES: Experimental Study.

**Table 3:** Summary of articles’ assessment.

**No.**	**Author**	**Title**	**Intervention**	**Significance**
1.	Sylvia (2021) [12]	The Effect of Prolanis Exercise Activities on Decreasing Blood Sugar Levels in Diabetes Mellitus Patients (2021)	Along with physical activity and sports, the Prolanis group was also given education, with a focus on DM nutrition diet, amount, and type of food, as well as regularity in medication and blood sugar measurement.	The findings revealed that there was a difference in blood sugar levels between the intervention and control groups with a *p*-value of 0.008 and that the Prolanic exercise activities were applied to treat type 2 diabetes patients at the Padangmatingi Public Health Center in Padangsidimpuan.
2	Bota (2021) [45]	Glucose Monitoring for Type 1 Diabetes Mellitus in Junior Rhythmic Gymnastics – A Case Report	To overcome the dread of hypoglycemia, which keeps diabetics from exercising, glucose monitoring is created by giving them physical activity activities.	The gymnast participated in training sessions as long as her glycemic levels permitted her to engage in physical activity. In order to distinguish between the glucose profiles on training days and non-training days, it was important from the perspective of diabetes management to highlight the distribution of glycaemic values (in range, above, and below the goal zone) in bimonthly and daily reports.
3	Gomez (2021) [14]	Effect of High-Intensity Interval Training on Quality of Life, Sleep Quality, Exercise Motivation, and Enjoyment in Sedentary People with Type 1 Diabetes Mellitus	Since hypoglycemia prevention and time constraints are the main obstacles for this population to exercising and improving physical conditioning, high-intensity interval training (HIIT) is an effective and safe practice method.	Since no cases of severe hypoglycemia were reported, we suggest that the 6-week HIIT program used in the current study is both safe and a successful method for enhancing HRQoL, sleep quality, exercise motivation, and enjoyment, all of which are crucial psychological well-being factors in T1DM patients.
4	Fajriah (2020) [15]	The Effects from Physical Exercise on the Blood Glucose Levels, HbA1c and Quality of Life of Type 2 Diabetes Mellitus Patients: A Systematic Review	The benefits of resistance training, aerobic exercise, and a combination of both in HbA1c, blood sugar levels, and quality of life in T2DM patients are explored. Several research and recommendations for the management of T2DM promote physical activity.	The clinical situation and the patient's specific physical fitness must be taken into consideration when choosing the type and intensity of exercise for the therapy of T2DM. To evaluate the combined benefits of resistance training and aerobic exercise on glucose, HbA1c, and the quality of life of T2DM adjusted for different age categories, more research is required.
5	Yao (2020) [8]	Effect of Information-Motivation-Behavioral Model Based on Protection Motivation Theory on the Psychological Resilience and Quality of Life of Patients with Type 2 DM (2020)	It is crucial to combine the IMB intervention with the protection motive theory to help type 2 DM patients become more psychologically resilient, improve their quality of life, and lower their blood glucose levels.	Prior to the study, there was not a measurable difference between the treatment and control groups (*p*> 0.05). Following the intervention, our treatment group's blood glucose level and depression score were substantially lower compared to those of the experimental group (*p*<0.05), and their levels of psychological resilience and quality of life were significantly greater. Following the intervention, the treatment group's blood glucose level and depressed mood scale were reduced (*p*<0.05), and their psychological resilience and quality of life considerably improved (*p*<0.05). The baseline group's blood sugar levels, depression, psychological toughness, and quality of life did not differ significantly before and after the treatment (*p*> 0.05).
6	Rosyid (2020) [46]	Fasting Blood Glucose Levels Associated with Quality of Life in Diabetic Foot Ulcer Patients	Evaluating the relationship between fasting blood glucose (GDP) levels and quality of life in people with diabetic foot ulcers.	In individuals with diabetic foot ulcers, GDP levels were substantially correlated with quality of life (*p*=0.04). Blood sugar levels are strongly correlated with patients' quality of life when they have diabetic foot ulcers.
7	Sesaria (2020) [47]	Mobile Smartphone Intervention For Managing Glycaemia Control in Patients with Diabetes Mellitus: A Systematic Review	Effectiveness of a mobile smartphone application for regulating blood sugar levels in people with diabetes.	According to the findings of this literature analysis, people with diabetes mellitus who used mobile phones had lower HbA1c and fasting blood glucose levels. The results of this study support the effectiveness of the mobile diabetes intervention in maintaining glycaemia control in diabetes mellitus patients.
8	Khunkaew (2019) [48]	Health-Related Quality of Life and self-care Management Among People With Diabetic Foot Ulcers in Northern Thailand	To check about how patients with DFUs manage their foot care and their HRQoL. In the self-care management of people with DFUs, foot care is crucial.	The HRQOL and self-care behaviours of people with DFUs in Northern Thailand have never been studied before. The findings show that improving HRQoL requires tailored, targeted foot care instruction that includes self-care management techniques.
9	Alrub (2019) [49]	Factors Associated with Health-Related Quality of Life among Jordanian Patients with Diabetic Foot Ulcer	Diabetic Foot Scale-Short Form (DFS-SF) and Short Form-8, two self-administered questionnaires, were used to measure health-related quality of life (SF-8).	Low mean DFS-SF scores and low mean scores on the physical and mental component summary scales were observed in patients with diabetic foot ulcers (PCS8 and MCS8). Males had considerably higher DFS-SF scores than females, indicating a superior quality of life in terms of health (*p* =value 0.03).
10	Joeliantina (2019) [9]	A Literature Review of Complementary and Alternative Medicine Used Among Diabetes Mellitus Patients	Along with conventional treatment, some diabetic patients also use complementary and alternative medicine (CAM) to preserve their health and regulate their blood sugar.	Depending on the patients who use it, CAM might be classified as supplementary medicine, alternative medicine, or integrative medicine. Chronic illness patients frequently use CAM.
11	Ibrahim (2019) [10]	Diabetes Prevalence and Quality of Life of Female Nursing Students	The King Saud bin Abdulaziz University for Health Sciences in Riyadh evaluated the impact of diabetes mellitus on the quality of life of female nursing students (KSAU-HS).	This analysis found that the measured quality of life areas for people with diabetes mellitus was negatively impacted by constraints on activities, emotional health issues, and social activities.
12	Almaramhi (2018) [50]	The Correlation of Fasting Blood Glucose Levels with the Severity of Diabetic Foot Ulcers and the Outcome of Treatment Strategies	The relationship between fasting blood sugar levels, DFU grades, and the effectiveness of proposed treatment methods.	144 (57.1%) of the 252 patients had fasting blood glucose levels below 220 mg, and 14 (5.6%) had readings between 80 and 100 mg. The majority of patients (131; 51.9%) had grades 4 and 5 DFUs at presentation. Only 83 (32.9%) of the 123 amputations done improved, while 154 (61.1%) did not (*p*-value 0.00).
13	Nagaraja Y (2018) [17]	Discovering the Benefits of Yoga and Improving Quality of Life	Advantages of consistent yoga practice and enhancement of life quality. There is proof that yoga practice increases both physical and mental capabilities.	Yoga is a type of mind-body exercise that combines physical exertion with a conscious interior emphasis on awareness of the self, the breath, and energy. Yoga is quite helpful for its advantages and enhancing quality of life.
14	Thomson (2018) [26]	Protocol for a Clinical Trial of Text Messaging in Addition to Standard Care *versus* Standard Care Alone in Prevention of Type 2 Diabetes through Lifestyle Modification in India and the UK	The effectiveness and user satisfaction of text messaging for patients in the UK and India who have pre-diabetes (HbA1c 6.0% to 6.4%; 42-47 mmol/mol) were explored. The adoption of a healthy lifestyle through food and exercise adjustment can aid in the prevention of type 2 diabetes.	In order to slow the progression from prediabetes to type 2 diabetes in the two separate countries, the study evaluated the effectiveness of personalized SMS messages in addition to conventional lifestyle recommendations.
15	Cunningham (2018) [51]	The Effect of Diabetes Self-Management Education on Hba1c and Quality of Life in African-Americans: A Systematic Review and Meta-analysis	Results from a meta-analysis revealed that DSME had no discernible impact on African Americans' HbA1c levels. QoL did improve, and it is a crucial DSME endpoint to track in upcoming studies. Understanding the impact of DSME on HbA1c in this population requires more research.	Participants in the intervention and those receiving normal care did not differ significantly in HbA1c weighted mean: 0.08% [0.40-0.^23^]; 2 = 84.79 (*p*< .001), I 2 = 92%, (n = 1630). In four out of five studies that measured QoL, participants in interventions showed a significant improvement.
16	Sharma (2018) [52]	Efficacy Of Pilates-based Mat Exercise on Quality of Life, Quality of Sleep, and Satisfaction with Life in Type 2 Diabetes Mellitus	Give physical therapy (a Pilates-based mat workout) a try to improve your quality of life, your ability to sleep, and your overall sense of pleasure with life.	The findings indicated a highly substantial impact of exercise on both life quality and sleep quality, as well as a considerable impact on overall life satisfaction. Pilates-based mat exercises had a positive impact on all metrics, including individuals with type 2 diabetes mellitus' quality of life, sleep quality, and life satisfaction.
17	Borji (2017) [53]	The Impact of Orem's self-care Model on the Quality of Life in Patients with Type II Diabetes	The impact of Orem's self-care strategy on patients with type II diabetes in Ilam, Iran, considering quality of life (QoL).	The results revealed that the experimental group's mean and standard deviation for quality of life before and after the intervention were, respectively, 47.1 9.21 and 67.91 12.87, which was statistically significant (*p*< 0.05).
18	Sreedevi (2017) [16]	The Effect of Yoga and Peer Support Interventions on the Quality of Life of Women with Diabetes: Results of a Randomized Controlled Trial	The impact of yoga and peer support, two low-cost therapies, on the level of well-being (QoL) of women with diabetes type 2.	Most study participants (96%) thought peer support and yoga interventions were helpful. Paired t-tests showed significant changes in the environment and social domains in the friendship group and the context of various interventions in the yoga group. However, this disintegrated between other group comparisons, which might have been caused by the study's short period of three months and poor diabetes control (hemoglobin A1c ranged from 9.4 to 9.6).
19	Iqbal (2017) [54]	Profile and Predictors of Health-Related Quality of Life among Type II Diabetes Mellitus Patients in Quetta City, Pakistan	In the study, a model for HRQoL in patients with T2DM is presented, with medication adherence acting as a predictor of HRQoL. Healthcare practitioners should pay close attention to how their patients take their medications and make an attempt to explain to them the advantages of doing so.	In the current study, patients' HRQoL was subpar, with mean scores of 0.48 and 0.36. In a cross-tabulation analysis, significant relationships were reported between HRQoL and age, disease duration, number of prescription medications, medication adherence, and treatment satisfaction (*p* 0.05). The important variables were added to the model after it passed the very relevant Omnibus Test of Model Coefficient (Chi-square = 12.983, *p* = 0.030, df = 4) and showed a considerable goodness of fit.
20	Metin (2017) [21]	Aromatherapy Massage for Neuropathic Pain and Quality of Life in Diabetic Patients	The impact of treatment on patients with painful diabetic neuropathy's quality of life (QoL) and degree of neuropathic pain.	In order to alleviate neuropathic pain and enhance QoL in patients with acute neuropathy, aromatherapy massage is a quick and efficient nonpharmacological nursing intervention.
21	Teston (2017) [55]	Effect of the Consultation of Nursing on Knowledge, Quality of Life, Attitude towards Disease, and self-care among Persons with Diabetes	To examine how self-care-based nursing consultation affects patients with type 2 diabetes mellitus' knowledge of and attitudes toward their disease, as well as their quality of life and adherence to self-care rituals (DM).	The IG revealed a significant shift in education about diabetes (*p*<0.001), the impact of the condition on life satisfaction (*p* = 0.002), approach toward the condition (*p* = 0.024), and self-care activity compliance (*p*<0.001). The nursing consultation on encouraging self-care has a beneficial influence on knowledge, attitude, and commitment to personality activities, but it also has an increased impact on quality of life.
22	Roifah (2016) [56]	Analysis of the Relationship of Long Suffering with Diabetes Mellitus with Quality of Life of People with Diabetes Mellitus	The goal of this study was to determine the association between chronic diabetes mellitus and the level of life of those who have it.	The *p*-value<0.05 indicates that there is a connection between the duration of suffering and the standard of living of DM patients at the external medical centre of RSUD Prof. Dr. Wahidin Sudiro Husodo.
23	Cai (2016) [56]	Effect of Exercise on the Quality of Life in Type 2 Diabetes Mellitus: A Systematic Review	The quality of life of a type 2 diabetic patient before and after physical activity is evaluated. Exercise is one of the crucial diabetic therapies.	We divided the exercise into four modes: aerobic, resistance, a combination of aerobic and resistance, and yoga. Aerobic exercise showed a significant effect between groups. Resistance and combined exercise showed mixed results. Yoga also showed good intervention effects on quality of life.
24	Kueh (2016) [39]	The Effect of Diabetes Knowledge and Attitudes on Self-Management and Quality of Life among People with Type 2 Diabetes	The Summary of Diabetes Self-Care Activities (SDSCA) scale, the Diabetes Knowledge Scale (DKN), the Diabetes Integration Scale-19 (ATT19), and the Diabetes Quality of Life (DQoL) scale, which measure, respectively, diabetes knowledge, attitudes, self-management, and QoL is presented.	A topic of behaviors and personality in areas of monitoring blood glucose levels and foot care was diabetes knowledge. The influence of QoL was significantly predicted by attitudes. Diet was a strong predictor and impacted the QoL, and personality in the context of glucose control was a major correlate of influence on QoL. Aspects of self-management, such as exercise and foot care, were highly predictive of satisfaction and the influence of QoL, respectively.
25	Al-Aboudi (2015) [57]	A Cross-Sectional Assessment of Health-related Quality of Life among Type 2 Diabetes Patients in Riyadh, Saudi Arabia	To outline the profile of type 2 diabetes patients' care quality of life in Riyadh, Saudi Arabia. This study has shown that the wellness quality of life for Saudi diabetic patients is poor. When preparing comprehensive patient treatment strategies, healthcare providers must take this into account.	Wellness life satisfaction scores did not significantly correlate with age groups, the period of diabetes, marital status, degree of education, or kind of therapy.
26	Kumar (2021) [32]	Effects of Diabetes Self-management Education Program on Lowering Blood Glucose Level, Stress, and Quality of Life among Females with Type 2 Diabetes Mellitus in Thailand	To determine the impact of diabetic identity education (DSME) on improving quality of life (QoL), stress reduction, and blood glucose control in Thai women with type 2 diabetes.	Before the intervention began, the baseline characteristics of the two groups were comparable, and no significant differences were found in the categories of age, degree, blood glucose measuring habit, clinical examination, knowledge, self-care, stress, or hemoglobin HbA1c (>0.05).
27	Hust (2020) [34]	Impact of Diabetes Self-management, Diabetes Management Self-efficacy, and Diabetes Knowledge on Glycemic Control in People with Type 2 Diabetes (T2D): A Multicenter Study in Thailand	The antecedent constructs of diabetes treatment self-efficacy and diabetic knowledge have been found to be closely related to effective diabetic self-management.	In our analysis, 52.4% of the patients had blood sugar that was out of control (HbA1c > 7%). The bivariate analysis revealed that all three psychometric measures (DK, DMSE, and DSM) were related to blood glucose control. Blood glucose control has been found to be substantially correlated with diabetes management self-efficacy in the Thai Type 2 diabetic population.
28	Lubis (2016) [58]	SMS-based Reminder System Design to Improve Medication Adherence of Diabetes Mellitus Patients	Sending SMS reminders to patients as part of the delivery of health services is one technology-based approach to enhancing drug adherence.	Since the result of the remember system report suggests that 81.3% of participants attended the clinic after getting a reminder SMS, using a notification proposed system can be advised as a technique to increase adherence to treatment of patients with diabetes mellitus.
29	Power (2020) [59]	Diabetes Self-management Education and Support in Adults with Type 2 Diabetes: A Consensus Report of the American Diabetes Association, the Association of Diabetes Care and Education Specialists, the Academy of Nutrition and Dietetics, the American Academy of Family Physicians, the American Academy of PAs, the American Association of Nurse Practitioners, and the American Pharmacists Association	Diabetes Consciousness Education and Guidance (DSMES) focuses on the full variety of clinical, intellectual, sociological, and psychosocial aspects of care necessary for everyday self-management and lays the groundwork for assisting all diabetics in navigating their basic self with optimism and improved outcomes.	The clinical, psychosocial, and behavioral facets of diabetes are all improved by DSMES. The foundation receives ongoing support from DSMES to encourage the accomplishment of individual goals and impact the best results. DSMES has been shown to have numerous advantages, however, only a small percentage of persons with diabetes are actually referred for and receive treatment.
30	Si (2019) [60]	*Lactobacillus bulgaricus* Improves the Antioxidant Capacity of Black Garlic in the Prevention of Gestational Diabetes Mellitus: A Randomized Control Trial.	Black garlic's antioxidant activity may be enhanced by *Lactobacillus bulgaricus* to help prevent diabetes during pregnancy (GDM). *L. bulgaricus* was used in the preparation of black garlic.	There were 40 weeks of treatment. Blood glucose levels were found following an oral glucose tolerance (OGTT) and at 1, 2, and 3 hours after fasting (FBG, 1hBG, and 2hBG). Measurements of plasma malondialdehyde (MDA), superoxide dismutase (SOD), glutathione peroxidase (GSH-PX), and total antioxidant capacity (T-AOC) in GDM patients were used to assess the antioxidant efficacy of black garlic. Two independent samples of the *t*-test were used to compare the two groups. *L. bulgaricus* decreased the incidence of fetal problems as well as the rate of FBG, 1hBG, and 2hBG.

## Data Availability

The data and supportive information are available within the article.

## LIST OF ABBREVIATIONS

RCT: Randomized Controlled Trial
DM: Diabetes Mellitus
HRQoL: Health-Related Quality of Life
QoL: Quality of Life
CAM: Complementary and Alternative Medicine

## SUPPLEMENTARY MATERIAL:

PRISMA checklist is available as supplementary material on the publisher’s website along with the published article. Supplementary material is available on the publisher’s website along with the published article.
