# Supplementary material for: Types of Nursing Intervention on Improving Quality of Life among Patients with Diabetes Mellitus: A Systematic Review
Source: Curr Diabetes Rev. 2024 Jan 30;20(3):e290823220467. doi: 10.2174/1573399820666230829103016 (PMC10909825; doi:10.2174/1573399820666230829103016)
Supplement: Supplementary file 1 — PRISMA checklist is available as supplementary material on the publisher’s website along with the published article. Supplementary material is available on the publisher’s website along with the published article. [file CDR-20-E290823220467_SD1.pdf]

Supplementary Material

Types of Nursing Intervention on Improving Quality of Life among Patients with Diabetes Mellitus: A Systematic Review

Agung Samsu Alam[1], Amin Samiasih[1,*], Mohammad Fatkhul Mubin[1], Satriya Pranata[1] and Reina Dhamanik[1]

[1]*Department of Nursing, Faculty of Nursing and Health Sciences, Universitas Muhammadiyah Semarang, Semarang City, Central Java, Indonesia*

Artikel 1

Section and Topic	Item #	Checklist item	Location where item is reported
**TITLE**			
Title	1	Identify the report as a systematic review.	The Effect of Prolanis Exercise Activities on Decreasing Blood Sugar Levels in Diabetes Mellitus Patients (2021)
**ABSTRACT**			
Abstract	2	See the PRISMA 2020 for Abstracts checklist.	The authors has well described it
**INTRODUCTION**			
Rationale	3	Describe the rationale for the review in the context of existing knowledge.	Yes
Objectives	4	Provide an explicit statement of the objective(s) or question(s) the review addresses.	Yes
**METHODS**			
Eligibility criteria	5	Specify the inclusion and exclusion criteria for the review and how studies were grouped for the syntheses.	No
Information sources	6	Specify all databases, registers, websites, organisations, reference lists and other sources searched or consulted to identify studies. Specify the date when each source was last searched or consulted.	Yes
Search strategy	7	Present the full search strategies for all databases, registers and websites, including any filters and limits used.	Yes
Selection process	8	Specify the methods used to decide whether a study met the inclusion criteria of the review, including how many reviewers screened each record and each report retrieved, whether they worked independently, and if applicable, details of automation tools used in the process.	No
Data collection process	9	Specify the methods used to collect data from reports, including how many reviewers collected data from each report, whether they worked independently, any processes for obtaining or confirming data from study investigators, and if applicable, details of automation tools used in the process.	No
Data items	10a	List and define all outcomes for which data were sought. Specify whether all results that were compatible with each outcome domain in each study were sought (e.g. for all measures, time points, analyses), and if not, the methods used to decide which results to collect.	Yes
	10b	List and define all other variables for which data were sought (e.g. participant and intervention characteristics, funding sources). Describe any assumptions made about any missing or unclear information.	Yes
Study risk of bias assessment	11	Specify the methods used to assess risk of bias in the included studies, including details of the tool(s) used, how many reviewers assessed each study and whether they worked independently, and if applicable, details of automation tools used in the process.	No
Effect	12	Specify for each outcome the effect measure(s) (e.g. risk ratio, mean	Yes

Section and Topic	Item #	Checklist item	Location where item is reported
measures		difference) used in the synthesis or presentation of results.	
Synthesis methods	13a	Describe the processes used to decide which studies were eligible for each synthesis (e.g. tabulating the study intervention characteristics and comparing against the planned groups for each synthesis (item #5)).	Yes
	13b	Describe any methods required to prepare the data for presentation or synthesis, such as handling of missing summary statistics, or data conversions.	Yes
	13c	Describe any methods used to tabulate or visually display results of individual studies and syntheses.	Yes
	13d	Describe any methods used to synthesize results and provide a rationale for the choice(s). If meta-analysis was performed, describe the model(s), method(s) to identify the presence and extent of statistical heterogeneity, and software package(s) used.	Yes
	13e	Describe any methods used to explore possible causes of heterogeneity among study results (e.g. subgroup analysis, meta-regression).	Yes
	13f	Describe any sensitivity analyses conducted to assess robustness of the synthesized results.	Yes
Reporting bias assessment	14	Describe any methods used to assess risk of bias due to missing results in a synthesis (arising from reporting biases).	No
Certainty assessment	15	Describe any methods used to assess certainty (or confidence) in the body of evidence for an outcome.	Yes
**RESULTS**			
Study selection	16a	Describe the results of the search and selection process, from the number of records identified in the search to the number of studies included in the review, ideally using a flow diagram.	Yes
	16b	Cite studies that might appear to meet the inclusion criteria, but which were excluded, and explain why they were excluded.	Yes
Study characteristics	17	Cite each included study and present its characteristics.	Yes
Risk of bias in studies	18	Present assessments of risk of bias for each included study.	No
Results of individual studies	19	For all outcomes, present, for each study: (a) summary statistics for each group (where appropriate) and (b) an effect estimate and its precision (e.g. confidence/credible interval), ideally using structured tables or plots.	Yes
Results of syntheses	20a	For each synthesis, briefly summarise the characteristics and risk of bias among contributing studies.	Yes
	20b	Present results of all statistical syntheses conducted. If meta-analysis was done, present for each the summary estimate and its precision (e.g. confidence/credible interval) and measures of statistical heterogeneity. If comparing groups, describe the direction of the effect.	Yes
	20c	Present results of all investigations of possible causes of heterogeneity among study results.	No
	20d	Present results of all sensitivity analyses conducted to assess the robustness of the synthesized results.	Yes
Reporting biases	21	Present assessments of risk of bias due to missing results (arising from reporting biases) for each synthesis assessed.	No
Certainty of evidence	22	Present assessments of certainty (or confidence) in the body of evidence for each outcome assessed.	Yes
**DISCUSSION**			
Discussion	23a	Provide a general interpretation of the results in the context of other evidence.	Yes
	23b	Discuss any limitations of the evidence included in the review.	Yes

Section and Topic	Item #	Checklist item	Location where item is reported
	23c	Discuss any limitations of the review processes used.	Yes
	23d	Discuss implications of the results for practice, policy, and future research.	Yes
**OTHER INFORMATION**			
Registration and protocol	24a	Provide registration information for the review, including register name and registration number, or state that the review was not registered.	Yes
	24b	Indicate where the review protocol can be accessed, or state that a protocol was not prepared.	Yes
	24c	Describe and explain any amendments to information provided at registration or in the protocol.	No
Support	25	Describe sources of financial or non-financial support for the review, and the role of the funders or sponsors in the review.	No
Competing interests	26	Declare any competing interests of review authors.	Yes
Availability of data, code and other materials	27	Report which of the following are publicly available and where they can be found: template data collection forms; data extracted from included studies; data used for all analyses; analytic code; any other materials used in the review.	Yes

From: Page MJ, McKenzie JE, Bossuyt PM, Boutron I, Hoffmann TC, Mulrow CD, et al. The PRISMA 2020 statement: an updated guideline for reporting systematic reviews. BMJ 2021;372:n71. doi: 10.1136/bmj.n71

For more information, visit: http://www.prisma-statement.org/

Artikel 2

Section and Topic	Item #	Checklist item	Location where item is reported
**TITLE**			
Title	1	Identify the report as a systematic review.	Glucose Monitoring For Type 1 Diabetes Mellitus In Junior Rhythmic Gymnastics
**ABSTRACT**			
Abstract	2	See the PRISMA 2020 for Abstracts checklist.	The auktors has well described it
**INTRODUCTION**			
Rationale	3	Describe the rationale for the review in the context of existing knowledge.	Yes
Objectives	4	Provide an explicit statement of the objective(s) or question(s) the review addresses.	Yes
**METHODS**			
Eligibility criteria	5	Specify the inclusion and exclusion criteria for the review and how studies were grouped for the syntheses.	Yes
Information sources	6	Specify all databases, registers, websites, organisations, reference lists and other sources searched or consulted to identify studies. Specify the date when each source was last searched or consulted.	Yes
Search strategy	7	Present the full search strategies for all databases, registers and websites, including any filters and limits used.	Yes
Selection process	8	Specify the methods used to decide whether a study met the inclusion criteria of the review, including how many reviewers screened each record and each report retrieved, whether they worked independently, and if applicable, details of automation tools used in the process.	No
Data collection process	9	Specify the methods used to collect data from reports, including how many reviewers collected data from each report, whether they worked independently, any processes for obtaining or confirming data from study investigators, and if applicable, details of automation tools used in the process.	No

Section and Topic	Item #	Checklist item	Location where item is reported
Data items	10a	List and define all outcomes for which data were sought. Specify whether all results that were compatible with each outcome domain in each study were sought (e.g. for all measures, time points, analyses), and if not, the methods used to decide which results to collect.	Yes
	10b	List and define all other variables for which data were sought (e.g. participant and intervention characteristics, funding sources). Describe any assumptions made about any missing or unclear information.	Yes
Study risk of bias assessment	11	Specify the methods used to assess risk of bias in the included studies, including details of the tool(s) used, how many reviewers assessed each study and whether they worked independently, and if applicable, details of automation tools used in the process.	No
Effect measures	12	Specify for each outcome the effect measure(s) (e.g. risk ratio, mean difference) used in the synthesis or presentation of results.	No
Synthesis methods	13a	Describe the processes used to decide which studies were eligible for each synthesis (e.g. tabulating the study intervention characteristics and comparing against the planned groups for each synthesis (item #5)).	Yes
	13b	Describe any methods required to prepare the data for presentation or synthesis, such as handling of missing summary statistics, or data conversions.	No
	13c	Describe any methods used to tabulate or visually display results of individual studies and syntheses.	Yes
	13d	Describe any methods used to synthesize results and provide a rationale for the choice(s). If meta-analysis was performed, describe the model(s), method(s) to identify the presence and extent of statistical heterogeneity, and software package(s) used.	No
	13e	Describe any methods used to explore possible causes of heterogeneity among study results (e.g. subgroup analysis, meta-regression).	No
	13f	Describe any sensitivity analyses conducted to assess robustness of the synthesized results.	No
Reporting bias assessment	14	Describe any methods used to assess risk of bias due to missing results in a synthesis (arising from reporting biases).	No
Certainty assessment	15	Describe any methods used to assess certainty (or confidence) in the body of evidence for an outcome.	Yes
**RESULTS**			
Study selection	16a	Describe the results of the search and selection process, from the number of records identified in the search to the number of studies included in the review, ideally using a flow diagram.	Yes
	16b	Cite studies that might appear to meet the inclusion criteria, but which were excluded, and explain why they were excluded.	No
Study characteristics	17	Cite each included study and present its characteristics.	Yes
Risk of bias in studies	18	Present assessments of risk of bias for each included study.	No
Results of individual studies	19	For all outcomes, present, for each study: (a) summary statistics for each group (where appropriate) and (b) an effect estimate and its precision (e.g. confidence/credible interval), ideally using structured tables or plots.	No
Results of syntheses	20a	For each synthesis, briefly summarise the characteristics and risk of bias among contributing studies.	No
	20b	Present results of all statistical syntheses conducted. If meta-analysis was done, present for each the summary estimate and its precision (e.g. confidence/credible interval) and measures of statistical heterogeneity. If comparing groups, describe the direction of the effect.	No
	20c	Present results of all investigations of possible causes of heterogeneity	No

Section and Topic	Item #	Checklist item	Location where item is reported
		among study results.	
	20d	Present results of all sensitivity analyses conducted to assess the robustness of the synthesized results.	Yes
Reporting biases	21	Present assessments of risk of bias due to missing results (arising from reporting biases) for each synthesis assessed.	No
Certainty of evidence	22	Present assessments of certainty (or confidence) in the body of evidence for each outcome assessed.	Yes
**DISCUSSION**			
Discussion	23a	Provide a general interpretation of the results in the context of other evidence.	Yes
	23b	Discuss any limitations of the evidence included in the review.	Yes
	23c	Discuss any limitations of the review processes used.	No
	23d	Discuss implications of the results for practice, policy, and future research.	Yes
**OTHER INFORMATION**			
Registration and protocol	24a	Provide registration information for the review, including register name and registration number, or state that the review was not registered.	Yes
	24b	Indicate where the review protocol can be accessed, or state that a protocol was not prepared.	Not described
	24c	Describe and explain any amendments to information provided at registration or in the protocol.	Not described
Support	25	Describe sources of financial or non-financial support for the review, and the role of the funders or sponsors in the review.	No
Competing interests	26	Declare any competing interests of review authors.	No
Availability of data, code and other materials	27	Report which of the following are publicly available and where they can be found: template data collection forms; data extracted from included studies; data used for all analyses; analytic code; any other materials used in the review.	No

From: Page MJ, McKenzie JE, Bossuyt PM, Boutron I, Hoffmann TC, Mulrow CD, et al. The PRISMA 2020 statement: an updated guideline for reporting systematic reviews. BMJ 2021;372:n71. doi: 10.1136/bmj.n71
For more information, visit: http://www.prisma-statement.org/

Artikel 3

Section and Topic	Item #	Checklist item	Location where item is reported
**TITLE**			
Title	1	Identify the report as a systematic review.	Effect of High-Intensity Interval Training on Quality of Life, Sleep Quality, Exercise Motivation and Enjoyment in Sedentary People with Type 1 Diabetes Mellitus
**ABSTRACT**			
Abstract	2	See the PRISMA 2020 for Abstracts checklist.	The authors has well described it
**INTRODUCTION**			
Rationale	3	Describe the rationale for the review in the context of existing knowledge.	Yes
Objectives	4	Provide an explicit statement of the objective(s) or question(s) the review addresses.	Yes
**METHODS**			
Eligibility criteria	5	Specify the inclusion and exclusion criteria for the review and how studies were grouped for the syntheses.	Yes
Information	6	Specify all databases, registers, websites, organisations, reference lists and other sources searched or consulted to identify studies.	Yes

Section and Topic	Item #	Checklist item	Location where item is reported
sources		Specify the date when each source was last searched or consulted.	
Search strategy	7	Present the full search strategies for all databases, registers and websites, including any filters and limits used.	Yes
Selection process	8	Specify the methods used to decide whether a study met the inclusion criteria of the review, including how many reviewers screened each record and each report retrieved, whether they worked independently, and if applicable, details of automation tools used in the process.	Yes
Data collection process	9	Specify the methods used to collect data from reports, including how many reviewers collected data from each report, whether they worked independently, any processes for obtaining or confirming data from study investigators, and if applicable, details of automation tools used in the process.	Yes
Data items	10a	List and define all outcomes for which data were sought. Specify whether all results that were compatible with each outcome domain in each study were sought (e.g. for all measures, time points, analyses), and if not, the methods used to decide which results to collect.	Yes
	10b	List and define all other variables for which data were sought (e.g. participant and intervention characteristics, funding sources). Describe any assumptions made about any missing or unclear information.	Yes
Study risk of bias assessment	11	Specify the methods used to assess risk of bias in the included studies, including details of the tool(s) used, how many reviewers assessed each study and whether they worked independently, and if applicable, details of automation tools used in the process.	Yes
Effect measures	12	Specify for each outcome the effect measure(s) (e.g. risk ratio, mean difference) used in the synthesis or presentation of results.	Yes
Synthesis methods	13a	Describe the processes used to decide which studies were eligible for each synthesis (e.g. tabulating the study intervention characteristics and comparing against the planned groups for each synthesis (item #5)).	Yes
	13b	Describe any methods required to prepare the data for presentation or synthesis, such as handling of missing summary statistics, or data conversions.	Yes
	13c	Describe any methods used to tabulate or visually display results of individual studies and syntheses.	Yes
	13d	Describe any methods used to synthesize results and provide a rationale for the choice(s). If meta-analysis was performed, describe the model(s), method(s) to identify the presence and extent of statistical heterogeneity, and software package(s) used.	Yes
	13e	Describe any methods used to explore possible causes of heterogeneity among study results (e.g. subgroup analysis, meta-regression).	Yes
	13f	Describe any sensitivity analyses conducted to assess robustness of the synthesized results.	Yes
Reporting bias assessment	14	Describe any methods used to assess risk of bias due to missing results in a synthesis (arising from reporting biases).	Yes
Certainty assessment	15	Describe any methods used to assess certainty (or confidence) in the body of evidence for an outcome.	Yes
**RESULTS**			
Study selection	16a	Describe the results of the search and selection process, from the number of records identified in the search to the number of studies included in the review, ideally using a flow diagram.	Yes

Section and Topic	Item #	Checklist item	Location where item is reported
	16b	Cite studies that might appear to meet the inclusion criteria, but which were excluded, and explain why they were excluded.	Yes
Study characteristics	17	Cite each included study and present its characteristics.	Yes
Risk of bias in studies	18	Present assessments of risk of bias for each included study.	No
Results of individual studies	19	For all outcomes, present, for each study: (a) summary statistics for each group (where appropriate) and (b) an effect estimate and its precision (e.g. confidence/credible interval), ideally using structured tables or plots.	Yes
Results of syntheses	20a	For each synthesis, briefly summarise the characteristics and risk of bias among contributing studies.	Yes
	20b	Present results of all statistical syntheses conducted. If meta-analysis was done, present for each the summary estimate and its precision (e.g. confidence/credible interval) and measures of statistical heterogeneity. If comparing groups, describe the direction of the effect.	Yes
	20c	Present results of all investigations of possible causes of heterogeneity among study results.	No
	20d	Present results of all sensitivity analyses conducted to assess the robustness of the synthesized results.	Yes
Reporting biases	21	Present assessments of risk of bias due to missing results (arising from reporting biases) for each synthesis assessed.	No
Certainty of evidence	22	Present assessments of certainty (or confidence) in the body of evidence for each outcome assessed.	Yes
**DISCUSSION**			
Discussion	23a	Provide a general interpretation of the results in the context of other evidence.	Yes
	23b	Discuss any limitations of the evidence included in the review.	Yes
	23c	Discuss any limitations of the review processes used.	Yes
	23d	Discuss implications of the results for practice, policy, and future research.	
**OTHER INFORMATION**			
Registration and protocol	24a	Provide registration information for the review, including register name and registration number, or state that the review was not registered.	Yes
	24b	Indicate where the review protocol can be accessed, or state that a protocol was not prepared.	Yes
	24c	Describe and explain any amendments to information provided at registration or in the protocol.	Yes
Support	25	Describe sources of financial or non-financial support for the review, and the role of the funders or sponsors in the review.	No
Competing interests	26	Declare any competing interests of review authors.	Yes
Availability of data, code and other materials	27	Report which of the following are publicly available and where they can be found: template data collection forms; data extracted from included studies; data used for all analyses; analytic code; any other materials used in the review.	Yes

From: Page MJ, McKenzie JE, Bossuyt PM, Boutron I, Hoffmann TC, Mulrow CD, et al. The PRISMA 2020 statement: an updated guideline for reporting systematic reviews. BMJ 2021;372:n71. doi: 10.1136/bmj.n71

For more information, visit: http://www.prisma-statement.org/

Artikel 4

Section and Topic	Item #	Checklist item	Location where item is reported
**TITLE**			
Title	1	Identify the report as a systematic review.	The Effects from Physical Exercise on the Blood Glucose Levels, HbA1c and Quality of Life of Type 2 Diabetes Mellitus Patients
**ABSTRACT**			
Abstract	2	See the PRISMA 2020 for Abstracts checklist.	The authors has well described it
**INTRODUCTION**			
Rationale	3	Describe the rationale for the review in the context of existing knowledge.	Yes
Objectives	4	Provide an explicit statement of the objective(s) or question(s) the review addresses.	Yes
**METHODS**			
Eligibility criteria	5	Specify the inclusion and exclusion criteria for the review and how studies were grouped for the syntheses.	Yes
Information sources	6	Specify all databases, registers, websites, organisations, reference lists and other sources searched or consulted to identify studies. Specify the date when each source was last searched or consulted.	Yes
Search strategy	7	Present the full search strategies for all databases, registers and websites, including any filters and limits used.	Yes
Selection process	8	Specify the methods used to decide whether a study met the inclusion criteria of the review, including how many reviewers screened each record and each report retrieved, whether they worked independently, and if applicable, details of automation tools used in the process.	Yes
Data collection process	9	Specify the methods used to collect data from reports, including how many reviewers collected data from each report, whether they worked independently, any processes for obtaining or confirming data from study investigators, and if applicable, details of automation tools used in the process.	Yes
Data items	10a	List and define all outcomes for which data were sought. Specify whether all results that were compatible with each outcome domain in each study were sought (e.g. for all measures, time points, analyses), and if not, the methods used to decide which results to collect.	Yes
	10b	List and define all other variables for which data were sought (e.g. participant and intervention characteristics, funding sources). Describe any assumptions made about any missing or unclear information.	Yes
Study risk of bias assessment	11	Specify the methods used to assess risk of bias in the included studies, including details of the tool(s) used, how many reviewers assessed each study and whether they worked independently, and if applicable, details of automation tools used in the process.	No
Effect measures	12	Specify for each outcome the effect measure(s) (e.g. risk ratio, mean difference) used in the synthesis or presentation of results.	Yes
Synthesis methods	13a	Describe the processes used to decide which studies were eligible for each synthesis (e.g. tabulating the study intervention characteristics and comparing against the planned groups for each synthesis (item #5)).	Yes
	13b	Describe any methods required to prepare the data for presentation or synthesis, such as handling of missing summary statistics, or data conversions.	Yes
	13c	Describe any methods used to tabulate or visually display results of individual studies and syntheses.	Yes
	13d	Describe any methods used to synthesize results and provide a rationale for the choice(s). If meta-analysis was performed, describe the	Yes

Section and Topic	Item #	Checklist item	Location where item is reported
		model(s), method(s) to identify the presence and extent of statistical heterogeneity, and software package(s) used.	
	13e	Describe any methods used to explore possible causes of heterogeneity among study results (e.g. subgroup analysis, meta-regression).	Yes
	13f	Describe any sensitivity analyses conducted to assess robustness of the synthesized results.	Yes
Reporting bias assessment	14	Describe any methods used to assess risk of bias due to missing results in a synthesis (arising from reporting biases).	Yes
Certainty assessment	15	Describe any methods used to assess certainty (or confidence) in the body of evidence for an outcome.	Yes
**RESULTS**			
Study selection	16a	Describe the results of the search and selection process, from the number of records identified in the search to the number of studies included in the review, ideally using a flow diagram.	Yes
	16b	Cite studies that might appear to meet the inclusion criteria, but which were excluded, and explain why they were excluded.	Yes
Study characteristics	17	Cite each included study and present its characteristics.	Yes
Risk of bias in studies	18	Present assessments of risk of bias for each included study.	No
Results of individual studies	19	For all outcomes, present, for each study: (a) summary statistics for each group (where appropriate) and (b) an effect estimate and its precision (e.g. confidence/credible interval), ideally using structured tables or plots.	Yes
Results of syntheses	20a	For each synthesis, briefly summarise the characteristics and risk of bias among contributing studies.	Yes
	20b	Present results of all statistical syntheses conducted. If meta-analysis was done, present for each the summary estimate and its precision (e.g. confidence/credible interval) and measures of statistical heterogeneity. If comparing groups, describe the direction of the effect.	Yes
	20c	Present results of all investigations of possible causes of heterogeneity among study results.	Yes
	20d	Present results of all sensitivity analyses conducted to assess the robustness of the synthesized results.	Yes
Reporting biases	21	Present assessments of risk of bias due to missing results (arising from reporting biases) for each synthesis assessed.	No
Certainty of evidence	22	Present assessments of certainty (or confidence) in the body of evidence for each outcome assessed.	Yes
**DISCUSSION**			
Discussion	23a	Provide a general interpretation of the results in the context of other evidence.	Yes
	23b	Discuss any limitations of the evidence included in the review.	Yes
	23c	Discuss any limitations of the review processes used.	Yes
	23d	Discuss implications of the results for practice, policy, and future research.	No
**OTHER INFORMATION**			
Registration and protocol	24a	Provide registration information for the review, including register name and registration number, or state that the review was not registered.	Yes
	24b	Indicate where the review protocol can be accessed, or state that a protocol was not prepared.	Yes
	24c	Describe and explain any amendments to information provided at	Yes

Section and Topic	Item #	Checklist item	Location where item is reported
		registration or in the protocol.	
Support	25	Describe sources of financial or non-financial support for the review, and the role of the funders or sponsors in the review.	No
Competing interests	26	Declare any competing interests of review authors.	No
Availability of data, code and other materials	27	Report which of the following are publicly available and where they can be found: template data collection forms; data extracted from included studies; data used for all analyses; analytic code; any other materials used in the review.	Yes

From: Page MJ, McKenzie JE, Bossuyt PM, Boutron I, Hoffmann TC, Mulrow CD, et al. The PRISMA 2020 statement: an updated guideline for reporting systematic reviews. BMJ 2021;372:n71. doi: 10.1136/bmj.n71

For more information, visit: http://www.prisma-statement.org/

Artikel 5

Section and Topic	Item #	Checklist item	Location where item is reported
**TITLE**			
Title	1	Identify the report as a systematic review.	Effect of Information-Motivation-Behavioral Model Based on Protection Motivation Theory on the Psychological Resilience and Quality of Life of Patients with Type 2 DM
**ABSTRACT**			
Abstract	2	See the PRISMA 2020 for Abstracts checklist.	The authors has well described it
**INTRODUCTION**			
Rationale	3	Describe the rationale for the review in the context of existing knowledge.	Yes
Objectives	4	Provide an explicit statement of the objective(s) or question(s) the review addresses.	Yes
**METHODS**			
Eligibility criteria	5	Specify the inclusion and exclusion criteria for the review and how studies were grouped for the syntheses.	Yes
Information sources	6	Specify all databases, registers, websites, organisations, reference lists and other sources searched or consulted to identify studies. Specify the date when each source was last searched or consulted.	Yes
Search strategy	7	Present the full search strategies for all databases, registers and websites, including any filters and limits used.	Yes
Selection process	8	Specify the methods used to decide whether a study met the inclusion criteria of the review, including how many reviewers screened each record and each report retrieved, whether they worked independently, and if applicable, details of automation tools used in the process.	Yes
Data collection process	9	Specify the methods used to collect data from reports, including how many reviewers collected data from each report, whether they worked independently, any processes for obtaining or confirming data from study investigators, and if applicable, details of automation tools used in the process.	Yes
Data items	10a	List and define all outcomes for which data were sought. Specify whether all results that were compatible with each outcome domain in each study were sought (e.g. for all measures, time points, analyses), and if not, the methods used to decide which results to collect.	Yes
	10b	List and define all other variables for which data were sought (e.g. participant and intervention characteristics, funding sources). Describe any assumptions made about any missing or unclear information.	Yes

Section and Topic	Item #	Checklist item	Location where item is reported
Study risk of bias assessment	11	Specify the methods used to assess risk of bias in the included studies, including details of the tool(s) used, how many reviewers assessed each study and whether they worked independently, and if applicable, details of automation tools used in the process.	No
Effect measures	12	Specify for each outcome the effect measure(s) (e.g. risk ratio, mean difference) used in the synthesis or presentation of results.	Yes
Synthesis methods	13a	Describe the processes used to decide which studies were eligible for each synthesis (e.g. tabulating the study intervention characteristics and comparing against the planned groups for each synthesis (item #5)).	Yes
	13b	Describe any methods required to prepare the data for presentation or synthesis, such as handling of missing summary statistics, or data conversions.	Yes
	13c	Describe any methods used to tabulate or visually display results of individual studies and syntheses.	Yes
	13d	Describe any methods used to synthesize results and provide a rationale for the choice(s). If meta-analysis was performed, describe the model(s), method(s) to identify the presence and extent of statistical heterogeneity, and software package(s) used.	Yes
	13e	Describe any methods used to explore possible causes of heterogeneity among study results (e.g. subgroup analysis, meta-regression).	Yes
	13f	Describe any sensitivity analyses conducted to assess robustness of the synthesized results.	Yes
Reporting bias assessment	14	Describe any methods used to assess risk of bias due to missing results in a synthesis (arising from reporting biases).	No
Certainty assessment	15	Describe any methods used to assess certainty (or confidence) in the body of evidence for an outcome.	Yes
**RESULTS**			
Study selection	16a	Describe the results of the search and selection process, from the number of records identified in the search to the number of studies included in the review, ideally using a flow diagram.	Yes
	16b	Cite studies that might appear to meet the inclusion criteria, but which were excluded, and explain why they were excluded.	Yes
Study characteristics	17	Cite each included study and present its characteristics.	Yes
Risk of bias in studies	18	Present assessments of risk of bias for each included study.	No
Results of individual studies	19	For all outcomes, present, for each study: (a) summary statistics for each group (where appropriate) and (b) an effect estimate and its precision (e.g. confidence/credible interval), ideally using structured tables or plots.	Yes
Results of syntheses	20a	For each synthesis, briefly summarise the characteristics and risk of bias among contributing studies.	Yes
	20b	Present results of all statistical syntheses conducted. If meta-analysis was done, present for each the summary estimate and its precision (e.g. confidence/credible interval) and measures of statistical heterogeneity. If comparing groups, describe the direction of the effect.	Yes
	20c	Present results of all investigations of possible causes of heter-	Yes

Section and Topic	Item #	Checklist item	Location where item is reported
		ogeneity among study results.	
	20d	Present results of all sensitivity analyses conducted to assess the robustness of the synthesized results.	Yes
Reporting biases	21	Present assessments of risk of bias due to missing results (arising from reporting biases) for each synthesis assessed.	No
Certainty of evidence	22	Present assessments of certainty (or confidence) in the body of evidence for each outcome assessed.	Yes
**DISCUSSION**			
Discussion	23a	Provide a general interpretation of the results in the context of other evidence.	Yes
	23b	Discuss any limitations of the evidence included in the review.	Yes
	23c	Discuss any limitations of the review processes used.	Yes
	23d	Discuss implications of the results for practice, policy, and future research.	Yes
**OTHER INFORMATION**			
Registration and protocol	24a	Provide registration information for the review, including register name and registration number, or state that the review was not registered.	Yes
	24b	Indicate where the review protocol can be accessed, or state that a protocol was not prepared.	Yes
	24c	Describe and explain any amendments to information provided at registration or in the protocol.	Yes
Support	25	Describe sources of financial or non-financial support for the review, and the role of the funders or sponsors in the review.	No
Competing interests	26	Declare any competing interests of review authors.	Yes
Availability of data, code and other materials	27	Report which of the following are publicly available and where they can be found: template data collection forms; data extracted from included studies; data used for all analyses; analytic code; any other materials used in the review.	Yes

From: Page MJ, McKenzie JE, Bossuyt PM, Boutron I, Hoffmann TC, Mulrow CD, et al. The PRISMA 2020 statement: an updated guideline for reporting systematic reviews. BMJ 2021;372:n71. doi: 10.1136/bmj.n71
For more information, visit: http://www.prisma-statement.org/

Artikel 6

Section and Topic	Item #	Checklist item	Location where item is reported
**TITLE**			
Title	1	Identify the report as a systematic review.	Fasting Blood Glucose Levels And Associated With Quality Of Life In Diabetic Foot Ulcer Patients
**ABSTRACT**			
Abstract	2	See the PRISMA 2020 for Abstracts checklist.	The authors has well describe it
**INTRODUCTION**			
Rationale	3	Describe the rationale for the review in the context of existing knowledge.	Yes
Objectives	4	Provide an explicit statement of the objective(s) or question(s) the review addresses.	Yes
**METHODS**			
Eligibility criteria	5	Specify the inclusion and exclusion criteria for the review and how studies were grouped for the syntheses.	Yes

Section and Topic	Item #	Checklist item	Location where item is reported
Information sources	6	Specify all databases, registers, websites, organisations, reference lists and other sources searched or consulted to identify studies. Specify the date when each source was last searched or consulted.	Yes
Search strategy	7	Present the full search strategies for all databases, registers and websites, including any filters and limits used.	Yes
Selection process	8	Specify the methods used to decide whether a study met the inclusion criteria of the review, including how many reviewers screened each record and each report retrieved, whether they worked independently, and if applicable, details of automation tools used in the process.	Yes
Data collection process	9	Specify the methods used to collect data from reports, including how many reviewers collected data from each report, whether they worked independently, any processes for obtaining or confirming data from study investigators, and if applicable, details of automation tools used in the process.	Yes
Data items	10a	List and define all outcomes for which data were sought. Specify whether all results that were compatible with each outcome domain in each study were sought (e.g. for all measures, time points, analyses), and if not, the methods used to decide which results to collect.	Yes
	10b	List and define all other variables for which data were sought (e.g. participant and intervention characteristics, funding sources). Describe any assumptions made about any missing or unclear information.	Yes
Study risk of bias assessment	11	Specify the methods used to assess risk of bias in the included studies, including details of the tool(s) used, how many reviewers assessed each study and whether they worked independently, and if applicable, details of automation tools used in the process.	No
Effect measures	12	Specify for each outcome the effect measure(s) (e.g. risk ratio, mean difference) used in the synthesis or presentation of results.	Yes
Synthesis methods	13a	Describe the processes used to decide which studies were eligible for each synthesis (e.g. tabulating the study intervention characteristics and comparing against the planned groups for each synthesis (item #5)).	Yes
	13b	Describe any methods required to prepare the data for presentation or synthesis, such as handling of missing summary statistics, or data conversions.	Yes
	13c	Describe any methods used to tabulate or visually display results of individual studies and syntheses.	Yes
	13d	Describe any methods used to synthesize results and provide a rationale for the choice(s). If meta-analysis was performed, describe the model(s), method(s) to identify the presence and extent of statistical heterogeneity, and software package(s) used.	Yes
	13e	Describe any methods used to explore possible causes of heterogeneity among study results (e.g. subgroup analysis, meta-regression).	Yes
	13f	Describe any sensitivity analyses conducted to assess robustness of the synthesized results.	Yes
Reporting bias assessment	14	Describe any methods used to assess risk of bias due to missing results in a synthesis (arising from reporting biases).	No
Certainty assessment	15	Describe any methods used to assess certainty (or confidence) in the body of evidence for an outcome.	Yes
**RESULTS**			
Study selection	16a	Describe the results of the search and selection process, from the number of records identified in the search to the number of studies included in the review, ideally using a flow diagram.	Yes
	16b	Cite studies that might appear to meet the inclusion criteria, but which were excluded, and explain why they were excluded.	Yes
Study characteris-	17	Cite each included study and present its characteristics.	Yes

Section and Topic	Item #	Checklist item	Location where item is reported
tics			
Risk of bias in studies	18	Present assessments of risk of bias for each included study.	No
Results of individual studies	19	For all outcomes, present, for each study: (a) summary statistics for each group (where appropriate) and (b) an effect estimate and its precision (e.g. confidence/credible interval), ideally using structured tables or plots.	Yes
Results of syntheses	20a	For each synthesis, briefly summarise the characteristics and risk of bias among contributing studies.	No
	20b	Present results of all statistical syntheses conducted. If meta-analysis was done, present for each the summary estimate and its precision (e.g. confidence/credible interval) and measures of statistical heterogeneity. If comparing groups, describe the direction of the effect.	Yes
	20c	Present results of all investigations of possible causes of heterogeneity among study results.	Yes
	20d	Present results of all sensitivity analyses conducted to assess the robustness of the synthesized results.	Yes
Reporting biases	21	Present assessments of risk of bias due to missing results (arising from reporting biases) for each synthesis assessed.	No
Certainty of evidence	22	Present assessments of certainty (or confidence) in the body of evidence for each outcome assessed.	Yes
**DISCUSSION**			
Discussion	23a	Provide a general interpretation of the results in the context of other evidence.	Yes
	23b	Discuss any limitations of the evidence included in the review.	Yes
	23c	Discuss any limitations of the review processes used.	Yes
	23d	Discuss implications of the results for practice, policy, and future research.	No
**OTHER INFORMATION**			
Registration and protocol	24a	Provide registration information for the review, including register name and registration number, or state that the review was not registered.	Yes
	24b	Indicate where the review protocol can be accessed, or state that a protocol was not prepared.	No
	24c	Describe and explain any amendments to information provided at registration or in the protocol.	No
Support	25	Describe sources of financial or non-financial support for the review, and the role of the funders or sponsors in the review.	No
Competing interests	26	Declare any competing interests of review authors.	No
Availability of data, code and other materials	27	Report which of the following are publicly available and where they can be found: template data collection forms; data extracted from included studies; data used for all analyses; analytic code; any other materials used in the review.	No

From: Page MJ, McKenzie JE, Bossuyt PM, Boutron I, Hoffmann TC, Mulrow CD, et al. The PRISMA 2020 statement: an updated guideline for reporting systematic reviews. BMJ 2021;372:n71. doi: 10.1136/bmj.n71

For more information, visit: http://www.prisma-statement.org/

Artikel 7.

Section and Topic	Item #	Checklist item	Location where item is reported
**TITLE**			
Title	1	Identify the report as a systematic review.	Mobile Smartphone Intervention For Managing Glycaemia Control In The Patients With Diabe-

Section and Topic	Item #	Checklist item	Location where item is reported
			tes Mellitus: A Systematic Review
**ABSTRACT**			
Abstract	2	See the PRISMA 2020 for Abstracts checklist.	The authors has well described it
**INTRODUCTION**			
Rationale	3	Describe the rationale for the review in the context of existing knowledge.	Yes
Objectives	4	Provide an explicit statement of the objective(s) or question(s) the review addresses.	Yes
**METHODS**			
Eligibility criteria	5	Specify the inclusion and exclusion criteria for the review and how studies were grouped for the syntheses.	Yes
Information sources	6	Specify all databases, registers, websites, organisations, reference lists and other sources searched or consulted to identify studies. Specify the date when each source was last searched or consulted.	Yes
Search strategy	7	Present the full search strategies for all databases, registers and websites, including any filters and limits used.	Yes
Selection process	8	Specify the methods used to decide whether a study met the inclusion criteria of the review, including how many reviewers screened each record and each report retrieved, whether they worked independently, and if applicable, details of automation tools used in the process.	Yes
Data collection process	9	Specify the methods used to collect data from reports, including how many reviewers collected data from each report, whether they worked independently, any processes for obtaining or confirming data from study investigators, and if applicable, details of automation tools used in the process.	Yes
Data items	10a	List and define all outcomes for which data were sought. Specify whether all results that were compatible with each outcome domain in each study were sought (e.g. for all measures, time points, analyses), and if not, the methods used to decide which results to collect.	Yes
	10b	List and define all other variables for which data were sought (e.g. participant and intervention characteristics, funding sources). Describe any assumptions made about any missing or unclear information.	Yes
Study risk of bias assessment	11	Specify the methods used to assess risk of bias in the included studies, including details of the tool(s) used, how many reviewers assessed each study and whether they worked independently, and if applicable, details of automation tools used in the process.	No
Effect measures	12	Specify for each outcome the effect measure(s) (e.g. risk ratio, mean difference) used in the synthesis or presentation of results.	Yes
Synthesis methods	13a	Describe the processes used to decide which studies were eligible for each synthesis (e.g. tabulating the study intervention characteristics and comparing against the planned groups for each synthesis (item #5)).	Yes
	13b	Describe any methods required to prepare the data for presentation or synthesis, such as handling of missing summary statistics, or data conversions.	Yes
	13c	Describe any methods used to tabulate or visually display results of individual studies and syntheses.	Yes
	13d	Describe any methods used to synthesize results and provide a rationale for the choice(s). If meta-analysis was performed, describe the model(s), method(s) to identify the presence and extent of statistical heterogeneity, and software package(s) used.	Yes

Section and Topic	Item #	Checklist item	Location where item is reported
	13e	Describe any methods used to explore possible causes of heterogeneity among study results (e.g. subgroup analysis, meta-regression).	No
	13f	Describe any sensitivity analyses conducted to assess robustness of the synthesized results.	Yes
Reporting bias assessment	14	Describe any methods used to assess risk of bias due to missing results in a synthesis (arising from reporting biases).	No
Certainty assessment	15	Describe any methods used to assess certainty (or confidence) in the body of evidence for an outcome.	Yes
**RESULTS**			
Study selection	16a	Describe the results of the search and selection process, from the number of records identified in the search to the number of studies included in the review, ideally using a flow diagram.	Yes
	16b	Cite studies that might appear to meet the inclusion criteria, but which were excluded, and explain why they were excluded.	Yes
Study characteristics	17	Cite each included study and present its characteristics.	Yes
Risk of bias in studies	18	Present assessments of risk of bias for each included study.	No
Results of individual studies	19	For all outcomes, present, for each study: (a) summary statistics for each group (where appropriate) and (b) an effect estimate and its precision (e.g. confidence/credible interval), ideally using structured tables or plots.	Yes
Results of syntheses	20a	For each synthesis, briefly summarise the characteristics and risk of bias among contributing studies.	Yes
	20b	Present results of all statistical syntheses conducted. If meta-analysis was done, present for each the summary estimate and its precision (e.g. confidence/credible interval) and measures of statistical heterogeneity. If comparing groups, describe the direction of the effect.	Yes
	20c	Present results of all investigations of possible causes of heterogeneity among study results.	No
	20d	Present results of all sensitivity analyses conducted to assess the robustness of the synthesized results.	Yes
Reporting biases	21	Present assessments of risk of bias due to missing results (arising from reporting biases) for each synthesis assessed.	No
Certainty of evidence	22	Present assessments of certainty (or confidence) in the body of evidence for each outcome assessed.	Yes
**DISCUSSION**			
Discussion	23a	Provide a general interpretation of the results in the context of other evidence.	Yes
	23b	Discuss any limitations of the evidence included in the review.	Yes
	23c	Discuss any limitations of the review processes used.	Yes
	23d	Discuss implications of the results for practice, policy, and future research.	No
**OTHER INFORMATION**			
Registration and protocol	24a	Provide registration information for the review, including register name and registration number, or state that the review was not registered.	Yes
	24b	Indicate where the review protocol can be accessed, or state that a protocol was not prepared.	Yes
	24c	Describe and explain any amendments to information provided at registration or in the protocol.	No
Support	25	Describe sources of financial or non-financial support for the re-	No

Section and Topic	Item #	Checklist item	Location where item is reported
		view, and the role of the funders or sponsors in the review.	
Competing interests	26	Declare any competing interests of review authors.	Yes
Availability of data, code and other materials	27	Report which of the following are publicly available and where they can be found: template data collection forms; data extracted from included studies; data used for all analyses; analytic code; any other materials used in the review.	Yes

From: Page MJ, McKenzie JE, Bossuyt PM, Boutron I, Hoffmann TC, Mulrow CD, et al. The PRISMA 2020 statement: an updated guideline for reporting systematic reviews. BMJ 2021;372:n71. doi: 10.1136/bmj.n71
For more information, visit: http://www.prisma-statement.org/

Artikel 8.

Section and Topic	Item #	Checklist item	Location where item is reported
**TITLE**			
Title	1	Identify the report as a systematic review.	Health-Related Quality of Life and Self-Care Management Among People With Diabetic Foot Ulcers in Northern Thailand
**ABSTRACT**			
Abstract	2	See the PRISMA 2020 for Abstracts checklist.	The authors has well described it
**INTRODUCTION**			
Rationale	3	Describe the rationale for the review in the context of existing knowledge.	Yes
Objectives	4	Provide an explicit statement of the objective(s) or question(s) the review addresses.	Yes
**METHODS**			
Eligibility criteria	5	Specify the inclusion and exclusion criteria for the review and how studies were grouped for the syntheses.	Yes
Information sources	6	Specify all databases, registers, websites, organisations, reference lists and other sources searched or consulted to identify studies. Specify the date when each source was last searched or consulted.	Yes
Search strategy	7	Present the full search strategies for all databases, registers and websites, including any filters and limits used.	Yes
Selection process	8	Specify the methods used to decide whether a study met the inclusion criteria of the review, including how many reviewers screened each record and each report retrieved, whether they worked independently, and if applicable, details of automation tools used in the process.	Yes
Data collection process	9	Specify the methods used to collect data from reports, including how many reviewers collected data from each report, whether they worked independently, any processes for obtaining or confirming data from study investigators, and if applicable, details of automation tools used in the process.	Yes
Data items	10a	List and define all outcomes for which data were sought. Specify whether all results that were compatible with each outcome domain in each study were sought (e.g. for all measures, time points, analyses), and if not, the methods used to decide which results to collect.	Yes
	10b	List and define all other variables for which data were sought (e.g. participant and intervention characteristics, funding sources). Describe any assumptions made about any missing or unclear information.	No
Study risk of bias assessment	11	Specify the methods used to assess risk of bias in the included studies, including details of the tool(s) used, how many reviewers assessed each study and whether they worked independently, and if	No

Section and Topic	Item #	Checklist item	Location where item is reported
		applicable, details of automation tools used in the process.	
Effect measures	12	Specify for each outcome the effect measure(s) (e.g. risk ratio, mean difference) used in the synthesis or presentation of results.	Yes
Synthesis methods	13a	Describe the processes used to decide which studies were eligible for each synthesis (e.g. tabulating the study intervention characteristics and comparing against the planned groups for each synthesis (item #5)).	Yes
	13b	Describe any methods required to prepare the data for presentation or synthesis, such as handling of missing summary statistics, or data conversions.	Yes
	13c	Describe any methods used to tabulate or visually display results of individual studies and syntheses.	Yes
	13d	Describe any methods used to synthesize results and provide a rationale for the choice(s). If meta-analysis was performed, describe the model(s), method(s) to identify the presence and extent of statistical heterogeneity, and software package(s) used.	No
	13e	Describe any methods used to explore possible causes of heterogeneity among study results (e.g. subgroup analysis, meta-regression).	No
	13f	Describe any sensitivity analyses conducted to assess robustness of the synthesized results.	Yes
Reporting bias assessment	14	Describe any methods used to assess risk of bias due to missing results in a synthesis (arising from reporting biases).	No
Certainty assessment	15	Describe any methods used to assess certainty (or confidence) in the body of evidence for an outcome.	Yes
**RESULTS**			
Study selection	16a	Describe the results of the search and selection process, from the number of records identified in the search to the number of studies included in the review, ideally using a flow diagram.	Yes
	16b	Cite studies that might appear to meet the inclusion criteria, but which were excluded, and explain why they were excluded.	Yes
Study characteristics	17	Cite each included study and present its characteristics.	Yes
Risk of bias in studies	18	Present assessments of risk of bias for each included study.	Yes
Results of individual studies	19	For all outcomes, present, for each study: (a) summary statistics for each group (where appropriate) and (b) an effect estimate and its precision (e.g. confidence/credible interval), ideally using structured tables or plots.	Yes
Results of syntheses	20a	For each synthesis, briefly summarise the characteristics and risk of bias among contributing studies.	No
	20b	Present results of all statistical syntheses conducted. If meta-analysis was done, present for each the summary estimate and its precision (e.g. confidence/credible interval) and measures of statistical heterogeneity. If comparing groups, describe the direction of the effect.	No
	20c	Present results of all investigations of possible causes of heterogeneity among study results.	Yes
	20d	Present results of all sensitivity analyses conducted to assess the robustness of the synthesized results.	Yes
Reporting biases	21	Present assessments of risk of bias due to missing results (arising from reporting biases) for each synthesis assessed.	No
Certainty of evidence	22	Present assessments of certainty (or confidence) in the body of evidence for each outcome assessed.	Yes
**DISCUSSION**			

Section and Topic	Item #	Checklist item	Location where item is reported
Discussion	23a	Provide a general interpretation of the results in the context of other evidence.	Yes
	23b	Discuss any limitations of the evidence included in the review.	Yes
	23c	Discuss any limitations of the review processes used.	Yes
	23d	Discuss implications of the results for practice, policy, and future research.	No
**OTHER INFORMATION**			
Registration and protocol	24a	Provide registration information for the review, including register name and registration number, or state that the review was not registered.	Yes
	24b	Indicate where the review protocol can be accessed, or state that a protocol was not prepared.	Yes
	24c	Describe and explain any amendments to information provided at registration or in the protocol.	No
Support	25	Describe sources of financial or non-financial support for the review, and the role of the funders or sponsors in the review.	No
Competing interests	26	Declare any competing interests of review authors.	No
Availability of data, code and other materials	27	Report which of the following are publicly available and where they can be found: template data collection forms; data extracted from included studies; data used for all analyses; analytic code; any other materials used in the review.	Yes

From: Page MJ, McKenzie JE, Bossuyt PM, Boutron I, Hoffmann TC, Mulrow CD, et al. The PRISMA 2020 statement: an updated guideline for reporting systematic reviews. BMJ 2021;372:n71. doi: 10.1136/bmj.n71

For more information, visit: http://www.prisma-statement.org/

Artikel 9

Section and Topic	Item #	Checklist item	Location where item is reported
**TITLE**			
Title	1	Identify the report as a systematic review.	Factors Associated with Health-Related Quality of Life among Jordanian Patients with Diabetic Foot Ulcer
**ABSTRACT**			
Abstract	2	See the PRISMA 2020 for Abstracts checklist.	The authors has well described it
**INTRODUCTION**			
Rationale	3	Describe the rationale for the review in the context of existing knowledge.	Yes
Objectives	4	Provide an explicit statement of the objective(s) or question(s) the review addresses.	Yes
**METHODS**			
Eligibility criteria	5	Specify the inclusion and exclusion criteria for the review and how studies were grouped for the syntheses.	Yes
Information sources	6	Specify all databases, registers, websites, organisations, reference lists and other sources searched or consulted to identify studies. Specify the date when each source was last searched or consulted.	Yes
Search strategy	7	Present the full search strategies for all databases, registers and websites, including any filters and limits used.	Yes
Selection process	8	Specify the methods used to decide whether a study met the inclusion criteria of the review, including how many reviewers screened each record and each report retrieved, whether they worked independently, and if applicable, details of automation tools used in the process.	Yes

Section and Topic	Item #	Checklist item	Location where item is reported
Data collection process	9	Specify the methods used to collect data from reports, including how many reviewers collected data from each report, whether they worked independently, any processes for obtaining or confirming data from study investigators, and if applicable, details of automation tools used in the process.	Yes
Data items	10a	List and define all outcomes for which data were sought. Specify whether all results that were compatible with each outcome domain in each study were sought (e.g. for all measures, time points, analyses), and if not, the methods used to decide which results to collect.	Yes
	10b	List and define all other variables for which data were sought (e.g. participant and intervention characteristics, funding sources). Describe any assumptions made about any missing or unclear information.	Yes
Study risk of bias assessment	11	Specify the methods used to assess risk of bias in the included studies, including details of the tool(s) used, how many reviewers assessed each study and whether they worked independently, and if applicable, details of automation tools used in the process.	No
Effect measures	12	Specify for each outcome the effect measure(s) (e.g. risk ratio, mean difference) used in the synthesis or presentation of results.	Yes
Synthesis methods	13a	Describe the processes used to decide which studies were eligible for each synthesis (e.g. tabulating the study intervention characteristics and comparing against the planned groups for each synthesis (item #5)).	Yes
	13b	Describe any methods required to prepare the data for presentation or synthesis, such as handling of missing summary statistics, or data conversions.	Yes
	13c	Describe any methods used to tabulate or visually display results of individual studies and syntheses.	Yes
	13d	Describe any methods used to synthesize results and provide a rationale for the choice(s). If meta-analysis was performed, describe the model(s), method(s) to identify the presence and extent of statistical heterogeneity, and software package(s) used.	No
	13e	Describe any methods used to explore possible causes of heterogeneity among study results (e.g. subgroup analysis, meta-regression).	No
	13f	Describe any sensitivity analyses conducted to assess robustness of the synthesized results.	Yes
Reporting bias assessment	14	Describe any methods used to assess risk of bias due to missing results in a synthesis (arising from reporting biases).	No
Certainty assessment	15	Describe any methods used to assess certainty (or confidence) in the body of evidence for an outcome.	Yes
**RESULTS**			
Study selection	16a	Describe the results of the search and selection process, from the number of records identified in the search to the number of studies included in the review, ideally using a flow diagram.	Yes
	16b	Cite studies that might appear to meet the inclusion criteria, but which were excluded, and explain why they were excluded.	Yes
Study characteristics	17	Cite each included study and present its characteristics.	Yes
Risk of bias in studies	18	Present assessments of risk of bias for each included study.	No
Results of individual studies	19	For all outcomes, present, for each study: (a) summary statistics for each group (where appropriate) and (b) an effect estimate and its precision (e.g. confidence/credible interval), ideally using structured tables or plots.	Yes
Results of syntheses	20a	For each synthesis, briefly summarise the characteristics and risk of bias among contributing studies.	No

Section and Topic	Item #	Checklist item	Location where item is reported
	20b	Present results of all statistical syntheses conducted. If meta-analysis was done, present for each the summary estimate and its precision (e.g. confidence/credible interval) and measures of statistical heterogeneity. If comparing groups, describe the direction of the effect.	Yes
	20c	Present results of all investigations of possible causes of heterogeneity among study results.	Yes
	20d	Present results of all sensitivity analyses conducted to assess the robustness of the synthesized results.	Yes
Reporting biases	21	Present assessments of risk of bias due to missing results (arising from reporting biases) for each synthesis assessed.	No
Certainty of evidence	22	Present assessments of certainty (or confidence) in the body of evidence for each outcome assessed.	Yes
**DISCUSSION**			
Discussion	23a	Provide a general interpretation of the results in the context of other evidence.	Yes
	23b	Discuss any limitations of the evidence included in the review.	Yes
	23c	Discuss any limitations of the review processes used.	Yes
	23d	Discuss implications of the results for practice, policy, and future research.	Yes
**OTHER INFORMATION**			
Registration and protocol	24a	Provide registration information for the review, including register name and registration number, or state that the review was not registered.	Yes
	24b	Indicate where the review protocol can be accessed, or state that a protocol was not prepared.	Yes
	24c	Describe and explain any amendments to information provided at registration or in the protocol.	No
Support	25	Describe sources of financial or non-financial support for the review, and the role of the funders or sponsors in the review.	No
Competing interests	26	Declare any competing interests of review authors.	Yes
Availability of data, code and other materials	27	Report which of the following are publicly available and where they can be found: template data collection forms; data extracted from included studies; data used for all analyses; analytic code; any other materials used in the review.	Yes

From: Page MJ, McKenzie JE, Bossuyt PM, Boutron I, Hoffmann TC, Mulrow CD, et al. The PRISMA 2020 statement: an updated guideline for reporting systematic reviews. BMJ 2021;372:n71. doi: 10.1136/bmj.n71
For more information, visit: http://www.prisma-statement.org/

Artikel 10

Section and Topic	Item #	Checklist item	Location where item is reported
**TITLE**			
Title	1	Identify the report as a systematic review.	A complementary and alternative medicine used among diabetes mellitus patient
**ABSTRACT**			
Abstract	2	See the PRISMA 2020 for Abstracts checklist.	The authors has well described it
**INTRODUCTION**			
Rationale	3	Describe the rationale for the review in the context of existing knowledge.	Yes
Objectives	4	Provide an explicit statement of the objective(s) or question(s) the review addresses.	Yes

Section and Topic	Item #	Checklist item	Location where item is reported
**METHODS**			
Eligibility criteria	5	Specify the inclusion and exclusion criteria for the review and how studies were grouped for the syntheses.	Yes
Information sources	6	Specify all databases, registers, websites, organisations, reference lists and other sources searched or consulted to identify studies. Specify the date when each source was last searched or consulted.	Yes
Search strategy	7	Present the full search strategies for all databases, registers and websites, including any filters and limits used.	Yes
Selection process	8	Specify the methods used to decide whether a study met the inclusion criteria of the review, including how many reviewers screened each record and each report retrieved, whether they worked independently, and if applicable, details of automation tools used in the process.	Yes
Data collection process	9	Specify the methods used to collect data from reports, including how many reviewers collected data from each report, whether they worked independently, any processes for obtaining or confirming data from study investigators, and if applicable, details of automation tools used in the process.	Yes
Data items	10a	List and define all outcomes for which data were sought. Specify whether all results that were compatible with each outcome domain in each study were sought (e.g. for all measures, time points, analyses), and if not, the methods used to decide which results to collect.	Yes
	10b	List and define all other variables for which data were sought (e.g. participant and intervention characteristics, funding sources). Describe any assumptions made about any missing or unclear information.	Yes
Study risk of bias assessment	11	Specify the methods used to assess risk of bias in the included studies, including details of the tool(s) used, how many reviewers assessed each study and whether they worked independently, and if applicable, details of automation tools used in the process.	No
Effect measures	12	Specify for each outcome the effect measure(s) (e.g. risk ratio, mean difference) used in the synthesis or presentation of results.	Yes
Synthesis methods	13a	Describe the processes used to decide which studies were eligible for each synthesis (e.g. tabulating the study intervention characteristics and comparing against the planned groups for each synthesis (item #5)).	Yes
	13b	Describe any methods required to prepare the data for presentation or synthesis, such as handling of missing summary statistics, or data conversions.	Yes
	13c	Describe any methods used to tabulate or visually display results of individual studies and syntheses.	Yes
	13d	Describe any methods used to synthesize results and provide a rationale for the choice(s). If meta-analysis was performed, describe the model(s), method(s) to identify the presence and extent of statistical heterogeneity, and software package(s) used.	Yes
	13e	Describe any methods used to explore possible causes of heterogeneity among study results (e.g. subgroup analysis, meta-regression).	No
	13f	Describe any sensitivity analyses conducted to assess robustness of the synthesized results.	Yes
Reporting bias assessment	14	Describe any methods used to assess risk of bias due to missing results in a synthesis (arising from reporting biases).	No
Certainty assessment	15	Describe any methods used to assess certainty (or confidence) in the body of evidence for an outcome.	Yes
**RESULTS**			
Study selection	16a	Describe the results of the search and selection process, from the number of records identified in the search to the number of studies included in the review, ideally using a flow diagram.	Yes

Section and Topic	Item #	Checklist item	Location where item is reported
	16b	Cite studies that might appear to meet the inclusion criteria, but which were excluded, and explain why they were excluded.	Yes
Study characteristics	17	Cite each included study and present its characteristics.	Yes
Risk of bias in studies	18	Present assessments of risk of bias for each included study.	No
Results of individual studies	19	For all outcomes, present, for each study: (a) summary statistics for each group (where appropriate) and (b) an effect estimate and its precision (e.g. confidence/credible interval), ideally using structured tables or plots.	Yes
Results of syntheses	20a	For each synthesis, briefly summarise the characteristics and risk of bias among contributing studies.	Yes
	20b	Present results of all statistical syntheses conducted. If meta-analysis was done, present for each the summary estimate and its precision (e.g. confidence/credible interval) and measures of statistical heterogeneity. If comparing groups, describe the direction of the effect.	Yes
	20c	Present results of all investigations of possible causes of heterogeneity among study results.	Yes
	20d	Present results of all sensitivity analyses conducted to assess the robustness of the synthesized results.	Yes
Reporting biases	21	Present assessments of risk of bias due to missing results (arising from reporting biases) for each synthesis assessed.	No
Certainty of evidence	22	Present assessments of certainty (or confidence) in the body of evidence for each outcome assessed.	Yes
**DISCUSSION**			
Discussion	23a	Provide a general interpretation of the results in the context of other evidence.	Yes
	23b	Discuss any limitations of the evidence included in the review.	Yes
	23c	Discuss any limitations of the review processes used.	No
	23d	Discuss implications of the results for practice, policy, and future research.	Yes
**OTHER INFORMATION**			
Registration and protocol	24a	Provide registration information for the review, including register name and registration number, or state that the review was not registered.	Yes
	24b	Indicate where the review protocol can be accessed, or state that a protocol was not prepared.	Yes
	24c	Describe and explain any amendments to information provided at registration or in the protocol.	No
Support	25	Describe sources of financial or non-financial support for the review, and the role of the funders or sponsors in the review.	No described
Competing interests	26	Declare any competing interests of review authors.	Yes
Availability of data, code and other materials	27	Report which of the following are publicly available and where they can be found: template data collection forms; data extracted from included studies; data used for all analyses; analytic code; any other materials used in the review.	Yes

From: Page MJ, McKenzie JE, Bossuyt PM, Boutron I, Hoffmann TC, Mulrow CD, et al. The PRISMA 2020 statement: an updated guideline for reporting systematic reviews. BMJ 2021;372:n71. doi: 10.1136/bmj.n71

For more information, visit: http://www.prisma-statement.org/

Artikel 11

Section and Topic	Item #	Checklist item	Location where item is reported
**TITLE**			

Section and Topic	Item #	Checklist item	Location where item is reported
Title	1	Identify the report as a systematic review.	Diabetes Prevalence and Quality of Life of Female Nursing Students
**ABSTRACT**			
Abstract	2	See the PRISMA 2020 for Abstracts checklist.	The authors has well described it
**INTRODUCTION**			
Rationale	3	Describe the rationale for the review in the context of existing knowledge.	Yes
Objectives	4	Provide an explicit statement of the objective(s) or question(s) the review addresses.	Yes
**METHODS**			
Eligibility criteria	5	Specify the inclusion and exclusion criteria for the review and how studies were grouped for the syntheses.	Yes
Information sources	6	Specify all databases, registers, websites, organisations, reference lists and other sources searched or consulted to identify studies. Specify the date when each source was last searched or consulted.	Yes
Search strategy	7	Present the full search strategies for all databases, registers and websites, including any filters and limits used.	Yes
Selection process	8	Specify the methods used to decide whether a study met the inclusion criteria of the review, including how many reviewers screened each record and each report retrieved, whether they worked independently, and if applicable, details of automation tools used in the process.	Yes
Data collection process	9	Specify the methods used to collect data from reports, including how many reviewers collected data from each report, whether they worked independently, any processes for obtaining or confirming data from study investigators, and if applicable, details of automation tools used in the process.	Yes
Data items	10a	List and define all outcomes for which data were sought. Specify whether all results that were compatible with each outcome domain in each study were sought (e.g. for all measures, time points, analyses), and if not, the methods used to decide which results to collect.	Yes
	10b	List and define all other variables for which data were sought (e.g. participant and intervention characteristics, funding sources). Describe any assumptions made about any missing or unclear information.	Yes
Study risk of bias assessment	11	Specify the methods used to assess risk of bias in the included studies, including details of the tool(s) used, how many reviewers assessed each study and whether they worked independently, and if applicable, details of automation tools used in the process.	No
Effect measures	12	Specify for each outcome the effect measure(s) (e.g. risk ratio, mean difference) used in the synthesis or presentation of results.	Yes
Synthesis methods	13a	Describe the processes used to decide which studies were eligible for each synthesis (e.g. tabulating the study intervention characteristics and comparing against the planned groups for each synthesis (item #5)).	Yes
	13b	Describe any methods required to prepare the data for presentation or synthesis, such as handling of missing summary statistics, or data conversions.	Yes
	13c	Describe any methods used to tabulate or visually display results of individual studies and syntheses.	Yes
	13d	Describe any methods used to synthesize results and provide a rationale for the choice(s). If meta-analysis was performed, describe the model(s), method(s) to identify the presence and extent of statistical heterogeneity, and software package(s) used.	No
	13e	Describe any methods used to explore possible causes of heterogeneity among study results (e.g. subgroup analysis, meta-regression).	No
	13f	Describe any sensitivity analyses conducted to assess robustness of the syn-	

Section and Topic	Item #	Checklist item	Location where item is reported
		thesized results.	
Reporting bias assessment	14	Describe any methods used to assess risk of bias due to missing results in a synthesis (arising from reporting biases).	No
Certainty assessment	15	Describe any methods used to assess certainty (or confidence) in the body of evidence for an outcome.	Yes
**RESULTS**			
Study selection	16a	Describe the results of the search and selection process, from the number of records identified in the search to the number of studies included in the review, ideally using a flow diagram.	No
	16b	Cite studies that might appear to meet the inclusion criteria, but which were excluded, and explain why they were excluded.	Yes
Study characteristics	17	Cite each included study and present its characteristics.	Yes
Risk of bias in studies	18	Present assessments of risk of bias for each included study.	No
Results of individual studies	19	For all outcomes, present, for each study: (a) summary statistics for each group (where appropriate) and (b) an effect estimate and its precision (e.g. confidence/credible interval), ideally using structured tables or plots.	No
Results of syntheses	20a	For each synthesis, briefly summarise the characteristics and risk of bias among contributing studies.	Yes
	20b	Present results of all statistical syntheses conducted. If meta-analysis was done, present for each the summary estimate and its precision (e.g. confidence/credible interval) and measures of statistical heterogeneity. If comparing groups, describe the direction of the effect.	No
	20c	Present results of all investigations of possible causes of heterogeneity among study results.	Yes
	20d	Present results of all sensitivity analyses conducted to assess the robustness of the synthesized results.	Yes
Reporting biases	21	Present assessments of risk of bias due to missing results (arising from reporting biases) for each synthesis assessed.	No
Certainty of evidence	22	Present assessments of certainty (or confidence) in the body of evidence for each outcome assessed.	Yes
**DISCUSSION**			
Discussion	23a	Provide a general interpretation of the results in the context of other evidence.	Yes
	23b	Discuss any limitations of the evidence included in the review.	Yes
	23c	Discuss any limitations of the review processes used.	Yes
	23d	Discuss implications of the results for practice, policy, and future research.	Yes
**OTHER INFORMATION**			
Registration and protocol	24a	Provide registration information for the review, including register name and registration number, or state that the review was not registered.	Yes
	24b	Indicate where the review protocol can be accessed, or state that a protocol was not prepared.	Yes
	24c	Describe and explain any amendments to information provided at registration or in the protocol.	Mo described
Support	25	Describe sources of financial or non-financial support for the review, and the role of the funders or sponsors in the review.	No described
Competing interests	26	Declare any competing interests of review authors.	No described
Availability of data, code and other materials	27	Report which of the following are publicly available and where they can be found: template data collection forms; data extracted from included studies; data used for all analyses; analytic code; any other materials used in the review.	Yes

From: Page MJ, McKenzie JE, Bossuyt PM, Boutron I, Hoffmann TC, Mulrow CD, et al. The PRISMA 2020 statement: an updated guideline for reporting systematic reviews. BMJ 2021;372:n71. doi: 10.1136/bmj.n71
For more information, visit: http://www.prisma-statement.org/

Artikel 12

Section and Topic	Item #	Checklist item	Location where item is reported
**TITLE**			
Title	1	Identify the report as a systematic review.	The correlation of fasting blood glucose levels with the severity of diabetic foot ulcers and the outcome of treatment strategies
**ABSTRACT**			
Abstract	2	See the PRISMA 2020 for Abstracts checklist.	The authors has well described it
**INTRODUCTION**			
Rationale	3	Describe the rationale for the review in the context of existing knowledge.	Yes
Objectives	4	Provide an explicit statement of the objective(s) or question(s) the review addresses.	Yes
**METHODS**			
Eligibility criteria	5	Specify the inclusion and exclusion criteria for the review and how studies were grouped for the syntheses.	Yes
Information sources	6	Specify all databases, registers, websites, organisations, reference lists and other sources searched or consulted to identify studies. Specify the date when each source was last searched or consulted.	Yes
Search strategy	7	Present the full search strategies for all databases, registers and websites, including any filters and limits used.	Yes
Selection process	8	Specify the methods used to decide whether a study met the inclusion criteria of the review, including how many reviewers screened each record and each report retrieved, whether they worked independently, and if applicable, details of automation tools used in the process.	Yes
Data collection process	9	Specify the methods used to collect data from reports, including how many reviewers collected data from each report, whether they worked independently, any processes for obtaining or confirming data from study investigators, and if applicable, details of automation tools used in the process.	Yes
Data items	10a	List and define all outcomes for which data were sought. Specify whether all results that were compatible with each outcome domain in each study were sought (e.g. for all measures, time points, analyses), and if not, the methods used to decide which results to collect.	No
	10b	List and define all other variables for which data were sought (e.g. participant and intervention characteristics, funding sources). Describe any assumptions made about any missing or unclear information.	Yes
Study risk of bias assessment	11	Specify the methods used to assess risk of bias in the included studies, including details of the tool(s) used, how many reviewers assessed each study and whether they worked independently, and if applicable, details of automation tools used in the process.	Yes
Effect measures	12	Specify for each outcome the effect measure(s) (e.g. risk ratio, mean difference) used in the synthesis or presentation of results.	Yes
Synthesis methods	13a	Describe the processes used to decide which studies were eligible for each synthesis (e.g. tabulating the study intervention characteristics and comparing against the planned groups for each synthesis (item #5)).	Yes
	13b	Describe any methods required to prepare the data for presentation or synthesis, such as handling of missing summary statistics, or data conversions.	Yes

Section and Topic	Item #	Checklist item	Location where item is reported
	13c	Describe any methods used to tabulate or visually display results of individual studies and syntheses.	Yes
	13d	Describe any methods used to synthesize results and provide a rationale for the choice(s). If meta-analysis was performed, describe the model(s), method(s) to identify the presence and extent of statistical heterogeneity, and software package(s) used.	Yes
	13e	Describe any methods used to explore possible causes of heterogeneity among study results (e.g. subgroup analysis, meta-regression).	Yes
	13f	Describe any sensitivity analyses conducted to assess robustness of the synthesized results.	Yes
Reporting bias assessment	14	Describe any methods used to assess risk of bias due to missing results in a synthesis (arising from reporting biases).	No
Certainty assessment	15	Describe any methods used to assess certainty (or confidence) in the body of evidence for an outcome.	Yes
**RESULTS**			
Study selection	16a	Describe the results of the search and selection process, from the number of records identified in the search to the number of studies included in the review, ideally using a flow diagram.	Yes
	16b	Cite studies that might appear to meet the inclusion criteria, but which were excluded, and explain why they were excluded.	Yes
Study characteristics	17	Cite each included study and present its characteristics.	Yes
Risk of bias in studies	18	Present assessments of risk of bias for each included study.	Yes
Results of individual studies	19	For all outcomes, present, for each study: (a) summary statistics for each group (where appropriate) and (b) an effect estimate and its precision (e.g. confidence/credible interval), ideally using structured tables or plots.	No
Results of syntheses	20a	For each synthesis, briefly summarise the characteristics and risk of bias among contributing studies.	Yes
	20b	Present results of all statistical syntheses conducted. If meta-analysis was done, present for each the summary estimate and its precision (e.g. confidence/credible interval) and measures of statistical heterogeneity. If comparing groups, describe the direction of the effect.	Yes
	20c	Present results of all investigations of possible causes of heterogeneity among study results.	Yes
	20d	Present results of all sensitivity analyses conducted to assess the robustness of the synthesized results.	Yes
Reporting biases	21	Present assessments of risk of bias due to missing results (arising from reporting biases) for each synthesis assessed.	No
Certainty of evidence	22	Present assessments of certainty (or confidence) in the body of evidence for each outcome assessed.	Yes
**DISCUSSION**			
Discussion	23a	Provide a general interpretation of the results in the context of other evidence.	Yes
	23b	Discuss any limitations of the evidence included in the review.	Yes
	23c	Discuss any limitations of the review processes used.	Yes
	23d	Discuss implications of the results for practice, policy, and future research.	Yes
**OTHER INFORMATION**			
Registration and	24a	Provide registration information for the review, including register name and registration number, or state that the review was not regis-	Yes

Section and Topic	Item #	Checklist item	Location where item is reported
protocol		tered.	
	24b	Indicate where the review protocol can be accessed, or state that a protocol was not prepared.	Yes
	24c	Describe and explain any amendments to information provided at registration or in the protocol.	No described
Support	25	Describe sources of financial or non-financial support for the review, and the role of the funders or sponsors in the review.	No described
Competing interests	26	Declare any competing interests of review authors.	Yes
Availability of data, code and other materials	27	Report which of the following are publicly available and where they can be found: template data collection forms; data extracted from included studies; data used for all analyses; analytic code; any other materials used in the review.	

From: Page MJ, McKenzie JE, Bossuyt PM, Boutron I, Hoffmann TC, Mulrow CD, et al. The PRISMA 2020 statement: an updated guideline for reporting systematic reviews. BMJ 2021;372:n71. doi: 10.1136/bmj.n71

For more information, visit: http://www.prisma-statement.org/

Artikel 13.

Section and Topic	Item #	Checklist item	Location where item is reported
**TITLE**			
Title	1	Identify the report as a systematic review.	Discovering the benefits of yoga and improve quality of life
**ABSTRACT**			
Abstract	2	See the PRISMA 2020 for Abstracts checklist.	The authors has well described it
**INTRODUCTION**			
Rationale	3	Describe the rationale for the review in the context of existing knowledge.	Yes
Objectives	4	Provide an explicit statement of the objective(s) or question(s) the review addresses.	Yes
**METHODS**			
Eligibility criteria	5	Specify the inclusion and exclusion criteria for the review and how studies were grouped for the syntheses.	Yes
Information sources	6	Specify all databases, registers, websites, organisations, reference lists and other sources searched or consulted to identify studies. Specify the date when each source was last searched or consulted.	Yes
Search strategy	7	Present the full search strategies for all databases, registers and websites, including any filters and limits used.	No
Selection process	8	Specify the methods used to decide whether a study met the inclusion criteria of the review, including how many reviewers screened each record and each report retrieved, whether they worked independently, and if applicable, details of automation tools used in the process.	Yes
Data collection process	9	Specify the methods used to collect data from reports, including how many reviewers collected data from each report, whether they worked independently, any processes for obtaining or confirming data from study investigators, and if applicable, details of automation tools used in the process.	Yes
Data items	10a	List and define all outcomes for which data were sought. Specify whether all results that were compatible with each outcome domain in each study were sought (e.g. for all measures, time points, analyses), and if not, the methods used to decide which results to collect.	Yes
	10b	List and define all other variables for which data were sought (e.g. participant and intervention characteristics, funding sources). Describe any assumptions made about any missing or unclear information.	No
Study risk of bias	11	Specify the methods used to assess risk of bias in the included studies, including details of the tool(s) used, how many reviewers assessed each study and	No

Section and Topic	Item #	Checklist item	Location where item is reported
assessment		whether they worked independently, and if applicable, details of automation tools used in the process.	
Effect measures	12	Specify for each outcome the effect measure(s) (e.g. risk ratio, mean difference) used in the synthesis or presentation of results.	Yes
Synthesis methods	13a	Describe the processes used to decide which studies were eligible for each synthesis (e.g. tabulating the study intervention characteristics and comparing against the planned groups for each synthesis (item #5)).	Yes
	13b	Describe any methods required to prepare the data for presentation or synthesis, such as handling of missing summary statistics, or data conversions.	Yes
	13c	Describe any methods used to tabulate or visually display results of individual studies and syntheses.	Yes
	13d	Describe any methods used to synthesize results and provide a rationale for the choice(s). If meta-analysis was performed, describe the model(s), method(s) to identify the presence and extent of statistical heterogeneity, and software package(s) used.	No
	13e	Describe any methods used to explore possible causes of heterogeneity among study results (e.g. subgroup analysis, meta-regression).	Yes
	13f	Describe any sensitivity analyses conducted to assess robustness of the synthesized results.	Yes
Reporting bias assessment	14	Describe any methods used to assess risk of bias due to missing results in a synthesis (arising from reporting biases).	Yes
Certainty assessment	15	Describe any methods used to assess certainty (or confidence) in the body of evidence for an outcome.	Yes
**RESULTS**			
Study selection	16a	Describe the results of the search and selection process, from the number of records identified in the search to the number of studies included in the review, ideally using a flow diagram.	Yes
	16b	Cite studies that might appear to meet the inclusion criteria, but which were excluded, and explain why they were excluded.	Yes
Study characteristics	17	Cite each included study and present its characteristics.	Yes
Risk of bias in studies	18	Present assessments of risk of bias for each included study.	No
Results of individual studies	19	For all outcomes, present, for each study: (a) summary statistics for each group (where appropriate) and (b) an effect estimate and its precision (e.g. confidence/credible interval), ideally using structured tables or plots.	Yes
Results of syntheses	20a	For each synthesis, briefly summarise the characteristics and risk of bias among contributing studies.	Yes
	20b	Present results of all statistical syntheses conducted. If meta-analysis was done, present for each the summary estimate and its precision (e.g. confidence/credible interval) and measures of statistical heterogeneity. If comparing groups, describe the direction of the effect.	No
	20c	Present results of all investigations of possible causes of heterogeneity among study results.	Yes
	20d	Present results of all sensitivity analyses conducted to assess the robustness of the synthesized results.	Yes
Reporting biases	21	Present assessments of risk of bias due to missing results (arising from reporting biases) for each synthesis assessed.	No
Certainty of evidence	22	Present assessments of certainty (or confidence) in the body of evidence for each outcome assessed.	Yes
**DISCUSSION**			
Discussion	23a	Provide a general interpretation of the results in the context of other evidence.	Yes
	23b	Discuss any limitations of the evidence included in the review.	Yes

Section and Topic	Item #	Checklist item	Location where item is reported
	23c	Discuss any limitations of the review processes used.	Yes
	23d	Discuss implications of the results for practice, policy, and future research.	Yes
**OTHER INFORMATION**			
Registration and protocol	24a	Provide registration information for the review, including register name and registration number, or state that the review was not registered.	Yes
	24b	Indicate where the review protocol can be accessed, or state that a protocol was not prepared.	No
	24c	Describe and explain any amendments to information provided at registration or in the protocol.	No described
Support	25	Describe sources of financial or non-financial support for the review, and the role of the funders or sponsors in the review.	No described
Competing interests	26	Declare any competing interests of review authors.	Yes
Availability of data, code and other materials	27	Report which of the following are publicly available and where they can be found: template data collection forms; data extracted from included studies; data used for all analyses; analytic code; any other materials used in the review.	Yes

From: Page MJ, McKenzie JE, Bossuyt PM, Boutron I, Hoffmann TC, Mulrow CD, et al. The PRISMA 2020 statement: an updated guideline for reporting systematic reviews. BMJ 2021;372:n71. doi: 10.1136/bmj.n71

For more information, visit: http://www.prisma-statement.org/

Artikel 14.

Section and Topic	Item #	Checklist item	Location where item is reported
**TITLE**			
Title	1	Identify the report as a systematic review.	Protocol for a clinical trial of text messaging in addition to standard care versus standard care alone in prevention of type 2 diabetes through lifestyle modification in India and the UK
**ABSTRACT**			
Abstract	2	See the PRISMA 2020 for Abstracts checklist.	The authors has well described it
**INTRODUCTION**			
Rationale	3	Describe the rationale for the review in the context of existing knowledge.	Yes
Objectives	4	Provide an explicit statement of the objective(s) or question(s) the review addresses.	Yes
**METHODS**			
Eligibility criteria	5	Specify the inclusion and exclusion criteria for the review and how studies were grouped for the syntheses.	Yes
Information sources	6	Specify all databases, registers, websites, organisations, reference lists and other sources searched or consulted to identify studies. Specify the date when each source was last searched or consulted.	Yes
Search strategy	7	Present the full search strategies for all databases, registers and websites, including any filters and limits used.	Yes
Selection process	8	Specify the methods used to decide whether a study met the inclusion criteria of the review, including how many reviewers screened each record and each report retrieved, whether they worked independently, and if applicable, details of automation tools used in the process.	Yes
Data collection process	9	Specify the methods used to collect data from reports, including how many reviewers collected data from each report,	Yes

Section and Topic	Item #	Checklist item	Location where item is reported
		whether they worked independently, any processes for obtaining or confirming data from study investigators, and if applicable, details of automation tools used in the process.	
Data items	10a	List and define all outcomes for which data were sought. Specify whether all results that were compatible with each outcome domain in each study were sought (e.g. for all measures, time points, analyses), and if not, the methods used to decide which results to collect.	Yes
	10b	List and define all other variables for which data were sought (e.g. participant and intervention characteristics, funding sources). Describe any assumptions made about any missing or unclear information.	Yes
Study risk of bias assessment	11	Specify the methods used to assess risk of bias in the included studies, including details of the tool(s) used, how many reviewers assessed each study and whether they worked independently, and if applicable, details of automation tools used in the process.	No
Effect measures	12	Specify for each outcome the effect measure(s) (e.g. risk ratio, mean difference) used in the synthesis or presentation of results.	Yes
Synthesis methods	13a	Describe the processes used to decide which studies were eligible for each synthesis (e.g. tabulating the study intervention characteristics and comparing against the planned groups for each synthesis (item #5)).	Yes
	13b	Describe any methods required to prepare the data for presentation or synthesis, such as handling of missing summary statistics, or data conversions.	Yes
	13c	Describe any methods used to tabulate or visually display results of individual studies and syntheses.	Yes
	13d	Describe any methods used to synthesize results and provide a rationale for the choice(s). If meta-analysis was performed, describe the model(s), method(s) to identify the presence and extent of statistical heterogeneity, and software package(s) used.	Yes
	13e	Describe any methods used to explore possible causes of heterogeneity among study results (e.g. subgroup analysis, meta-regression).	Yes
	13f	Describe any sensitivity analyses conducted to assess robustness of the synthesized results.	Yes
Reporting bias assessment	14	Describe any methods used to assess risk of bias due to missing results in a synthesis (arising from reporting biases).	No
Certainty assessment	15	Describe any methods used to assess certainty (or confidence) in the body of evidence for an outcome.	Yes
**RESULTS**			
Study selection	16a	Describe the results of the search and selection process, from the number of records identified in the search to the number of studies included in the review, ideally using a flow diagram.	Yes
	16b	Cite studies that might appear to meet the inclusion criteria, but which were excluded, and explain why they were excluded.	Yes
Study characteristics	17	Cite each included study and present its characteristics.	Yes
Risk of bias in studies	18	Present assessments of risk of bias for each included study.	No
Results of individ-	19	For all outcomes, present, for each study: (a) summary statis-	Yes

Section and Topic	Item #	Checklist item	Location where item is reported
ual studies		tics for each group (where appropriate) and (b) an effect estimate and its precision (e.g. confidence/credible interval), ideally using structured tables or plots.	
Results of syntheses	20a	For each synthesis, briefly summarise the characteristics and risk of bias among contributing studies.	Yes
	20b	Present results of all statistical syntheses conducted. If meta-analysis was done, present for each the summary estimate and its precision (e.g. confidence/credible interval) and measures of statistical heterogeneity. If comparing groups, describe the direction of the effect.	Yes
	20c	Present results of all investigations of possible causes of heterogeneity among study results.	Yes
	20d	Present results of all sensitivity analyses conducted to assess the robustness of the synthesized results.	Yes
Reporting biases	21	Present assessments of risk of bias due to missing results (arising from reporting biases) for each synthesis assessed.	No
Certainty of evidence	22	Present assessments of certainty (or confidence) in the body of evidence for each outcome assessed.	Yes
**DISCUSSION**			
Discussion	23a	Provide a general interpretation of the results in the context of other evidence.	Yes
	23b	Discuss any limitations of the evidence included in the review.	Yes
	23c	Discuss any limitations of the review processes used.	Yes
	23d	Discuss implications of the results for practice, policy, and future research.	No
**OTHER INFORMATION**			
Registration and protocol	24a	Provide registration information for the review, including register name and registration number, or state that the review was not registered.	Yes
	24b	Indicate where the review protocol can be accessed, or state that a protocol was not prepared.	Yes
	24c	Describe and explain any amendments to information provided at registration or in the protocol.	No described
Support	25	Describe sources of financial or non-financial support for the review, and the role of the funders or sponsors in the review.	Yes
Competing interests	26	Declare any competing interests of review authors.	Yes
Availability of data, code and other materials	27	Report which of the following are publicly available and where they can be found: template data collection forms; data extracted from included studies; data used for all analyses; analytic code; any other materials used in the review.	Yes

From: Page MJ, McKenzie JE, Bossuyt PM, Boutron I, Hoffmann TC, Mulrow CD, et al. The PRISMA 2020 statement: an updated guideline for reporting systematic reviews. BMJ 2021;372:n71. doi: 10.1136/bmj.n71

For more information, visit: http://www.prisma-statement.org/

Artikel 15.

Section and Topic	Item #	Checklist item	Location where item is reported
**TITLE**			
Title	1	Identify the report as a systematic review.	The effect of diabetes self-management education on HbA1c and quality of life in African-Americans.
**ABSTRACT**			

Section and Topic	Item #	Checklist item	Location where item is reported
Abstract	2	See the PRISMA 2020 for Abstracts checklist.	The authors has well described it
**INTRODUCTION**			
Rationale	3	Describe the rationale for the review in the context of existing knowledge.	Yes
Objectives	4	Provide an explicit statement of the objective(s) or question(s) the review addresses.	Yes
**METHODS**			
Eligibility criteria	5	Specify the inclusion and exclusion criteria for the review and how studies were grouped for the syntheses.	Yes
Information sources	6	Specify all databases, registers, websites, organisations, reference lists and other sources searched or consulted to identify studies. Specify the date when each source was last searched or consulted.	Yes
Search strategy	7	Present the full search strategies for all databases, registers and websites, including any filters and limits used.	Yes
Selection process	8	Specify the methods used to decide whether a study met the inclusion criteria of the review, including how many reviewers screened each record and each report retrieved, whether they worked independently, and if applicable, details of automation tools used in the process.	Yes
Data collection process	9	Specify the methods used to collect data from reports, including how many reviewers collected data from each report, whether they worked independently, any processes for obtaining or confirming data from study investigators, and if applicable, details of automation tools used in the process.	Yes
Data items	10a	List and define all outcomes for which data were sought. Specify whether all results that were compatible with each outcome domain in each study were sought (e.g. for all measures, time points, analyses), and if not, the methods used to decide which results to collect.	Yes
	10b	List and define all other variables for which data were sought (e.g. participant and intervention characteristics, funding sources). Describe any assumptions made about any missing or unclear information.	Yes
Study risk of bias assessment	11	Specify the methods used to assess risk of bias in the included studies, including details of the tool(s) used, how many reviewers assessed each study and whether they worked independently, and if applicable, details of automation tools used in the process.	No
Effect measures	12	Specify for each outcome the effect measure(s) (e.g. risk ratio, mean difference) used in the synthesis or presentation of results.	Yes
Synthesis methods	13a	Describe the processes used to decide which studies were eligible for each synthesis (e.g. tabulating the study intervention characteristics and comparing against the planned groups for each synthesis (item #5)).	Yes
	13b	Describe any methods required to prepare the data for presentation or synthesis, such as handling of missing summary statistics, or data conversions.	Yes
	13c	Describe any methods used to tabulate or visually display results of individual studies and syntheses.	Yes
	13d	Describe any methods used to synthesize results and provide a rationale for the choice(s). If meta-analysis was performed, describe the model(s), method(s) to identify the presence and extent of statistical heterogeneity, and software package(s) used.	No
	13e	Describe any methods used to explore possible causes of heterogeneity among study results (e.g. subgroup analysis, meta-regression).	Yes
	13f	Describe any sensitivity analyses conducted to assess robustness of the synthesized results.	Yes
Reporting bias as-	14	Describe any methods used to assess risk of bias due to missing results	No

Section and Topic	Item #	Checklist item	Location where item is reported
sessment		in a synthesis (arising from reporting biases).	
Certainty assessment	15	Describe any methods used to assess certainty (or confidence) in the body of evidence for an outcome.	Yes
**RESULTS**			
Study selection	16a	Describe the results of the search and selection process, from the number of records identified in the search to the number of studies included in the review, ideally using a flow diagram.	Yes
	16b	Cite studies that might appear to meet the inclusion criteria, but which were excluded, and explain why they were excluded.	Yes
Study characteristics	17	Cite each included study and present its characteristics.	Yes
Risk of bias in studies	18	Present assessments of risk of bias for each included study.	No
Results of individual studies	19	For all outcomes, present, for each study: (a) summary statistics for each group (where appropriate) and (b) an effect estimate and its precision (e.g. confidence/credible interval), ideally using structured tables or plots.	Yes
Results of syntheses	20a	For each synthesis, briefly summarise the characteristics and risk of bias among contributing studies.	Yes
	20b	Present results of all statistical syntheses conducted. If meta-analysis was done, present for each the summary estimate and its precision (e.g. confidence/credible interval) and measures of statistical heterogeneity. If comparing groups, describe the direction of the effect.	Yes
	20c	Present results of all investigations of possible causes of heterogeneity among study results.	Yes
	20d	Present results of all sensitivity analyses conducted to assess the robustness of the synthesized results.	Yes
Reporting biases	21	Present assessments of risk of bias due to missing results (arising from reporting biases) for each synthesis assessed.	Yes
Certainty of evidence	22	Present assessments of certainty (or confidence) in the body of evidence for each outcome assessed.	Yes
**DISCUSSION**			
Discussion	23a	Provide a general interpretation of the results in the context of other evidence.	Yes
	23b	Discuss any limitations of the evidence included in the review.	Yes
	23c	Discuss any limitations of the review processes used.	Yes
	23d	Discuss implications of the results for practice, policy, and future research.	Yes
**OTHER INFORMATION**			
Registration and protocol	24a	Provide registration information for the review, including register name and registration number, or state that the review was not registered.	Yes
	24b	Indicate where the review protocol can be accessed, or state that a protocol was not prepared.	Yes
	24c	Describe and explain any amendments to information provided at registration or in the protocol.	No described
Support	25	Describe sources of financial or non-financial support for the review, and the role of the funders or sponsors in the review.	No described
Competing interests	26	Declare any competing interests of review authors.	Yes
Availability of data, code and other materials	27	Report which of the following are publicly available and where they can be found: template data collection forms; data extracted from included studies; data used for all analyses; analytic code; any other materials used in the review.	Yes

From: Page MJ, McKenzie JE, Bossuyt PM, Boutron I, Hoffmann TC, Mulrow CD, et al. The PRISMA 2020 statement: an updated guideline for reporting systematic reviews. BMJ 2021;372:n71. doi: 10.1136/bmj.n71
For more information, visit: http://www.prisma-statement.org/

Artikel 16.

Section and Topic	Item #	Checklist item	Location where item is reported
**TITLE**			
Title	1	Identify the report as a systematic review.	Efficacy Of Pilates Based Mat Exercise On Quality Of Life, Quality Of Sleep And Satisfaction With Life In Type 2 Diabetes Mellitus
**ABSTRACT**			
Abstract	2	See the PRISMA 2020 for Abstracts checklist.	The authors has well described it
**INTRODUCTION**			
Rationale	3	Describe the rationale for the review in the context of existing knowledge.	Yes
Objectives	4	Provide an explicit statement of the objective(s) or question(s) the review addresses.	Yes
**METHODS**			
Eligibility criteria	5	Specify the inclusion and exclusion criteria for the review and how studies were grouped for the syntheses.	Yes
Information sources	6	Specify all databases, registers, websites, organisations, reference lists and other sources searched or consulted to identify studies. Specify the date when each source was last searched or consulted.	Yes
Search strategy	7	Present the full search strategies for all databases, registers and websites, including any filters and limits used.	Yes
Selection process	8	Specify the methods used to decide whether a study met the inclusion criteria of the review, including how many reviewers screened each record and each report retrieved, whether they worked independently, and if applicable, details of automation tools used in the process.	Yes
Data collection process	9	Specify the methods used to collect data from reports, including how many reviewers collected data from each report, whether they worked independently, any processes for obtaining or confirming data from study investigators, and if applicable, details of automation tools used in the process.	Yes
Data items	10a	List and define all outcomes for which data were sought. Specify whether all results that were compatible with each outcome domain in each study were sought (e.g. for all measures, time points, analyses), and if not, the methods used to decide which results to collect.	Yes
	10b	List and define all other variables for which data were sought (e.g. participant and intervention characteristics, funding sources). Describe any assumptions made about any missing or unclear information.	No
Study risk of bias assessment	11	Specify the methods used to assess risk of bias in the included studies, including details of the tool(s) used, how many reviewers assessed each study and whether they worked independently, and if applicable, details of automation tools used in the process.	No
Effect measures	12	Specify for each outcome the effect measure(s) (e.g. risk ratio, mean difference) used in the synthesis or presentation of results.	Yes
Synthesis methods	13a	Describe the processes used to decide which studies were eligible for each synthesis (e.g. tabulating the study intervention characteristics and comparing against the planned groups for each synthesis (item #5)).	Yes
	13b	Describe any methods required to prepare the data for presentation or synthesis, such as handling of missing summary statistics, or data	Yes

Section and Topic	Item #	Checklist item	Location where item is reported
		conversions.	
	13c	Describe any methods used to tabulate or visually display results of individual studies and syntheses.	Yes
	13d	Describe any methods used to synthesize results and provide a rationale for the choice(s). If meta-analysis was performed, describe the model(s), method(s) to identify the presence and extent of statistical heterogeneity, and software package(s) used.	Yes
	13e	Describe any methods used to explore possible causes of heterogeneity among study results (e.g. subgroup analysis, meta-regression).	No
	13f	Describe any sensitivity analyses conducted to assess robustness of the synthesized results.	Yes
Reporting bias assessment	14	Describe any methods used to assess risk of bias due to missing results in a synthesis (arising from reporting biases).	No
Certainty assessment	15	Describe any methods used to assess certainty (or confidence) in the body of evidence for an outcome.	Yes
**RESULTS**			
Study selection	16a	Describe the results of the search and selection process, from the number of records identified in the search to the number of studies included in the review, ideally using a flow diagram.	Yes
	16b	Cite studies that might appear to meet the inclusion criteria, but which were excluded, and explain why they were excluded.	Yes
Study characteristics	17	Cite each included study and present its characteristics.	Yes
Risk of bias in studies	18	Present assessments of risk of bias for each included study.	No
Results of individual studies	19	For all outcomes, present, for each study: (a) summary statistics for each group (where appropriate) and (b) an effect estimate and its precision (e.g. confidence/credible interval), ideally using structured tables or plots.	Yes
Results of syntheses	20a	For each synthesis, briefly summarise the characteristics and risk of bias among contributing studies.	Yes
	20b	Present results of all statistical syntheses conducted. If meta-analysis was done, present for each the summary estimate and its precision (e.g. confidence/credible interval) and measures of statistical heterogeneity. If comparing groups, describe the direction of the effect.	No
	20c	Present results of all investigations of possible causes of heterogeneity among study results.	Yes
	20d	Present results of all sensitivity analyses conducted to assess the robustness of the synthesized results.	Yes
Reporting biases	21	Present assessments of risk of bias due to missing results (arising from reporting biases) for each synthesis assessed.	No
Certainty of evidence	22	Present assessments of certainty (or confidence) in the body of evidence for each outcome assessed.	Yes
**DISCUSSION**			
Discussion	23a	Provide a general interpretation of the results in the context of other evidence.	Yes
	23b	Discuss any limitations of the evidence included in the review.	Yes
	23c	Discuss any limitations of the review processes used.	Yes
	23d	Discuss implications of the results for practice, policy, and future research.	Yes
**OTHER INFORMATION**			

Section and Topic	Item #	Checklist item	Location where item is reported
Registration and protocol	24a	Provide registration information for the review, including register name and registration number, or state that the review was not registered.	Yes
	24b	Indicate where the review protocol can be accessed, or state that a protocol was not prepared.	Yes
	24c	Describe and explain any amendments to information provided at registration or in the protocol.	No described
Support	25	Describe sources of financial or non-financial support for the review, and the role of the funders or sponsors in the review.	No described
Competing interests	26	Declare any competing interests of review authors.	No described
Availability of data, code and other materials	27	Report which of the following are publicly available and where they can be found: template data collection forms; data extracted from included studies; data used for all analyses; analytic code; any other materials used in the review.	Yes

From: Page MJ, McKenzie JE, Bossuyt PM, Boutron I, Hoffmann TC, Mulrow CD, et al. The PRISMA 2020 statement: an updated guideline for reporting systematic reviews. BMJ 2021;372:n71. doi: 10.1136/bmj.n71

For more information, visit: http://www.prisma-statement.org/

Artikel 17

Section and Topic	Item #	Checklist item	Location where item is reported
**TITLE**			
Title	1	Identify the report as a systematic review.	The Impact of Orem's Self-care Model on the Quality of Life in Patients with Type II Diabetes
**ABSTRACT**			
Abstract	2	See the PRISMA 2020 for Abstracts checklist.	The authors has well described it
**INTRODUCTION**			
Rationale	3	Describe the rationale for the review in the context of existing knowledge.	Yes
Objectives	4	Provide an explicit statement of the objective(s) or question(s) the review addresses.	Yes
**METHODS**			
Eligibility criteria	5	Specify the inclusion and exclusion criteria for the review and how studies were grouped for the syntheses.	Yes
Information sources	6	Specify all databases, registers, websites, organisations, reference lists and other sources searched or consulted to identify studies. Specify the date when each source was last searched or consulted.	Yes
Search strategy	7	Present the full search strategies for all databases, registers and websites, including any filters and limits used.	Yes
Selection process	8	Specify the methods used to decide whether a study met the inclusion criteria of the review, including how many reviewers screened each record and each report retrieved, whether they worked independently, and if applicable, details of automation tools used in the process.	Yes
Data collection process	9	Specify the methods used to collect data from reports, including how many reviewers collected data from each report, whether they worked independently, any processes for obtaining or confirming data from study investigators, and if applicable, details of automation tools used in the process.	Yes
Data items	10a	List and define all outcomes for which data were sought. Specify whether all results that were compatible with each outcome domain in each study were sought (e.g. for all measures, time points, analyses), and if not, the methods used to decide which results to collect.	Yes
	10b	List and define all other variables for which data were sought (e.g. partic-	Yes

Section and Topic	Item #	Checklist item	Location where item is reported
		ipant and intervention characteristics, funding sources). Describe any assumptions made about any missing or unclear information.	
Study risk of bias assessment	11	Specify the methods used to assess risk of bias in the included studies, including details of the tool(s) used, how many reviewers assessed each study and whether they worked independently, and if applicable, details of automation tools used in the process.	No
Effect measures	12	Specify for each outcome the effect measure(s) (e.g. risk ratio, mean difference) used in the synthesis or presentation of results.	Yes
Synthesis methods	13a	Describe the processes used to decide which studies were eligible for each synthesis (e.g. tabulating the study intervention characteristics and comparing against the planned groups for each synthesis (item #5)).	Yes
	13b	Describe any methods required to prepare the data for presentation or synthesis, such as handling of missing summary statistics, or data conversions.	Yes
	13c	Describe any methods used to tabulate or visually display results of individual studies and syntheses.	Yes
	13d	Describe any methods used to synthesize results and provide a rationale for the choice(s). If meta-analysis was performed, describe the model(s), method(s) to identify the presence and extent of statistical heterogeneity, and software package(s) used.	Yes
	13e	Describe any methods used to explore possible causes of heterogeneity among study results (e.g. subgroup analysis, meta-regression).	No
	13f	Describe any sensitivity analyses conducted to assess robustness of the synthesized results.	Yes
Reporting bias assessment	14	Describe any methods used to assess risk of bias due to missing results in a synthesis (arising from reporting biases).	No
Certainty assessment	15	Describe any methods used to assess certainty (or confidence) in the body of evidence for an outcome.	Yes
**RESULTS**			
Study selection	16a	Describe the results of the search and selection process, from the number of records identified in the search to the number of studies included in the review, ideally using a flow diagram.	Yes
	16b	Cite studies that might appear to meet the inclusion criteria, but which were excluded, and explain why they were excluded.	Yes
Study characteristics	17	Cite each included study and present its characteristics.	Yes
Risk of bias in studies	18	Present assessments of risk of bias for each included study.	No
Results of individual studies	19	For all outcomes, present, for each study: (a) summary statistics for each group (where appropriate) and (b) an effect estimate and its precision (e.g. confidence/credible interval), ideally using structured tables or plots.	Yes
Results of syntheses	20a	For each synthesis, briefly summarise the characteristics and risk of bias among contributing studies.	Yes
	20b	Present results of all statistical syntheses conducted. If meta-analysis was done, present for each the summary estimate and its precision (e.g. confidence/credible interval) and measures of statistical heterogeneity. If comparing groups, describe the direction of the effect.	No
	20c	Present results of all investigations of possible causes of heterogeneity among study results.	Yes
	20d	Present results of all sensitivity analyses conducted to assess the robustness of the synthesized results.	Yes
Reporting biases	21	Present assessments of risk of bias due to missing results (arising from reporting biases) for each synthesis assessed.	No

Section and Topic	Item #	Checklist item	Location where item is reported
Certainty of evidence	22	Present assessments of certainty (or confidence) in the body of evidence for each outcome assessed.	Yes
**DISCUSSION**			
Discussion	23a	Provide a general interpretation of the results in the context of other evidence.	Yes
	23b	Discuss any limitations of the evidence included in the review.	Yes
	23c	Discuss any limitations of the review processes used.	Yes
	23d	Discuss implications of the results for practice, policy, and future research.	No
**OTHER INFORMATION**			
Registration and protocol	24a	Provide registration information for the review, including register name and registration number, or state that the review was not registered.	Yes
	24b	Indicate where the review protocol can be accessed, or state that a protocol was not prepared.	Yes
	24c	Describe and explain any amendments to information provided at registration or in the protocol.	No described
Support	25	Describe sources of financial or non-financial support for the review, and the role of the funders or sponsors in the review.	No described
Competing interests	26	Declare any competing interests of review authors.	No
Availability of data, code and other materials	27	Report which of the following are publicly available and where they can be found: template data collection forms; data extracted from included studies; data used for all analyses; analytic code; any other materials used in the review.	Yes

From: Page MJ, McKenzie JE, Bossuyt PM, Boutron I, Hoffmann TC, Mulrow CD, et al. The PRISMA 2020 statement: an updated guideline for reporting systematic reviews. BMJ 2021;372:n71. doi: 10.1136/bmj.n71

For more information, visit: http://www.prisma-statement.org/

Artikel 18.

Section and Topic	Item #	Checklist item	Location where item is reported
**TITLE**			
Title	1	Identify the report as a systematic review.	The Effect of Yoga and Peer Support Interventions on the Quality of Life of Women with Diabetes: Results of a Randomized Controlled Trial
**ABSTRACT**			
Abstract	2	See the PRISMA 2020 for Abstracts checklist.	The authors has well described it
**INTRODUCTION**			
Rationale	3	Describe the rationale for the review in the context of existing knowledge.	Yes
Objectives	4	Provide an explicit statement of the objective(s) or question(s) the review addresses.	Yes
**METHODS**			
Eligibility criteria	5	Specify the inclusion and exclusion criteria for the review and how studies were grouped for the syntheses.	Yes
Information sources	6	Specify all databases, registers, websites, organisations, reference lists and other sources searched or consulted to identify studies. Specify the date when each source was last searched or consulted.	Yes
Search strategy	7	Present the full search strategies for all databases, registers and websites, including any filters and limits used.	Yes
Selection process	8	Specify the methods used to decide whether a study met the inclusion criteria of the review, including how many reviewers screened each record and each report retrieved, whether they	Yes

Section and Topic	Item #	Checklist item	Location where item is reported
		worked independently, and if applicable, details of automation tools used in the process.	
Data collection process	9	Specify the methods used to collect data from reports, including how many reviewers collected data from each report, whether they worked independently, any processes for obtaining or confirming data from study investigators, and if applicable, details of automation tools used in the process.	Yes
Data items	10a	List and define all outcomes for which data were sought. Specify whether all results that were compatible with each outcome domain in each study were sought (e.g. for all measures, time points, analyses), and if not, the methods used to decide which results to collect.	Yes
	10b	List and define all other variables for which data were sought (e.g. participant and intervention characteristics, funding sources). Describe any assumptions made about any missing or unclear information.	Yes
Study risk of bias assessment	11	Specify the methods used to assess risk of bias in the included studies, including details of the tool(s) used, how many reviewers assessed each study and whether they worked independently, and if applicable, details of automation tools used in the process.	No
Effect measures	12	Specify for each outcome the effect measure(s) (e.g. risk ratio, mean difference) used in the synthesis or presentation of results.	Yes
Synthesis methods	13a	Describe the processes used to decide which studies were eligible for each synthesis (e.g. tabulating the study intervention characteristics and comparing against the planned groups for each synthesis (item #5)).	Yes
	13b	Describe any methods required to prepare the data for presentation or synthesis, such as handling of missing summary statistics, or data conversions.	Yes
	13c	Describe any methods used to tabulate or visually display results of individual studies and syntheses.	Yes
	13d	Describe any methods used to synthesize results and provide a rationale for the choice(s). If meta-analysis was performed, describe the model(s), method(s) to identify the presence and extent of statistical heterogeneity, and software package(s) used.	No
	13e	Describe any methods used to explore possible causes of heterogeneity among study results (e.g. subgroup analysis, meta-regression).	Yes
	13f	Describe any sensitivity analyses conducted to assess robustness of the synthesized results.	Yes
Reporting bias assessment	14	Describe any methods used to assess risk of bias due to missing results in a synthesis (arising from reporting biases).	No
Certainty assessment	15	Describe any methods used to assess certainty (or confidence) in the body of evidence for an outcome.	Yes
**RESULTS**			
Study selection	16a	Describe the results of the search and selection process, from the number of records identified in the search to the number of studies included in the review, ideally using a flow diagram.	Yes
	16b	Cite studies that might appear to meet the inclusion criteria, but which were excluded, and explain why they were excluded.	Yes
Study characteristics	17	Cite each included study and present its characteristics.	
Risk of bias in studies	18	Present assessments of risk of bias for each included study.	No

Section and Topic	Item #	Checklist item	Location where item is reported
Results of individual studies	19	For all outcomes, present, for each study: (a) summary statistics for each group (where appropriate) and (b) an effect estimate and its precision (e.g. confidence/credible interval), ideally using structured tables or plots.	Yes
Results of syntheses	20a	For each synthesis, briefly summarise the characteristics and risk of bias among contributing studies.	Yes
	20b	Present results of all statistical syntheses conducted. If meta-analysis was done, present for each the summary estimate and its precision (e.g. confidence/credible interval) and measures of statistical heterogeneity. If comparing groups, describe the direction of the effect.	Yes
	20c	Present results of all investigations of possible causes of heterogeneity among study results.	Yes
	20d	Present results of all sensitivity analyses conducted to assess the robustness of the synthesized results.	Yes
Reporting biases	21	Present assessments of risk of bias due to missing results (arising from reporting biases) for each synthesis assessed.	No
Certainty of evidence	22	Present assessments of certainty (or confidence) in the body of evidence for each outcome assessed.	Yes
**DISCUSSION**			
Discussion	23a	Provide a general interpretation of the results in the context of other evidence.	Yes
	23b	Discuss any limitations of the evidence included in the review.	Yes
	23c	Discuss any limitations of the review processes used.	Yes
	23d	Discuss implications of the results for practice, policy, and future research.	Yes
**OTHER INFORMATION**			
Registration and protocol	24a	Provide registration information for the review, including register name and registration number, or state that the review was not registered.	Yes
	24b	Indicate where the review protocol can be accessed, or state that a protocol was not prepared.	Yes
	24c	Describe and explain any amendments to information provided at registration or in the protocol.	No described
Support	25	Describe sources of financial or non-financial support for the review, and the role of the funders or sponsors in the review.	No described
Competing interests	26	Declare any competing interests of review authors.	Yes
Availability of data, code and other materials	27	Report which of the following are publicly available and where they can be found: template data collection forms; data extracted from included studies; data used for all analyses; analytic code; any other materials used in the review.	Yes

From: Page MJ, McKenzie JE, Bossuyt PM, Boutron I, Hoffmann TC, Mulrow CD, et al. The PRISMA 2020 statement: an updated guideline for reporting systematic reviews. BMJ 2021;372:n71. doi: 10.1136/bmj.n71

For more information, visit: http://www.prisma-statement.org/

Artikel 19.

Section and Topic	Item #	Checklist item	Location where item is reported
**TITLE**			
Title	1	Identify the report as a systematic review.	Profile and predictors of health related quality of life among type II diabetes mellitus patients in Quetta city, Pakistan.

Section and Topic	Item #	Checklist item	Location where item is reported
**ABSTRACT**			
Abstract	2	See the PRISMA 2020 for Abstracts checklist.	the authors has well described it
**INTRODUCTION**			
Rationale	3	Describe the rationale for the review in the context of existing knowledge.	yes
Objectives	4	Provide an explicit statement of the objective(s) or question(s) the review addresses.	yes
**METHODS**			
Eligibility criteria	5	Specify the inclusion and exclusion criteria for the review and how studies were grouped for the syntheses.	yes
Information sources	6	Specify all databases, registers, websites, organisations, reference lists and other sources searched or consulted to identify studies. Specify the date when each source was last searched or consulted.	yes
Search strategy	7	Present the full search strategies for all databases, registers and websites, including any filters and limits used.	yes
Selection process	8	Specify the methods used to decide whether a study met the inclusion criteria of the review, including how many reviewers screened each record and each report retrieved, whether they worked independently, and if applicable, details of automation tools used in the process.	yes
Data collection process	9	Specify the methods used to collect data from reports, including how many reviewers collected data from each report, whether they worked independently, any processes for obtaining or confirming data from study investigators, and if applicable, details of automation tools used in the process.	no
Data items	10a	List and define all outcomes for which data were sought. Specify whether all results that were compatible with each outcome domain in each study were sought (e.g. for all measures, time points, analyses), and if not, the methods used to decide which results to collect.	yes
	10b	List and define all other variables for which data were sought (e.g. participant and intervention characteristics, funding sources). Describe any assumptions made about any missing or unclear information.	yes
Study risk of bias assessment	11	Specify the methods used to assess risk of bias in the included studies, including details of the tool(s) used, how many reviewers assessed each study and whether they worked independently, and if applicable, details of automation tools used in the process.	no
Effect measures	12	Specify for each outcome the effect measure(s) (e.g. risk ratio, mean difference) used in the synthesis or presentation of results.	yes
Synthesis methods	13a	Describe the processes used to decide which studies were eligible for each synthesis (e.g. tabulating the study intervention characteristics and comparing against the planned groups for each synthesis (item #5)).	yes
	13b	Describe any methods required to prepare the data for presentation or synthesis, such as handling of missing summary statistics, or data conversions.	yes
	13c	Describe any methods used to tabulate or visually display results of individual studies and syntheses.	yes
	13d	Describe any methods used to synthesize results and provide a rationale for the choice(s). If meta-analysis was performed, describe the model(s), method(s) to identify the presence and extent of statistical heterogeneity, and software package(s) used.	no
	13e	Describe any methods used to explore possible causes of heterogeneity among study results (e.g. subgroup analysis, meta-regression).	yes
	13f	Describe any sensitivity analyses conducted to assess robustness of the synthesized results.	yes

Section and Topic	Item #	Checklist item	Location where item is reported
Reporting bias assessment	14	Describe any methods used to assess risk of bias due to missing results in a synthesis (arising from reporting biases).	no
Certainty assessment	15	Describe any methods used to assess certainty (or confidence) in the body of evidence for an outcome.	yes
**RESULTS**			
Study selection	16a	Describe the results of the search and selection process, from the number of records identified in the search to the number of studies included in the review, ideally using a flow diagram.	yes
	16b	Cite studies that might appear to meet the inclusion criteria, but which were excluded, and explain why they were excluded.	yes
Study characteristics	17	Cite each included study and present its characteristics.	yes
Risk of bias in studies	18	Present assessments of risk of bias for each included study.	no
Results of individual studies	19	For all outcomes, present, for each study: (a) summary statistics for each group (where appropriate) and (b) an effect estimate and its precision (e.g. confidence/credible interval), ideally using structured tables or plots.	yes
Results of syntheses	20a	For each synthesis, briefly summarise the characteristics and risk of bias among contributing studies.	yes
	20b	Present results of all statistical syntheses conducted. If meta-analysis was done, present for each the summary estimate and its precision (e.g. confidence/credible interval) and measures of statistical heterogeneity. If comparing groups, describe the direction of the effect.	no
	20c	Present results of all investigations of possible causes of heterogeneity among study results.	yes
	20d	Present results of all sensitivity analyses conducted to assess the robustness of the synthesized results.	yes
Reporting biases	21	Present assessments of risk of bias due to missing results (arising from reporting biases) for each synthesis assessed.	no
Certainty of evidence	22	Present assessments of certainty (or confidence) in the body of evidence for each outcome assessed.	yes
**DISCUSSION**			
Discussion	23a	Provide a general interpretation of the results in the context of other evidence.	yes
	23b	Discuss any limitations of the evidence included in the review.	yes
	23c	Discuss any limitations of the review processes used.	yes
	23d	Discuss implications of the results for practice, policy, and future research.	yes
**OTHER INFORMATION**			
Registration and protocol	24a	Provide registration information for the review, including register name and registration number, or state that the review was not registered.	yes
	24b	Indicate where the review protocol can be accessed, or state that a protocol was not prepared.	no described
	24c	Describe and explain any amendments to information provided at registration or in the protocol.	
Support	25	Describe sources of financial or non-financial support for the review, and the role of the funders or sponsors in the review.	no described
Competing interests	26	Declare any competing interests of review authors.	yes
Availability of data, code and other ma-	27	Report which of the following are publicly available and where they can be found: template data collection forms; data extracted from in-	yes

Section and Topic	Item #	Checklist item	Location where item is reported
terials		cluded studies; data used for all analyses; analytic code; any other materials used in the review.	

From: Page MJ, McKenzie JE, Bossuyt PM, Boutron I, Hoffmann TC, Mulrow CD, et al. The PRISMA 2020 statement: an updated guideline for reporting systematic reviews. BMJ 2021;372:n71. doi: 10.1136/bmj.n71
For more information, visit: http://www.prisma-statement.org/

Artikel 20

Section and Topic	Item #	Checklist item	Location where item is reported
**TITLE**			
Title	1	Identify the report as a systematic review.	Aromatherapy Massage for Neuropathic Pain and Quality of Life in Diabetic Patients
**ABSTRACT**			
Abstract	2	See the PRISMA 2020 for Abstracts checklist.	The authors has well describe it
**INTRODUCTION**			
Rationale	3	Describe the rationale for the review in the context of existing knowledge.	Yes
Objectives	4	Provide an explicit statement of the objective(s) or question(s) the review addresses.	Yes
**METHODS**			
Eligibility criteria	5	Specify the inclusion and exclusion criteria for the review and how studies were grouped for the syntheses.	Yes
Information sources	6	Specify all databases, registers, websites, organisations, reference lists and other sources searched or consulted to identify studies. Specify the date when each source was last searched or consulted.	Yes
Search strategy	7	Present the full search strategies for all databases, registers and websites, including any filters and limits used.	Yes
Selection process	8	Specify the methods used to decide whether a study met the inclusion criteria of the review, including how many reviewers screened each record and each report retrieved, whether they worked independently, and if applicable, details of automation tools used in the process.	Yes
Data collection process	9	Specify the methods used to collect data from reports, including how many reviewers collected data from each report, whether they worked independently, any processes for obtaining or confirming data from study investigators, and if applicable, details of automation tools used in the process.	Yes
Data items	10a	List and define all outcomes for which data were sought. Specify whether all results that were compatible with each outcome domain in each study were sought (e.g. for all measures, time points, analyses), and if not, the methods used to decide which results to collect.	Yes
	10b	List and define all other variables for which data were sought (e.g. participant and intervention characteristics, funding sources). Describe any assumptions made about any missing or unclear information.	Yes
Study risk of bias assessment	11	Specify the methods used to assess risk of bias in the included studies, including details of the tool(s) used, how many reviewers assessed each study and whether they worked independently, and if applicable, details of automation tools used in the process.	No
Effect measures	12	Specify for each outcome the effect measure(s) (e.g. risk ratio, mean difference) used in the synthesis or presentation of results.	Yes
Synthesis methods	13a	Describe the processes used to decide which studies were eligible for each synthesis (e.g. tabulating the study intervention characteristics and comparing against the planned groups for each synthesis (item #5)).	Yes

Section and Topic	Item #	Checklist item	Location where item is reported
	13b	Describe any methods required to prepare the data for presentation or synthesis, such as handling of missing summary statistics, or data conversions.	Yes
	13c	Describe any methods used to tabulate or visually display results of individual studies and syntheses.	Yes
	13d	Describe any methods used to synthesize results and provide a rationale for the choice(s). If meta-analysis was performed, describe the model(s), method(s) to identify the presence and extent of statistical heterogeneity, and software package(s) used.	Yes
	13e	Describe any methods used to explore possible causes of heterogeneity among study results (e.g. subgroup analysis, meta-regression).	Yes
	13f	Describe any sensitivity analyses conducted to assess robustness of the synthesized results.	Yes
Reporting bias assessment	14	Describe any methods used to assess risk of bias due to missing results in a synthesis (arising from reporting biases).	No
Certainty assessment	15	Describe any methods used to assess certainty (or confidence) in the body of evidence for an outcome.	Yes
**RESULTS**			
Study selection	16a	Describe the results of the search and selection process, from the number of records identified in the search to the number of studies included in the review, ideally using a flow diagram.	Yes
	16b	Cite studies that might appear to meet the inclusion criteria, but which were excluded, and explain why they were excluded.	Yes
Study characteristics	17	Cite each included study and present its characteristics.	Yes
Risk of bias in studies	18	Present assessments of risk of bias for each included study.	No
Results of individual studies	19	For all outcomes, present, for each study: (a) summary statistics for each group (where appropriate) and (b) an effect estimate and its precision (e.g. confidence/credible interval), ideally using structured tables or plots.	Yes
Results of syntheses	20a	For each synthesis, briefly summarise the characteristics and risk of bias among contributing studies.	Yes
	20b	Present results of all statistical syntheses conducted. If meta-analysis was done, present for each the summary estimate and its precision (e.g. confidence/credible interval) and measures of statistical heterogeneity. If comparing groups, describe the direction of the effect.	Yes
	20c	Present results of all investigations of possible causes of heterogeneity among study results.	Yes
	20d	Present results of all sensitivity analyses conducted to assess the robustness of the synthesized results.	Yes
Reporting biases	21	Present assessments of risk of bias due to missing results (arising from reporting biases) for each synthesis assessed.	No
Certainty of evidence	22	Present assessments of certainty (or confidence) in the body of evidence for each outcome assessed.	Yes
**DISCUSSION**			
Discussion	23a	Provide a general interpretation of the results in the context of other evidence.	Yes
	23b	Discuss any limitations of the evidence included in the review.	Yes
	23c	Discuss any limitations of the review processes used.	Yes
	23d	Discuss implications of the results for practice, policy, and future research.	Yes

Section and Topic	Item #	Checklist item	Location where item is reported
**OTHER INFORMATION**			
Registration and protocol	24a	Provide registration information for the review, including register name and registration number, or state that the review was not registered.	Yes
	24b	Indicate where the review protocol can be accessed, or state that a protocol was not prepared.	Yes
	24c	Describe and explain any amendments to information provided at registration or in the protocol.	No described
Support	25	Describe sources of financial or non-financial support for the review, and the role of the funders or sponsors in the review.	No described
Competing interests	26	Declare any competing interests of review authors.	Yes
Availability of data, code and other materials	27	Report which of the following are publicly available and where they can be found: template data collection forms; data extracted from included studies; data used for all analyses; analytic code; any other materials used in the review.	Yes

From: Page MJ, McKenzie JE, Bossuyt PM, Boutron I, Hoffmann TC, Mulrow CD, et al. The PRISMA 2020 statement: an updated guideline for reporting systematic reviews. BMJ 2021;372:n71. doi: 10.1136/bmj.n71
For more information, visit: http://www.prisma-statement.org/

Artikel 21.

Section and Topic	Item #	Checklist item	Location where item is reported
**TITLE**			
Title	1	Identify the report as a systematic review.	Effect Of The Consultation Of Nursing On Knowledge, Quality Of Life, Attitude Towards Disease And Self-Care Among Persons With Diabetes
**ABSTRACT**			
Abstract	2	See the PRISMA 2020 for Abstracts checklist.	The authors has well described it
**INTRODUCTION**			
Rationale	3	Describe the rationale for the review in the context of existing knowledge.	Yes
Objectives	4	Provide an explicit statement of the objective(s) or question(s) the review addresses.	Yes
**METHODS**			
Eligibility criteria	5	Specify the inclusion and exclusion criteria for the review and how studies were grouped for the syntheses.	Yes
Information sources	6	Specify all databases, registers, websites, organisations, reference lists and other sources searched or consulted to identify studies. Specify the date when each source was last searched or consulted.	Yes
Search strategy	7	Present the full search strategies for all databases, registers and websites, including any filters and limits used.	Yes
Selection process	8	Specify the methods used to decide whether a study met the inclusion criteria of the review, including how many reviewers screened each record and each report retrieved, whether they worked independently, and if applicable, details of automation tools used in the process.	Yes
Data collection process	9	Specify the methods used to collect data from reports, including how many reviewers collected data from each report, whether they worked independently, any processes for obtaining or confirming data from study investigators, and if applicable, details of automation tools used in the process.	Yes
Data items	10a	List and define all outcomes for which data were sought. Specify whether all results that were compatible with each outcome domain in each study were sought (e.g. for all measures, time points,	Yes

Section and Topic	Item #	Checklist item	Location where item is reported
		analyses), and if not, the methods used to decide which results to collect.	
	10b	List and define all other variables for which data were sought (e.g. participant and intervention characteristics, funding sources). Describe any assumptions made about any missing or unclear information.	Yes
Study risk of bias assessment	11	Specify the methods used to assess risk of bias in the included studies, including details of the tool(s) used, how many reviewers assessed each study and whether they worked independently, and if applicable, details of automation tools used in the process.	No
Effect measures	12	Specify for each outcome the effect measure(s) (e.g. risk ratio, mean difference) used in the synthesis or presentation of results.	Yes
Synthesis methods	13a	Describe the processes used to decide which studies were eligible for each synthesis (e.g. tabulating the study intervention characteristics and comparing against the planned groups for each synthesis (item #5)).	Yes
	13b	Describe any methods required to prepare the data for presentation or synthesis, such as handling of missing summary statistics, or data conversions.	Yes
	13c	Describe any methods used to tabulate or visually display results of individual studies and syntheses.	Yes
	13d	Describe any methods used to synthesize results and provide a rationale for the choice(s). If meta-analysis was performed, describe the model(s), method(s) to identify the presence and extent of statistical heterogeneity, and software package(s) used.	Yes
	13e	Describe any methods used to explore possible causes of heterogeneity among study results (e.g. subgroup analysis, meta-regression).	Yes
	13f	Describe any sensitivity analyses conducted to assess robustness of the synthesized results.	Yes
Reporting bias assessment	14	Describe any methods used to assess risk of bias due to missing results in a synthesis (arising from reporting biases).	No
Certainty assessment	15	Describe any methods used to assess certainty (or confidence) in the body of evidence for an outcome.	Yes
**RESULTS**			
Study selection	16a	Describe the results of the search and selection process, from the number of records identified in the search to the number of studies included in the review, ideally using a flow diagram.	Yes
	16b	Cite studies that might appear to meet the inclusion criteria, but which were excluded, and explain why they were excluded.	Yes
Study characteristics	17	Cite each included study and present its characteristics.	Yes
Risk of bias in studies	18	Present assessments of risk of bias for each included study.	No
Results of individual studies	19	For all outcomes, present, for each study: (a) summary statistics for each group (where appropriate) and (b) an effect estimate and its precision (e.g. confidence/credible interval), ideally using structured tables or plots.	Yes
Results of syntheses	20a	For each synthesis, briefly summarise the characteristics and risk of bias among contributing studies.	Yes
	20b	Present results of all statistical syntheses conducted. If meta-analysis was done, present for each the summary estimate and its precision (e.g. confidence/credible interval) and measures of statistical heterogeneity. If comparing groups, describe the direction	No

Section and Topic	Item #	Checklist item	Location where item is reported
		of the effect.	
	20c	Present results of all investigations of possible causes of heterogeneity among study results.	Yes
	20d	Present results of all sensitivity analyses conducted to assess the robustness of the synthesized results.	Yes
Reporting biases	21	Present assessments of risk of bias due to missing results (arising from reporting biases) for each synthesis assessed.	No
Certainty of evidence	22	Present assessments of certainty (or confidence) in the body of evidence for each outcome assessed.	Yes
**DISCUSSION**			
Discussion	23a	Provide a general interpretation of the results in the context of other evidence.	Yes
	23b	Discuss any limitations of the evidence included in the review.	Yes
	23c	Discuss any limitations of the review processes used.	Yes
	23d	Discuss implications of the results for practice, policy, and future research.	Yes
**OTHER INFORMATION**			
Registration and protocol	24a	Provide registration information for the review, including register name and registration number, or state that the review was not registered.	Yes
	24b	Indicate where the review protocol can be accessed, or state that a protocol was not prepared.	Yes
	24c	Describe and explain any amendments to information provided at registration or in the protocol.	No described
Support	25	Describe sources of financial or non-financial support for the review, and the role of the funders or sponsors in the review.	No described
Competing interests	26	Declare any competing interests of review authors.	Yes
Availability of data, code and other materials	27	Report which of the following are publicly available and where they can be found: template data collection forms; data extracted from included studies; data used for all analyses; analytic code; any other materials used in the review.	Yes

From: Page MJ, McKenzie JE, Bossuyt PM, Boutron I, Hoffmann TC, Mulrow CD, et al. The PRISMA 2020 statement: an updated guideline for reporting systematic reviews. BMJ 2021;372:n71. doi: 10.1136/bmj.n71

For more information, visit: http://www.prisma-statement.org/

Artikel 22.

Section and Topic	Item #	Checklist item	Location where item is reported
**TITLE**			
Title	1	Identify the report as a systematic review.	Analysis of the Relationship of Long Suffering with Diabetes Mellitus with Quality of Life of People with Diabetes Mellitus
**ABSTRACT**			
Abstract	2	See the PRISMA 2020 for Abstracts checklist.	The authors has well described it
**INTRODUCTION**			
Rationale	3	Describe the rationale for the review in the context of existing knowledge.	Yes
Objectives	4	Provide an explicit statement of the objective(s) or question(s) the review addresses.	Yes
**METHODS**			
Eligibility criteria	5	Specify the inclusion and exclusion criteria for the review and how	Yes

Section and Topic	Item #	Checklist item	Location where item is reported
		studies were grouped for the syntheses.	
Information sources	6	Specify all databases, registers, websites, organisations, reference lists and other sources searched or consulted to identify studies. Specify the date when each source was last searched or consulted.	Yes
Search strategy	7	Present the full search strategies for all databases, registers and websites, including any filters and limits used.	No
Selection process	8	Specify the methods used to decide whether a study met the inclusion criteria of the review, including how many reviewers screened each record and each report retrieved, whether they worked independently, and if applicable, details of automation tools used in the process.	Yes
Data collection process	9	Specify the methods used to collect data from reports, including how many reviewers collected data from each report, whether they worked independently, any processes for obtaining or confirming data from study investigators, and if applicable, details of automation tools used in the process.	No
Data items	10a	List and define all outcomes for which data were sought. Specify whether all results that were compatible with each outcome domain in each study were sought (e.g. for all measures, time points, analyses), and if not, the methods used to decide which results to collect.	Yes
	10b	List and define all other variables for which data were sought (e.g. participant and intervention characteristics, funding sources). Describe any assumptions made about any missing or unclear information.	Yes
Study risk of bias assessment	11	Specify the methods used to assess risk of bias in the included studies, including details of the tool(s) used, how many reviewers assessed each study and whether they worked independently, and if applicable, details of automation tools used in the process.	No
Effect measures	12	Specify for each outcome the effect measure(s) (e.g. risk ratio, mean difference) used in the synthesis or presentation of results.	Yes
Synthesis methods	13a	Describe the processes used to decide which studies were eligible for each synthesis (e.g. tabulating the study intervention characteristics and comparing against the planned groups for each synthesis (item #5)).	Yes
	13b	Describe any methods required to prepare the data for presentation or synthesis, such as handling of missing summary statistics, or data conversions.	No
	13c	Describe any methods used to tabulate or visually display results of individual studies and syntheses.	Yes
	13d	Describe any methods used to synthesize results and provide a rationale for the choice(s). If meta-analysis was performed, describe the model(s), method(s) to identify the presence and extent of statistical heterogeneity, and software package(s) used.	No
	13e	Describe any methods used to explore possible causes of heterogeneity among study results (e.g. subgroup analysis, meta-regression).	No
	13f	Describe any sensitivity analyses conducted to assess robustness of the synthesized results.	Yes
Reporting bias assessment	14	Describe any methods used to assess risk of bias due to missing results in a synthesis (arising from reporting biases).	No
Certainty assessment	15	Describe any methods used to assess certainty (or confidence) in the body of evidence for an outcome.	Yes
**RESULTS**			
Study selection	16a	Describe the results of the search and selection process, from the number of records identified in the search to the number of studies included in the review, ideally using a flow diagram.	Yes

Section and Topic	Item #	Checklist item	Location where item is reported
	16b	Cite studies that might appear to meet the inclusion criteria, but which were excluded, and explain why they were excluded.	Yes
Study characteristics	17	Cite each included study and present its characteristics.	Yes
Risk of bias in studies	18	Present assessments of risk of bias for each included study.	No
Results of individual studies	19	For all outcomes, present, for each study: (a) summary statistics for each group (where appropriate) and (b) an effect estimate and its precision (e.g. confidence/credible interval), ideally using structured tables or plots.	Yes
Results of syntheses	20a	For each synthesis, briefly summarise the characteristics and risk of bias among contributing studies.	Yes
	20b	Present results of all statistical syntheses conducted. If meta-analysis was done, present for each the summary estimate and its precision (e.g. confidence/credible interval) and measures of statistical heterogeneity. If comparing groups, describe the direction of the effect.	No
	20c	Present results of all investigations of possible causes of heterogeneity among study results.	Yes
	20d	Present results of all sensitivity analyses conducted to assess the robustness of the synthesized results.	Yes
Reporting biases	21	Present assessments of risk of bias due to missing results (arising from reporting biases) for each synthesis assessed.	No
Certainty of evidence	22	Present assessments of certainty (or confidence) in the body of evidence for each outcome assessed.	Yes
**DISCUSSION**			
Discussion	23a	Provide a general interpretation of the results in the context of other evidence.	Yes
	23b	Discuss any limitations of the evidence included in the review.	Yes
	23c	Discuss any limitations of the review processes used.	Yes
	23d	Discuss implications of the results for practice, policy, and future research.	No
**OTHER INFORMATION**			
Registration and protocol	24a	Provide registration information for the review, including register name and registration number, or state that the review was not registered.	Yes
	24b	Indicate where the review protocol can be accessed, or state that a protocol was not prepared.	Yes
	24c	Describe and explain any amendments to information provided at registration or in the protocol.	No described
Support	25	Describe sources of financial or non-financial support for the review, and the role of the funders or sponsors in the review.	No described
Competing interests	26	Declare any competing interests of review authors.	No
Availability of data, code and other materials	27	Report which of the following are publicly available and where they can be found: template data collection forms; data extracted from included studies; data used for all analyses; analytic code; any other materials used in the review.	Yes

From: Page MJ, McKenzie JE, Bossuyt PM, Boutron I, Hoffmann TC, Mulrow CD, et al. The PRISMA 2020 statement: an updated guideline for reporting systematic reviews. BMJ 2021;372:n71. doi: 10.1136/bmj.n71

For more information, visit: http://www.prisma-statement.org/

Artikel 23

Section and Topic	Item #	Checklist item	Location where item is reported

Section and Topic	Item #	Checklist item	Location where item is reported
**TITLE**			
Title	1	Identify the report as a systematic review.	Effect of exercise on the quality of life in type 2 diabetes mellitus.
**ABSTRACT**			
Abstract	2	See the PRISMA 2020 for Abstracts checklist.	The authors has well described it
**INTRODUCTION**			
Rationale	3	Describe the rationale for the review in the context of existing knowledge.	Yes
Objectives	4	Provide an explicit statement of the objective(s) or question(s) the review addresses.	Yes
**METHODS**			
Eligibility criteria	5	Specify the inclusion and exclusion criteria for the review and how studies were grouped for the syntheses.	Yes
Information sources	6	Specify all databases, registers, websites, organisations, reference lists and other sources searched or consulted to identify studies. Specify the date when each source was last searched or consulted.	Yes
Search strategy	7	Present the full search strategies for all databases, registers and websites, including any filters and limits used.	Yes
Selection process	8	Specify the methods used to decide whether a study met the inclusion criteria of the review, including how many reviewers screened each record and each report retrieved, whether they worked independently, and if applicable, details of automation tools used in the process.	Yes
Data collection process	9	Specify the methods used to collect data from reports, including how many reviewers collected data from each report, whether they worked independently, any processes for obtaining or confirming data from study investigators, and if applicable, details of automation tools used in the process.	Yes
Data items	10a	List and define all outcomes for which data were sought. Specify whether all results that were compatible with each outcome domain in each study were sought (e.g. for all measures, time points, analyses), and if not, the methods used to decide which results to collect.	Yes
	10b	List and define all other variables for which data were sought (e.g. participant and intervention characteristics, funding sources). Describe any assumptions made about any missing or unclear information.	Yes
Study risk of bias assessment	11	Specify the methods used to assess risk of bias in the included studies, including details of the tool(s) used, how many reviewers assessed each study and whether they worked independently, and if applicable, details of automation tools used in the process.	No
Effect measures	12	Specify for each outcome the effect measure(s) (e.g. risk ratio, mean difference) used in the synthesis or presentation of results.	Yes
Synthesis methods	13a	Describe the processes used to decide which studies were eligible for each synthesis (e.g. tabulating the study intervention characteristics and comparing against the planned groups for each synthesis (item #5)).	Yes
	13b	Describe any methods required to prepare the data for presentation or synthesis, such as handling of missing summary statistics, or data conversions.	Yes
	13c	Describe any methods used to tabulate or visually display results of individual studies and syntheses.	Yes
	13d	Describe any methods used to synthesize results and provide a rationale for the choice(s). If meta-analysis was performed, describe the model(s), method(s) to identify the presence and extent of statistical heterogeneity, and software package(s) used.	Yes
	13e	Describe any methods used to explore possible causes of heterogeneity among study results (e.g. subgroup analysis, meta-regression).	Yes

Section and Topic	Item #	Checklist item	Location where item is reported
	13f	Describe any sensitivity analyses conducted to assess robustness of the synthesized results.	Yes
Reporting bias assessment	14	Describe any methods used to assess risk of bias due to missing results in a synthesis (arising from reporting biases).	No
Certainty assessment	15	Describe any methods used to assess certainty (or confidence) in the body of evidence for an outcome.	Yes
**RESULTS**			
Study selection	16a	Describe the results of the search and selection process, from the number of records identified in the search to the number of studies included in the review, ideally using a flow diagram.	Yes
	16b	Cite studies that might appear to meet the inclusion criteria, but which were excluded, and explain why they were excluded.	Yes
Study characteristics	17	Cite each included study and present its characteristics.	Yes
Risk of bias in studies	18	Present assessments of risk of bias for each included study.	No
Results of individual studies	19	For all outcomes, present, for each study: (a) summary statistics for each group (where appropriate) and (b) an effect estimate and its precision (e.g. confidence/credible interval), ideally using structured tables or plots.	Yes
Results of syntheses	20a	For each synthesis, briefly summarise the characteristics and risk of bias among contributing studies.	Yes
	20b	Present results of all statistical syntheses conducted. If meta-analysis was done, present for each the summary estimate and its precision (e.g. confidence/credible interval) and measures of statistical heterogeneity. If comparing groups, describe the direction of the effect.	Yes
	20c	Present results of all investigations of possible causes of heterogeneity among study results.	Yes
	20d	Present results of all sensitivity analyses conducted to assess the robustness of the synthesized results.	Yes
Reporting biases	21	Present assessments of risk of bias due to missing results (arising from reporting biases) for each synthesis assessed.	No
Certainty of evidence	22	Present assessments of certainty (or confidence) in the body of evidence for each outcome assessed.	Yes
**DISCUSSION**			
Discussion	23a	Provide a general interpretation of the results in the context of other evidence.	Yes
	23b	Discuss any limitations of the evidence included in the review.	Yes
	23c	Discuss any limitations of the review processes used.	Yes
	23d	Discuss implications of the results for practice, policy, and future research.	Yes
**OTHER INFORMATION**			
Registration and protocol	24a	Provide registration information for the review, including register name and registration number, or state that the review was not registered.	Yes
	24b	Indicate where the review protocol can be accessed, or state that a protocol was not prepared.	Yes
	24c	Describe and explain any amendments to information provided at registration or in the protocol.	No described
Support	25	Describe sources of financial or non-financial support for the review, and the role of the funders or sponsors in the review.	No described
Competing interests	26	Declare any competing interests of review authors.	Yes
Availability of data, code and other materials	27	Report which of the following are publicly available and where they can be found: template data collection forms; data extracted from included studies; data used for all analyses; analytic code; any other materials used in the review.	Yes

From: Page MJ, McKenzie JE, Bossuyt PM, Boutron I, Hoffmann TC, Mulrow CD, et al. The PRISMA 2020 statement: an updated guideline for reporting systematic reviews. BMJ 2021;372:n71. doi: 10.1136/bmj.n71
For more information, visit: http://www.prisma-statement.org/

Artikel 24

Section and Topic	Item #	Checklist item	Location where item is reported
**TITLE**			
Title	1	Identify the report as a systematic review.	The effect of diabetes knowledge and attitudes on self-management and quality of life among people with type 2 diabetes
**ABSTRACT**			
Abstract	2	See the PRISMA 2020 for Abstracts checklist.	The authors has well described it
**INTRODUCTION**			
Rationale	3	Describe the rationale for the review in the context of existing knowledge.	Yes
Objectives	4	Provide an explicit statement of the objective(s) or question(s) the review addresses.	Yes
**METHODS**			
Eligibility criteria	5	Specify the inclusion and exclusion criteria for the review and how studies were grouped for the syntheses.	Yes
Information sources	6	Specify all databases, registers, websites, organisations, reference lists and other sources searched or consulted to identify studies. Specify the date when each source was last searched or consulted.	Yes
Search strategy	7	Present the full search strategies for all databases, registers and websites, including any filters and limits used.	Yes
Selection process	8	Specify the methods used to decide whether a study met the inclusion criteria of the review, including how many reviewers screened each record and each report retrieved, whether they worked independently, and if applicable, details of automation tools used in the process.	Yes
Data collection process	9	Specify the methods used to collect data from reports, including how many reviewers collected data from each report, whether they worked independently, any processes for obtaining or confirming data from study investigators, and if applicable, details of automation tools used in the process.	Yes
Data items	10a	List and define all outcomes for which data were sought. Specify whether all results that were compatible with each outcome domain in each study were sought (e.g. for all measures, time points, analyses), and if not, the methods used to decide which results to collect.	No
	10b	List and define all other variables for which data were sought (e.g. participant and intervention characteristics, funding sources). Describe any assumptions made about any missing or unclear information.	Yes
Study risk of bias assessment	11	Specify the methods used to assess risk of bias in the included studies, including details of the tool(s) used, how many reviewers assessed each study and whether they worked independently, and if applicable, details of automation tools used in the process.	No
Effect measures	12	Specify for each outcome the effect measure(s) (e.g. risk ratio, mean difference) used in the synthesis or presentation of results.	Yes
Synthesis methods	13a	Describe the processes used to decide which studies were eligible for each synthesis (e.g. tabulating the study intervention characteristics and comparing against the planned groups for each synthesis (item #5)).	Yes

Section and Topic	Item #	Checklist item	Location where item is reported
	13b	Describe any methods required to prepare the data for presentation or synthesis, such as handling of missing summary statistics, or data conversions.	Yes
	13c	Describe any methods used to tabulate or visually display results of individual studies and syntheses.	Yes
	13d	Describe any methods used to synthesize results and provide a rationale for the choice(s). If meta-analysis was performed, describe the model(s), method(s) to identify the presence and extent of statistical heterogeneity, and software package(s) used.	No
	13e	Describe any methods used to explore possible causes of heterogeneity among study results (e.g. subgroup analysis, meta-regression).	No
	13f	Describe any sensitivity analyses conducted to assess robustness of the synthesized results.	No
Reporting bias assessment	14	Describe any methods used to assess risk of bias due to missing results in a synthesis (arising from reporting biases).	No
Certainty assessment	15	Describe any methods used to assess certainty (or confidence) in the body of evidence for an outcome.	Yes
**RESULTS**			
Study selection	16a	Describe the results of the search and selection process, from the number of records identified in the search to the number of studies included in the review, ideally using a flow diagram.	Yes
	16b	Cite studies that might appear to meet the inclusion criteria, but which were excluded, and explain why they were excluded.	Yes
Study characteristics	17	Cite each included study and present its characteristics.	Yes
Risk of bias in studies	18	Present assessments of risk of bias for each included study.	No
Results of individual studies	19	For all outcomes, present, for each study: (a) summary statistics for each group (where appropriate) and (b) an effect estimate and its precision (e.g. confidence/credible interval), ideally using structured tables or plots.	Yes
Results of syntheses	20a	For each synthesis, briefly summarise the characteristics and risk of bias among contributing studies.	Yes
	20b	Present results of all statistical syntheses conducted. If meta-analysis was done, present for each the summary estimate and its precision (e.g. confidence/credible interval) and measures of statistical heterogeneity. If comparing groups, describe the direction of the effect.	No
	20c	Present results of all investigations of possible causes of heterogeneity among study results.	Yes
	20d	Present results of all sensitivity analyses conducted to assess the robustness of the synthesized results.	Yes
Reporting biases	21	Present assessments of risk of bias due to missing results (arising from reporting biases) for each synthesis assessed.	Yes
Certainty of evidence	22	Present assessments of certainty (or confidence) in the body of evidence for each outcome assessed.	Yes
**DISCUSSION**			
Discussion	23a	Provide a general interpretation of the results in the context of other evidence.	Yes
	23b	Discuss any limitations of the evidence included in the review.	Yes
	23c	Discuss any limitations of the review processes used.	Yes
	23d	Discuss implications of the results for practice, policy, and future research.	No

Section and Topic	Item #	Checklist item	Location where item is reported
**OTHER INFORMATION**			
Registration and protocol	24a	Provide registration information for the review, including register name and registration number, or state that the review was not registered.	Yes
	24b	Indicate where the review protocol can be accessed, or state that a protocol was not prepared.	Yes
	24c	Describe and explain any amendments to information provided at registration or in the protocol.	No described
Support	25	Describe sources of financial or non-financial support for the review, and the role of the funders or sponsors in the review.	No described
Competing interests	26	Declare any competing interests of review authors.	No
Availability of data, code and other materials	27	Report which of the following are publicly available and where they can be found: template data collection forms; data extracted from included studies; data used for all analyses; analytic code; any other materials used in the review.	No

From: Page MJ, McKenzie JE, Bossuyt PM, Boutron I, Hoffmann TC, Mulrow CD, et al. The PRISMA 2020 statement: an updated guideline for reporting systematic reviews. BMJ 2021;372:n71. doi: 10.1136/bmj.n71

For more information, visit: http://www.prisma-statement.org/

Artikel 25

Section and Topic	Item #	Checklist item	Location where item is reported
**TITLE**			
Title	1	Identify the report as a systematic review.	A cross-sectional assessment of health-related quality of life among type 2 diabetes patients in Riyadh, Saudi Arabia
**ABSTRACT**			
Abstract	2	See the PRISMA 2020 for Abstracts checklist.	The authors has well described it
**INTRODUCTION**			
Rationale	3	Describe the rationale for the review in the context of existing knowledge.	Yes
Objectives	4	Provide an explicit statement of the objective(s) or question(s) the review addresses.	Yes
**METHODS**			
Eligibility criteria	5	Specify the inclusion and exclusion criteria for the review and how studies were grouped for the syntheses.	Yes
Information sources	6	Specify all databases, registers, websites, organisations, reference lists and other sources searched or consulted to identify studies. Specify the date when each source was last searched or consulted.	Yes
Search strategy	7	Present the full search strategies for all databases, registers and websites, including any filters and limits used.	No
Selection process	8	Specify the methods used to decide whether a study met the inclusion criteria of the review, including how many reviewers screened each record and each report retrieved, whether they worked independently, and if applicable, details of automation tools used in the process.	Yes
Data collection process	9	Specify the methods used to collect data from reports, including how many reviewers collected data from each report, whether they worked independently, any processes for obtaining or confirming data from study investigators, and if applicable, details of automation tools used in the process.	Yes
Data items	10a	List and define all outcomes for which data were sought. Specify whether all results that were compatible with each outcome domain in	Yes

Section and Topic	Item #	Checklist item	Location where item is reported
		each study were sought (e.g. for all measures, time points, analyses), and if not, the methods used to decide which results to collect.	
	10b	List and define all other variables for which data were sought (e.g. participant and intervention characteristics, funding sources). Describe any assumptions made about any missing or unclear information.	Yes
Study risk of bias assessment	11	Specify the methods used to assess risk of bias in the included studies, including details of the tool(s) used, how many reviewers assessed each study and whether they worked independently, and if applicable, details of automation tools used in the process.	No
Effect measures	12	Specify for each outcome the effect measure(s) (e.g. risk ratio, mean difference) used in the synthesis or presentation of results.	Yes
Synthesis methods	13a	Describe the processes used to decide which studies were eligible for each synthesis (e.g. tabulating the study intervention characteristics and comparing against the planned groups for each synthesis (item #5)).	Yes
	13b	Describe any methods required to prepare the data for presentation or synthesis, such as handling of missing summary statistics, or data conversions.	Yes
	13c	Describe any methods used to tabulate or visually display results of individual studies and syntheses.	Yes
	13d	Describe any methods used to synthesize results and provide a rationale for the choice(s). If meta-analysis was performed, describe the model(s), method(s) to identify the presence and extent of statistical heterogeneity, and software package(s) used.	Yes
	13e	Describe any methods used to explore possible causes of heterogeneity among study results (e.g. subgroup analysis, meta-regression).	No
	13f	Describe any sensitivity analyses conducted to assess robustness of the synthesized results.	Yes
Reporting bias assessment	14	Describe any methods used to assess risk of bias due to missing results in a synthesis (arising from reporting biases).	No
Certainty assessment	15	Describe any methods used to assess certainty (or confidence) in the body of evidence for an outcome.	Yes
**RESULTS**			
Study selection	16a	Describe the results of the search and selection process, from the number of records identified in the search to the number of studies included in the review, ideally using a flow diagram.	Yes
	16b	Cite studies that might appear to meet the inclusion criteria, but which were excluded, and explain why they were excluded.	Yes
Study characteristics	17	Cite each included study and present its characteristics.	Yes
Risk of bias in studies	18	Present assessments of risk of bias for each included study.	No
Results of individual studies	19	For all outcomes, present, for each study: (a) summary statistics for each group (where appropriate) and (b) an effect estimate and its precision (e.g. confidence/credible interval), ideally using structured tables or plots.	Yes
Results of syntheses	20a	For each synthesis, briefly summarise the characteristics and risk of bias among contributing studies.	No
	20b	Present results of all statistical syntheses conducted. If meta-analysis was done, present for each the summary estimate and its precision (e.g. confidence/credible interval) and measures of statistical heterogeneity. If comparing groups, describe the direction of the effect.	No
	20c	Present results of all investigations of possible causes of heterogeneity among study results.	Yes

Section and Topic	Item #	Checklist item	Location where item is reported
	20d	Present results of all sensitivity analyses conducted to assess the robustness of the synthesized results.	Yes
Reporting biases	21	Present assessments of risk of bias due to missing results (arising from reporting biases) for each synthesis assessed.	No
Certainty of evidence	22	Present assessments of certainty (or confidence) in the body of evidence for each outcome assessed.	Yes
**DISCUSSION**			
Discussion	23a	Provide a general interpretation of the results in the context of other evidence.	Yes
	23b	Discuss any limitations of the evidence included in the review.	Yes
	23c	Discuss any limitations of the review processes used.	Yes
	23d	Discuss implications of the results for practice, policy, and future research.	No
**OTHER INFORMATION**			
Registration and protocol	24a	Provide registration information for the review, including register name and registration number, or state that the review was not registered.	Yes
	24b	Indicate where the review protocol can be accessed, or state that a protocol was not prepared.	Yes
	24c	Describe and explain any amendments to information provided at registration or in the protocol.	No
Support	25	Describe sources of financial or non-financial support for the review, and the role of the funders or sponsors in the review.	No
Competing interests	26	Declare any competing interests of review authors.	Yes
Availability of data, code and other materials	27	Report which of the following are publicly available and where they can be found: template data collection forms; data extracted from included studies; data used for all analyses; analytic code; any other materials used in the review.	Yes

From: Page MJ, McKenzie JE, Bossuyt PM, Boutron I, Hoffmann TC, Mulrow CD, et al. The PRISMA 2020 statement: an updated guideline for reporting systematic reviews. BMJ 2021;372:n71. doi: 10.1136/bmj.n71

For more information, visit: http://www.prisma-statement.org/

Artikel 26

Section and Topic	Item #	Checklist item	Location where item is reported
**TITLE**			
Title	1	Identify the report as a systematic review.	Effects of diabetes self-management education program on lowering blood glucose level, stress, and quality of life among females with type 2 diabetes mellitus in Thailand
**ABSTRACT**			
Abstract	2	See the PRISMA 2020 for Abstracts checklist.	The authors well has described it
**INTRODUCTION**			
Rationale	3	Describe the rationale for the review in the context of existing knowledge.	Yes
Objectives	4	Provide an explicit statement of the objective(s) or question(s) the review addresses.	Yes
**METHODS**			
Eligibility criteria	5	Specify the inclusion and exclusion criteria for the review and	Yes

Section and Topic	Item #	Checklist item	Location where item is reported
		how studies were grouped for the syntheses.	
Information sources	6	Specify all databases, registers, websites, organisations, reference lists and other sources searched or consulted to identify studies. Specify the date when each source was last searched or consulted.	Yes
Search strategy	7	Present the full search strategies for all databases, registers and websites, including any filters and limits used.	Yes
Selection process	8	Specify the methods used to decide whether a study met the inclusion criteria of the review, including how many reviewers screened each record and each report retrieved, whether they worked independently, and if applicable, details of automation tools used in the process.	Yes
Data collection process	9	Specify the methods used to collect data from reports, including how many reviewers collected data from each report, whether they worked independently, any processes for obtaining or confirming data from study investigators, and if applicable, details of automation tools used in the process.	Yes
Data items	10a	List and define all outcomes for which data were sought. Specify whether all results that were compatible with each outcome domain in each study were sought (e.g. for all measures, time points, analyses), and if not, the methods used to decide which results to collect.	Yes
	10b	List and define all other variables for which data were sought (e.g. participant and intervention characteristics, funding sources). Describe any assumptions made about any missing or unclear information.	Yes
Study risk of bias assessment	11	Specify the methods used to assess risk of bias in the included studies, including details of the tool(s) used, how many reviewers assessed each study and whether they worked independently, and if applicable, details of automation tools used in the process.	No
Effect measures	12	Specify for each outcome the effect measure(s) (e.g. risk ratio, mean difference) used in the synthesis or presentation of results.	Yes
Synthesis methods	13a	Describe the processes used to decide which studies were eligible for each synthesis (e.g. tabulating the study intervention characteristics and comparing against the planned groups for each synthesis (item #5)).	Yes
	13b	Describe any methods required to prepare the data for presentation or synthesis, such as handling of missing summary statistics, or data conversions.	Yes
	13c	Describe any methods used to tabulate or visually display results of individual studies and syntheses.	Yes
	13d	Describe any methods used to synthesize results and provide a rationale for the choice(s). If meta-analysis was performed, describe the model(s), method(s) to identify the presence and extent of statistical heterogeneity, and software package(s) used.	Yes
	13e	Describe any methods used to explore possible causes of heterogeneity among study results (e.g. subgroup analysis, meta-regression).	Yes
	13f	Describe any sensitivity analyses conducted to assess robustness of the synthesized results.	Yes

Section and Topic	Item #	Checklist item	Location where item is reported
Reporting bias assessment	14	Describe any methods used to assess risk of bias due to missing results in a synthesis (arising from reporting biases).	No
Certainty assessment	15	Describe any methods used to assess certainty (or confidence) in the body of evidence for an outcome.	Yes
**RESULTS**			
Study selection	16a	Describe the results of the search and selection process, from the number of records identified in the search to the number of studies included in the review, ideally using a flow diagram.	Yes
	16b	Cite studies that might appear to meet the inclusion criteria, but which were excluded, and explain why they were excluded.	Yes
Study characteristics	17	Cite each included study and present its characteristics.	Yes
Risk of bias in studies	18	Present assessments of risk of bias for each included study.	No
Results of individual studies	19	For all outcomes, present, for each study: (a) summary statistics for each group (where appropriate) and (b) an effect estimate and its precision (e.g. confidence/credible interval), ideally using structured tables or plots.	Yes
Results of syntheses	20a	For each synthesis, briefly summarise the characteristics and risk of bias among contributing studies.	Yes
	20b	Present results of all statistical syntheses conducted. If meta-analysis was done, present for each the summary estimate and its precision (e.g. confidence/credible interval) and measures of statistical heterogeneity. If comparing groups, describe the direction of the effect.	Yes
	20c	Present results of all investigations of possible causes of heterogeneity among study results.	Yes
	20d	Present results of all sensitivity analyses conducted to assess the robustness of the synthesized results.	Yes
Reporting biases	21	Present assessments of risk of bias due to missing results (arising from reporting biases) for each synthesis assessed.	No
Certainty of evidence	22	Present assessments of certainty (or confidence) in the body of evidence for each outcome assessed.	Yes
**DISCUSSION**			
Discussion	23a	Provide a general interpretation of the results in the context of other evidence.	Yes
	23b	Discuss any limitations of the evidence included in the review.	Yes
	23c	Discuss any limitations of the review processes used.	Yes
	23d	Discuss implications of the results for practice, policy, and future research.	Yes
**OTHER INFORMATION**			
Registration and protocol	24a	Provide registration information for the review, including register name and registration number, or state that the review was not registered.	Yes
	24b	Indicate where the review protocol can be accessed, or state that a protocol was not prepared.	Yes
	24c	Describe and explain any amendments to information provided	No described

Section and Topic	Item #	Checklist item	Location where item is reported
		at registration or in the protocol.	
Support	25	Describe sources of financial or non-financial support for the review, and the role of the funders or sponsors in the review.	No described
Competing interests	26	Declare any competing interests of review authors.	Yes
Availability of data, code and other materials	27	Report which of the following are publicly available and where they can be found: template data collection forms; data extracted from included studies; data used for all analyses; analytic code; any other materials used in the review.	Yes

From: Page MJ, McKenzie JE, Bossuyt PM, Boutron I, Hoffmann TC, Mulrow CD, et al. The PRISMA 2020 statement: an updated guideline for reporting systematic reviews. BMJ 2021;372:n71. doi: 10.1136/bmj.n71
For more information, visit: http://www.prisma-statement.org/

Artikel 27.

Section and Topic	Item #	Checklist item	Location where item is reported
**TITLE**			
Title	1	Identify the report as a systematic review.	Impact of diabetes self-management, diabetes management self-efficacy and diabetes knowledge on glycemic control in people with Type 2 Diabetes (T2D): A multicenter study in Thailand
**ABSTRACT**			
Abstract	2	See the PRISMA 2020 for Abstracts checklist.	The author has well described it
**INTRODUCTION**			
Rationale	3	Describe the rationale for the review in the context of existing knowledge.	Yes
Objectives	4	Provide an explicit statement of the objective(s) or question(s) the review addresses.	Yes
**METHODS**			
Eligibility criteria	5	Specify the inclusion and exclusion criteria for the review and how studies were grouped for the syntheses.	Yes
Information sources	6	Specify all databases, registers, websites, organisations, reference lists and other sources searched or consulted to identify studies. Specify the date when each source was last searched or consulted.	Yes
Search strategy	7	Present the full search strategies for all databases, registers and websites, including any filters and limits used.	Yes
Selection process	8	Specify the methods used to decide whether a study met the inclusion criteria of the review, including how many reviewers screened each record and each report retrieved, whether they worked independently, and if applicable, details of automation tools used in the process.	Yes
Data collection process	9	Specify the methods used to collect data from reports, including how many reviewers collected data from each report, whether they worked independently, any processes for obtaining or confirming data from study investigators, and if applicable, details of automation tools used in the process.	Yes
Data items	10a	List and define all outcomes for which data were sought. Specify whether all results that were compatible with each outcome domain in each study were sought (e.g. for all measures, time points, analyses), and if not, the methods used to decide which results to collect.	Yes
	10b	List and define all other variables for which data were sought (e.g. participant and intervention characteristics, funding	Yes

Section and Topic	Item #	Checklist item	Location where item is reported
		sources). Describe any assumptions made about any missing or unclear information.	
Study risk of bias assessment	11	Specify the methods used to assess risk of bias in the included studies, including details of the tool(s) used, how many reviewers assessed each study and whether they worked independently, and if applicable, details of automation tools used in the process.	No
Effect measures	12	Specify for each outcome the effect measure(s) (e.g. risk ratio, mean difference) used in the synthesis or presentation of results.	Yes
Synthesis methods	13a	Describe the processes used to decide which studies were eligible for each synthesis (e.g. tabulating the study intervention characteristics and comparing against the planned groups for each synthesis (item #5)).	Yes
	13b	Describe any methods required to prepare the data for presentation or synthesis, such as handling of missing summary statistics, or data conversions.	Yes
	13c	Describe any methods used to tabulate or visually display results of individual studies and syntheses.	Yes
	13d	Describe any methods used to synthesize results and provide a rationale for the choice(s). If meta-analysis was performed, describe the model(s), method(s) to identify the presence and extent of statistical heterogeneity, and software package(s) used.	Yes
	13e	Describe any methods used to explore possible causes of heterogeneity among study results (e.g. subgroup analysis, meta-regression).	No
	13f	Describe any sensitivity analyses conducted to assess robustness of the synthesized results.	Yes
Reporting bias assessment	14	Describe any methods used to assess risk of bias due to missing results in a synthesis (arising from reporting biases).	No
Certainty assessment	15	Describe any methods used to assess certainty (or confidence) in the body of evidence for an outcome.	Yes
**RESULTS**			
Study selection	16a	Describe the results of the search and selection process, from the number of records identified in the search to the number of studies included in the review, ideally using a flow diagram.	Yes
	16b	Cite studies that might appear to meet the inclusion criteria, but which were excluded, and explain why they were excluded.	Yes
Study characteristics	17	Cite each included study and present its characteristics.	Yes
Risk of bias in studies	18	Present assessments of risk of bias for each included study.	No
Results of individual studies	19	For all outcomes, present, for each study: (a) summary statistics for each group (where appropriate) and (b) an effect estimate and its precision (e.g. confidence/credible interval), ideally using structured tables or plots.	Yes
Results of syntheses	20a	For each synthesis, briefly summarise the characteristics and risk of bias among contributing studies.	No
	20b	Present results of all statistical syntheses conducted. If meta-analysis was done, present for each the summary estimate and its precision (e.g. confidence/credible interval) and measures of statistical heterogeneity. If comparing groups, describe the	Yes

Section and Topic	Item #	Checklist item	Location where item is reported
		direction of the effect.	
	20c	Present results of all investigations of possible causes of heterogeneity among study results.	Yes
	20d	Present results of all sensitivity analyses conducted to assess the robustness of the synthesized results.	Yes
Reporting biases	21	Present assessments of risk of bias due to missing results (arising from reporting biases) for each synthesis assessed.	No
Certainty of evidence	22	Present assessments of certainty (or confidence) in the body of evidence for each outcome assessed.	Yes
**DISCUSSION**			
Discussion	23a	Provide a general interpretation of the results in the context of other evidence.	Yes
	23b	Discuss any limitations of the evidence included in the review.	Yes
	23c	Discuss any limitations of the review processes used.	Yes
	23d	Discuss implications of the results for practice, policy, and future research.	No
**OTHER INFORMATION**			
Registration and protocol	24a	Provide registration information for the review, including register name and registration number, or state that the review was not registered.	Yes
	24b	Indicate where the review protocol can be accessed, or state that a protocol was not prepared.	Yes
	24c	Describe and explain any amendments to information provided at registration or in the protocol.	No
Support	25	Describe sources of financial or non-financial support for the review, and the role of the funders or sponsors in the review.	No
Competing interests	26	Declare any competing interests of review authors.	Yes
Availability of data, code and other materials	27	Report which of the following are publicly available and where they can be found: template data collection forms; data extracted from included studies; data used for all analyses; analytic code; any other materials used in the review.	Yes

From: Page MJ, McKenzie JE, Bossuyt PM, Boutron I, Hoffmann TC, Mulrow CD, et al. The PRISMA 2020 statement: an updated guideline for reporting systematic reviews. BMJ 2021;372:n71. doi: 10.1136/bmj.n71

For more information, visit: http://www.prisma-statement.org/

Artikel 28.

Section and Topic	Item #	Checklist item	Location where item is reported
**TITLE**			
Title	1	Identify the report as a systematic review.	SMS-based reminder system design to improve medication adherence of diabetes mellitus patients
**ABSTRACT**			
Abstract	2	See the PRISMA 2020 for Abstracts checklist.	The authors has well described it
**INTRODUCTION**			
Rationale	3	Describe the rationale for the review in the context of existing knowledge.	Yes

Section and Topic	Item #	Checklist item	Location where item is reported
Objectives	4	Provide an explicit statement of the objective(s) or question(s) the review addresses.	Yes
**METHODS**			
Eligibility criteria	5	Specify the inclusion and exclusion criteria for the review and how studies were grouped for the syntheses.	Yes
Information sources	6	Specify all databases, registers, websites, organisations, reference lists and other sources searched or consulted to identify studies. Specify the date when each source was last searched or consulted.	Yes
Search strategy	7	Present the full search strategies for all databases, registers and websites, including any filters and limits used.	Yes
Selection process	8	Specify the methods used to decide whether a study met the inclusion criteria of the review, including how many reviewers screened each record and each report retrieved, whether they worked independently, and if applicable, details of automation tools used in the process.	Yes
Data collection process	9	Specify the methods used to collect data from reports, including how many reviewers collected data from each report, whether they worked independently, any processes for obtaining or confirming data from study investigators, and if applicable, details of automation tools used in the process.	Yes
Data items	10a	List and define all outcomes for which data were sought. Specify whether all results that were compatible with each outcome domain in each study were sought (e.g. for all measures, time points, analyses), and if not, the methods used to decide which results to collect.	Yes
	10b	List and define all other variables for which data were sought (e.g. participant and intervention characteristics, funding sources). Describe any assumptions made about any missing or unclear information.	Yes
Study risk of bias assessment	11	Specify the methods used to assess risk of bias in the included studies, including details of the tool(s) used, how many reviewers assessed each study and whether they worked independently, and if applicable, details of automation tools used in the process.	No
Effect measures	12	Specify for each outcome the effect measure(s) (e.g. risk ratio, mean difference) used in the synthesis or presentation of results.	Yes
Synthesis methods	13a	Describe the processes used to decide which studies were eligible for each synthesis (e.g. tabulating the study intervention characteristics and comparing against the planned groups for each synthesis (item #5)).	Yes
	13b	Describe any methods required to prepare the data for presentation or synthesis, such as handling of missing summary statistics, or data conversions.	Yes
	13c	Describe any methods used to tabulate or visually display results of individual studies and syntheses.	Yes
	13d	Describe any methods used to synthesize results and provide a rationale for the choice(s). If meta-analysis was performed, describe the model(s), method(s) to identify the presence and extent of statistical heterogeneity, and software package(s) used.	Yes
	13e	Describe any methods used to explore possible causes of heterogeneity among study results (e.g. subgroup analysis, meta-regression).	Yes
	13f	Describe any sensitivity analyses conducted to assess robustness of the synthesized results.	Yes
Reporting bias assessment	14	Describe any methods used to assess risk of bias due to missing results in a synthesis (arising from reporting biases).	No
Certainty assessment	15	Describe any methods used to assess certainty (or confidence) in the body of evidence for an outcome.	Yes
**RESULTS**			
Study selection	16a	Describe the results of the search and selection process, from the number	Yes

Section and Topic	Item #	Checklist item	Location where item is reported
		of records identified in the search to the number of studies included in the review, ideally using a flow diagram.	
	16b	Cite studies that might appear to meet the inclusion criteria, but which were excluded, and explain why they were excluded.	Yes
Study characteristics	17	Cite each included study and present its characteristics.	Yes
Risk of bias in studies	18	Present assessments of risk of bias for each included study.	No
Results of individual studies	19	For all outcomes, present, for each study: (a) summary statistics for each group (where appropriate) and (b) an effect estimate and its precision (e.g. confidence/credible interval), ideally using structured tables or plots.	Yes
Results of syntheses	20a	For each synthesis, briefly summarise the characteristics and risk of bias among contributing studies.	Yes
	20b	Present results of all statistical syntheses conducted. If meta-analysis was done, present for each the summary estimate and its precision (e.g. confidence/credible interval) and measures of statistical heterogeneity. If comparing groups, describe the direction of the effect.	No
	20c	Present results of all investigations of possible causes of heterogeneity among study results.	Yes
	20d	Present results of all sensitivity analyses conducted to assess the robustness of the synthesized results.	Yes
Reporting biases	21	Present assessments of risk of bias due to missing results (arising from reporting biases) for each synthesis assessed.	Yes
Certainty of evidence	22	Present assessments of certainty (or confidence) in the body of evidence for each outcome assessed.	Yes
**DISCUSSION**			
Discussion	23a	Provide a general interpretation of the results in the context of other evidence.	Yes
	23b	Discuss any limitations of the evidence included in the review.	Yes
	23c	Discuss any limitations of the review processes used.	Yes
	23d	Discuss implications of the results for practice, policy, and future research.	No
**OTHER INFORMATION**			
Registration and protocol	24a	Provide registration information for the review, including register name and registration number, or state that the review was not registered.	Yes
	24b	Indicate where the review protocol can be accessed, or state that a protocol was not prepared.	Yes
	24c	Describe and explain any amendments to information provided at registration or in the protocol.	No
Support	25	Describe sources of financial or non-financial support for the review, and the role of the funders or sponsors in the review.	No
Competing interests	26	Declare any competing interests of review authors.	Yes
Availability of data, code and other materials	27	Report which of the following are publicly available and where they can be found: template data collection forms; data extracted from included studies; data used for all analyses; analytic code; any other materials used in the review.	Yes

From: Page MJ, McKenzie JE, Bossuyt PM, Boutron I, Hoffmann TC, Mulrow CD, et al. The PRISMA 2020 statement: an updated guideline for reporting systematic reviews. BMJ 2021;372:n71. doi: 10.1136/bmj.n71

For more information, visit: http://www.prisma-statement.org/

Artikel 29.

Section and Topic	Item #	Checklist item	Location where item is reported
**TITLE**			
Title	1	Identify the report as a systematic review.	Diabetes Self-management Education and Support in Adults With Type 2 Diabetes: A Consensus Report of the American Diabetes Association, the Association of Diabetes Care and Education Specialists, the Academy of Nutrition and Dietetics, the American Academy of Family Physicians, the American Academy of PAs, the American Association of Nurse Practitioners, and the American Pharmacists Association.
**ABSTRACT**			
Abstract	2	See the PRISMA 2020 for Abstracts checklist.	The authors has well described it
**INTRODUCTION**			
Rationale	3	Describe the rationale for the review in the context of existing knowledge.	Yes
Objectives	4	Provide an explicit statement of the objective(s) or question(s) the review addresses.	Yes
**METHODS**			
Eligibility criteria	5	Specify the inclusion and exclusion criteria for the review and how studies were grouped for the syntheses.	Yes
Information sources	6	Specify all databases, registers, websites, organisations, reference lists and other sources searched or consulted to identify studies. Specify the date when each source was last searched or consulted.	Yes
Search strategy	7	Present the full search strategies for all databases, registers and websites, including any filters and limits used.	Yes
Selection process	8	Specify the methods used to decide whether a study met the inclusion criteria of the review, including how many reviewers screened each record and each report retrieved, whether they worked independently, and if applicable, details of automation tools used in the process.	Yes
Data collection process	9	Specify the methods used to collect data from reports, including how many reviewers collected data from each report, whether they worked independently, any processes for obtaining or confirming data from study investigators, and if applicable, details of automation tools used in the process.	Yes
Data items	10a	List and define all outcomes for which data were sought. Specify whether all results that were compatible with each outcome domain in each study were sought (e.g. for all measures, time points, analyses), and if not, the methods used to decide which results to collect.	No
	10b	List and define all other variables for which data were sought (e.g. participant and intervention characteristics, funding sources). Describe any assumptions made about any missing or unclear information.	Yes
Study risk of bias assessment	11	Specify the methods used to assess risk of bias in the included studies, including details of the tool(s) used, how many reviewers assessed each study and whether they worked independently, and if applicable, details of automation tools used in the process.	No

Section and Topic	Item #	Checklist item	Location where item is reported
Effect measures	12	Specify for each outcome the effect measure(s) (e.g. risk ratio, mean difference) used in the synthesis or presentation of results.	Yes
Synthesis methods	13a	Describe the processes used to decide which studies were eligible for each synthesis (e.g. tabulating the study intervention characteristics and comparing against the planned groups for each synthesis (item #5)).	Yes
	13b	Describe any methods required to prepare the data for presentation or synthesis, such as handling of missing summary statistics, or data conversions.	Yes
	13c	Describe any methods used to tabulate or visually display results of individual studies and syntheses.	Yes
	13d	Describe any methods used to synthesize results and provide a rationale for the choice(s). If meta-analysis was performed, describe the model(s), method(s) to identify the presence and extent of statistical heterogeneity, and software package(s) used.	Yes
	13e	Describe any methods used to explore possible causes of heterogeneity among study results (e.g. subgroup analysis, meta-regression).	Yes
	13f	Describe any sensitivity analyses conducted to assess robustness of the synthesized results.	Yes
Reporting bias assessment	14	Describe any methods used to assess risk of bias due to missing results in a synthesis (arising from reporting biases).	No
Certainty assessment	15	Describe any methods used to assess certainty (or confidence) in the body of evidence for an outcome.	Yes
**RESULTS**			
Study selection	16a	Describe the results of the search and selection process, from the number of records identified in the search to the number of studies included in the review, ideally using a flow diagram.	Yes
	16b	Cite studies that might appear to meet the inclusion criteria, but which were excluded, and explain why they were excluded.	Yes
Study characteristics	17	Cite each included study and present its characteristics.	Yes
Risk of bias in studies	18	Present assessments of risk of bias for each included study.	No
Results of individual studies	19	For all outcomes, present, for each study: (a) summary statistics for each group (where appropriate) and (b) an effect estimate and its precision (e.g. confidence/credible interval), ideally using structured tables or plots.	Yes
Results of syntheses	20a	For each synthesis, briefly summarise the characteristics and risk of bias among contributing studies.	Yes
	20b	Present results of all statistical syntheses conducted. If meta-analysis was done, present for each the summary estimate and its precision (e.g. confidence/credible interval) and measures	No

Section and Topic	Item #	Checklist item	Location where item is reported
		of statistical heterogeneity. If comparing groups, describe the direction of the effect.	
	20c	Present results of all investigations of possible causes of heterogeneity among study results.	Yes
	20d	Present results of all sensitivity analyses conducted to assess the robustness of the synthesized results.	Yes
Reporting biases	21	Present assessments of risk of bias due to missing results (arising from reporting biases) for each synthesis assessed.	No
Certainty of evidence	22	Present assessments of certainty (or confidence) in the body of evidence for each outcome assessed.	Yes
**DISCUSSION**			
Discussion	23a	Provide a general interpretation of the results in the context of other evidence.	Yes
	23b	Discuss any limitations of the evidence included in the review.	Yes
	23c	Discuss any limitations of the review processes used.	Yes
	23d	Discuss implications of the results for practice, policy, and future research.	Yes
**OTHER INFORMATION**			
Registration and protocol	24a	Provide registration information for the review, including register name and registration number, or state that the review was not registered.	Yes
	24b	Indicate where the review protocol can be accessed, or state that a protocol was not prepared.	Yes
	24c	Describe and explain any amendments to information provided at registration or in the protocol.	No describe
Support	25	Describe sources of financial or non-financial support for the review, and the role of the funders or sponsors in the review.	No describe
Competing interests	26	Declare any competing interests of review authors.	Yes
Availability of data, code and other materials	27	Report which of the following are publicly available and where they can be found: template data collection forms; data extracted from included studies; data used for all analyses; analytic code; any other materials used in the review.	Yes

From: Page MJ, McKenzie JE, Bossuyt PM, Boutron I, Hoffmann TC, Mulrow CD, et al. The PRISMA 2020 statement: an updated guideline for reporting systematic reviews. BMJ 2021;372:n71. doi: 10.1136/bmj.n71
For more information, visit: http://www.prisma-statement.org/

Artikel 30.

Section and Topic	Item #	Checklist item	Location where item is reported
**TITLE**			
Title	1	Identify the report as a systematic review.	Lactobacillus bulgaricus improves antioxidant capacity of black garlic in the prevention of gestational diabetes mellitus: a randomized control trial.

Section and Topic	Item #	Checklist item	Location where item is reported
**ABSTRACT**			
Abstract	2	See the PRISMA 2020 for Abstracts checklist.	The authors has well describe it
**INTRODUCTION**			
Rationale	3	Describe the rationale for the review in the context of existing knowledge.	Yes
Objectives	4	Provide an explicit statement of the objective(s) or question(s) the review addresses.	Yes
**METHODS**			
Eligibility criteria	5	Specify the inclusion and exclusion criteria for the review and how studies were grouped for the syntheses.	Yes
Information sources	6	Specify all databases, registers, websites, organisations, reference lists and other sources searched or consulted to identify studies. Specify the date when each source was last searched or consulted.	Yes
Search strategy	7	Present the full search strategies for all databases, registers and websites, including any filters and limits used.	Yes
Selection process	8	Specify the methods used to decide whether a study met the inclusion criteria of the review, including how many reviewers screened each record and each report retrieved, whether they worked independently, and if applicable, details of automation tools used in the process.	Yes
Data collection process	9	Specify the methods used to collect data from reports, including how many reviewers collected data from each report, whether they worked independently, any processes for obtaining or confirming data from study investigators, and if applicable, details of automation tools used in the process.	Yes
Data items	10a	List and define all outcomes for which data were sought. Specify whether all results that were compatible with each outcome domain in each study were sought (e.g. for all measures, time points, analyses), and if not, the methods used to decide which results to collect.	Yes
	10b	List and define all other variables for which data were sought (e.g. participant and intervention characteristics, funding sources). Describe any assumptions made about any missing or unclear information.	Yes
Study risk of bias assessment	11	Specify the methods used to assess risk of bias in the included studies, including details of the tool(s) used, how many reviewers assessed each study and whether they worked independently, and if applicable, details of automation tools used in the process.	No
Effect measures	12	Specify for each outcome the effect measure(s) (e.g. risk ratio, mean difference) used in the synthesis or presentation of results.	Yes
Synthesis methods	13a	Describe the processes used to decide which studies were eligible for each synthesis (e.g. tabulating the study intervention characteristics and comparing against the planned groups for each synthesis (item #5)).	Yes
	13b	Describe any methods required to prepare the data for presentation or synthesis, such as handling of missing summary statistics, or data conversions.	Yes
	13c	Describe any methods used to tabulate or visually display results of individual studies and syntheses.	Yes
	13d	Describe any methods used to synthesize results and provide a rationale for the choice(s). If meta-analysis was performed, describe the model(s), method(s) to identify the presence and extent of statistical heterogeneity, and software package(s) used.	Yes
	13e	Describe any methods used to explore possible causes of heterogeneity among study results (e.g. subgroup analysis, meta-	Yes

Section and Topic	Item #	Checklist item	Location where item is reported
		regression).	
	13f	Describe any sensitivity analyses conducted to assess robustness of the synthesized results.	Yes
Reporting bias assessment	14	Describe any methods used to assess risk of bias due to missing results in a synthesis (arising from reporting biases).	No
Certainty assessment	15	Describe any methods used to assess certainty (or confidence) in the body of evidence for an outcome.	Yes
**RESULTS**			
Study selection	16a	Describe the results of the search and selection process, from the number of records identified in the search to the number of studies included in the review, ideally using a flow diagram.	Yes
	16b	Cite studies that might appear to meet the inclusion criteria, but which were excluded, and explain why they were excluded.	Yes
Study characteristics	17	Cite each included study and present its characteristics.	Yes
Risk of bias in studies	18	Present assessments of risk of bias for each included study.	No
Results of individual studies	19	For all outcomes, present, for each study: (a) summary statistics for each group (where appropriate) and (b) an effect estimate and its precision (e.g. confidence/credible interval), ideally using structured tables or plots.	Yes
Results of syntheses	20a	For each synthesis, briefly summarise the characteristics and risk of bias among contributing studies.	Yes
	20b	Present results of all statistical syntheses conducted. If meta-analysis was done, present for each the summary estimate and its precision (e.g. confidence/credible interval) and measures of statistical heterogeneity. If comparing groups, describe the direction of the effect.	Yes
	20c	Present results of all investigations of possible causes of heterogeneity among study results.	Yes
	20d	Present results of all sensitivity analyses conducted to assess the robustness of the synthesized results.	Yes
Reporting biases	21	Present assessments of risk of bias due to missing results (arising from reporting biases) for each synthesis assessed.	No
Certainty of evidence	22	Present assessments of certainty (or confidence) in the body of evidence for each outcome assessed.	Yes
**DISCUSSION**			
Discussion	23a	Provide a general interpretation of the results in the context of other evidence.	Yes
	23b	Discuss any limitations of the evidence included in the review.	Yes
	23c	Discuss any limitations of the review processes used.	Yes
	23d	Discuss implications of the results for practice, policy, and future research.	Yes
**OTHER INFORMATION**			
Registration and protocol	24a	Provide registration information for the review, including register name and registration number, or state that the review was not registered.	Yes
	24b	Indicate where the review protocol can be accessed, or state that a protocol was not prepared.	Yes
	24c	Describe and explain any amendments to information provided at registration or in the protocol.	No describe
Support	25	Describe sources of financial or non-financial support for the	No describe

Section and Topic	Item #	Checklist item	Location where item is reported
		review, and the role of the funders or sponsors in the review.	
Competing interests	26	Declare any competing interests of review authors.	Yes
Availability of data, code and other materials	27	Report which of the following are publicly available and where they can be found: template data collection forms; data extracted from included studies; data used for all analyses; analytic code; any other materials used in the review.	Yes

From: Page MJ, McKenzie JE, Bossuyt PM, Boutron I, Hoffmann TC, Mulrow CD, et al. The PRISMA 2020 statement: an updated guideline for reporting systematic reviews. BMJ 2021;372:n71. doi: 10.1136/bmj.n71

For more information, visit: http://www.prisma-statement.org/
